# Synthesis of Sulfonamides Incorporating Piperidinyl-Hydrazidoureido and Piperidinyl-Hydrazidothioureido Moieties and Their Carbonic Anhydrase I, II, IX and XII Inhibitory Activity

**DOI:** 10.3390/molecules27175370

**Published:** 2022-08-23

**Authors:** Davide Moi, Alessandro Deplano, Andrea Angeli, Gianfranco Balboni, Claudiu T. Supuran, Valentina Onnis

**Affiliations:** 1 Department of Life and Environmental Sciences, Unit of Pharmaceutical, Pharmacological and Nutraceutical Sciences, University of Cagliari, Monserrato University Campus, 09042 Monserrato, Italy; 2 Pharmacelera, Torre R, 4a Planta, Despatx A05, Parc Cientific de Barcelona, Baldiri Reixac 8, 08028 Barcelona, Spain; 3 Polo Scientifico Neurofarba Department, Laboratorio di Chimica Bioinorganica, Università Degli Studi di Firenze, Room 188, Via della Lastruccia 3, Sesto Fiorentino, 50019 Florence, Italy

**Keywords:** benzene sulfonamides, hydrazidoureas, hydrazidothioureaureas, carbonic anhydrase inhibitors

## Abstract

Here we report a small library of hydrazinocarbonyl-ureido and thioureido benzenesulfonamide derivatives, designed and synthesized as potent and selective human carbonic anhydrase inhibitors (hCAIs). The synthesized compounds were evaluated against isoforms hCA I, II, IX and XII using acetazolamide (AAZ) as standard inhibitor. Several urea and thiourea derivatives showed inhibitory activity at low nanomolar levels with selectivity against the cytosolic hCA II isoform, as well as the transmembrane, tumor-associated enzymes hCA IX and XII. The thiourea derivatives showed enhanced potency as compared to urea analogues. Additionally, eight compounds **5g**, **5m**, **5o**, **5q**, **6l**, **6j**, **6o** and **6u** were selected for docking analysis on isoform I, II, IX, XII to illustrate the potential interaction with the enzyme to better understand the activity against the different isoforms.

## 1. Introduction

Carbonic Anhydrase (CA) is a well-known family of metalloenzymes which is involved in the reversible conversion of CO_2_ into hydrogen carbonate ions and protons, water-soluble products that regulate the physiological pH. In the past years, CAs have been extensively studied, identifying eight different families: α, β, γ, δ, ζ, η-, θ- and ι-, of which αCAs are present in humans [1,2]. At the moment, 15 different αCAs isoforms have been distinguished, of which 12 are catalytically active: CAs I-IV, CA VA-VB, CA VI, CA VII, CA IX and CAs XII-XIV, distinguished by their different catalytic efficiencies and cellular localization [2,3]. Three of them, CA VIII, X and XI are called CA-related proteins CARPs. The active isoforms have been further clustered in four different classes differing on localization: *h*CAs I, II, III, VII, and XIII are the cytosolic isoforms, *h*CAs IV, IX, XII, and XIV are membrane-associated isoforms, *h*CAs VA and VB are predominantly expressed in mitochondria, whereas *h*CA VI is present in saliva and milk. *h*CAs are spread in several tissues and organs which several implications in physiological processes. Therefore, dysregulation of *h*CAs is related with several pathological processes such as glaucoma, epilepsy, edema, obesity and tumors [3,4,5]. All *h*CAs have in common a highly conserved active site where an Zn^2+^, is coordinated by His94, His96, His119 and by a water molecule, which is crucial for the catalytic activity [6]. Among all *h*CA, two isoforms, *h*CA IX and *h*CA, have been intensively studied as targets for the development of antiproliferative compounds, due to their role in survival of hypoxic tumors [6,7,8,9,10,11]. Cancer is generally characterized by an abnormal cell growth and spreading into neighboring tissues, but typically this overgrowing is not followed by correct vascularization with a poor oxygen and nourishment delivery as a consequence. Inevitably, this condition is related with the presence of multiple hypoxic regions which might limit the tumor progression. The hypoxia environment leads to important changes in gene expressions as an adaptive process, necessary for continual progression and metastasis, mediated by hypoxia-inducible transcription factor (HIF-1*α*), ref. [12], which promotes anaerobic glycolysis, a metabolic modifications crucial for cell survival [13]. As a result of anaerobic metabolism, a massive amount of lactic acid is present into the cytosol, with a consequent reduction in intracellular pH, which is incompatible with biochemical reaction of the cell [14]. In this context, *h*CA IX and *h*CA XII are overexpressed in cancer cells as an important tool for the control of intracellular pH, which allows tumor cells to become highly proliferative, aggressive, and resistant to numerous pharmacological therapies [15]. Therefore, the development of selective *h*CA IX and *h*CA XII inhibitors represents an appealing approach for the development of potential antiproliferative compounds. The majority of *h*CA inhibitors have been defined as zinc-binders [16,17] and among them, primary sulfonamides are the most common *h*CAi due to their unique interaction, not only with Zn^2+^, but also with the nearby residues [18,19]. As a continuation of our efforts in the design and synthesis of new potent and selective CAIs [18,19,20,21,22], this study presents a small library of benzenesulfonamide based-compounds, designed to extend the SAR piperidinyl-hydrazidoureides. The new benzenesulfonamides were endowed with a piperidine ring liked to ureido/thioureidoaryl tail and were evaluated against *h*CA I, *h*CA II, *h*CA IX, and *h*CA XII isoforms. Docking studies were also carried out to better understand the interaction with the different isoforms and confirm the acquired inhibition data.

## 2. Results

### 2.1. Chemistry

**Scheme 1:**
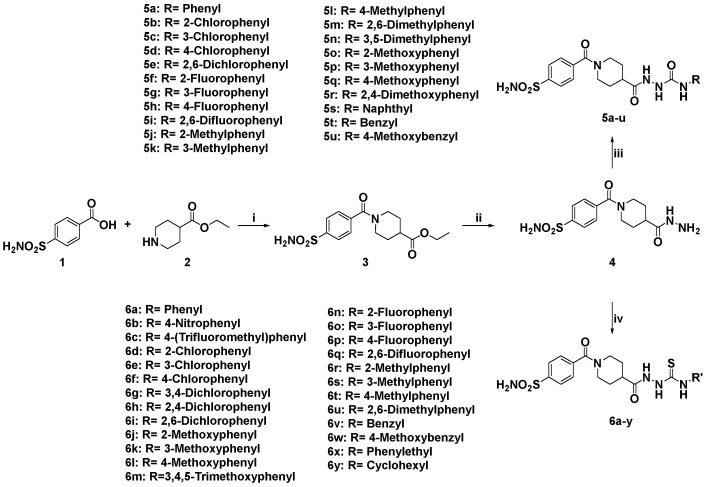
General synthetic procedure for sulfonamides subsets **5a**–**u** and **6a**–**y**. Reagents and conditions: (i) EDCI, HOBt, dry CH_3_CN r.t. 12 h, yield 77%; (ii) NH_2_NH_2_·H_2_O, absolute EtOH, reflux 3 h, yield 78%; (iii) substituted isocyanates, absolute EtOH, reflux 6 h, yield 34–91%; (iv) substituted isothiocyanates, absolute EtOH, reflux 6 h, yield 44–98%.

The target 4-sulfamoylbenzoyl-piperidine derivatives **5a**–**u** and **6a**–**y** were obtained through the synthetic pathway shown in Scheme 1. The key intermediate ethyl 1-(4-sulfamoylbenzoyl)piperidine-4-carboxylate (**3**) was prepared by amide coupling between 4-sulfamoylbenzoic acid (**1**) and ethyl piperidine-4-carboxylate (**2**). The condensation was accomplished in dry acetonitrile solution (CH_3_CN), using 1-(3-dimethylaminopropyl)-3-ethylcarbodiimide hydrochloride (EDCI) as coupling agent, in the presence of 1-hydroxybenzotriazole hydrate (HOBt). The intermediate **3** was converted in the corresponding hydrazide **4** by reaction with hydrazine hydrate in absolute ethanol (EtOH). Finally, hydrazinecarbonyl(piperidine-1-carbonyl)benzenesulfonamide (**4**) was reacted with substituted isocyanates or isothiocyanates to obtain the corresponding ureas **5a**–**u** and thioureas **6a**–**y**, respectively. The structure of the new compounds was confirmed by analytical data and is consistent with reported studies [18].

### 2.2. Carbonic Anhydrase Inhibition

The *h*CA I, *h*CA II, *h*CA IX and *h*CA XII inhibitory activity of sulfonamide derivatives **5a**–**u** (Table 1) and **6a**–**y** (Table 2) was assayed by a stopped flow CO_2_ hydrase assay using the standard inhibitor acetazolamide as positive control [23].

All urea derivatives **5a**–**u** demonstrated active on the tested *h*CA isoforms with Ki values ranging from low to high nanomolar concentration. The presence of a 2-chlorine on the aryl ring (compound **5b**) resulted in high activity on *h*CA-II, IX and XII while *h*CA-I was inhibited at higher concentrations. Moving the chlorine atom into 3-position (compound **5c**) *h*CA II activity was retained, while the activity on *h*CA XII and *h*CA I was worsened. Interestingly, the shift on the chlorine atom into 4-position (compound **5d**) reduced the activity against the four isoforms and *h*CA I particularly. The presence of a second chlorine atom (compound **5e**) caused a slight reduction in activity on *h*CA II and improvement in selectivity on *h*CA IX and *h*CA XII/*h*CA I when compared with the 2-Cl derivative **5b**.

The replacement of 2-chlorine with a methoxy group providing compound **5o**, improved the activity on *h*CA IX and *h*CA XII as well as the selectivity toward the target isoforms as compared to *h*CA I and *h*CA II. Similar results were obtained by the 4-methoxyphenylurea **5q**. Shifting the methoxy group into 3-position (compound **5p**) retained the activity on *h*CA IX and *h*CA XII and increased about 2-fold the activity on *h*CA II. The introduction of a second methoxy group (compound **5r**) produced a further increase in *h*CA II activity as well as reduction in *h*CA IX and *h*CA XII/*h*CA I selectivity as compared to the 2-methoxy **5o** and 4-methoxy **5q** analogs.

The replacement of 2-chlorine with a fluorine atom (compound **5f**) maintained the good activity on *h*CA II, *h*CA IX and *h*CA XII increasing the *h*CA IX, *h*CA XII/*h*CA I selectivity. The shift of fluorine atom into 3-position (compound **5g**) produced reduction in activity on *h*CA IX while the Ki values on *h*CA II and *h*CA XII are similar. Shifting the fluorine into 4-position (compound **5h**) improved *h*CA IX inhibitory activity with high *h*CA IX/*h*CA I and *h*CA IX/*h*CA II selectivity. The introduction of a second fluorine atom (compound **5i**) slight improved the activity on all *h*CA tested isoforms as compared to 2-fluorine analog **5f**, while the selectivity on *h*CA IX and *h*CA XII was worsened.

The introduction at 2-position of a methyl group (compound **5j**) gave good activity on all CA isoforms. The shift of the methyl group into 3-position (compound **5k**) produced change in the selectivity, the tumor-associated isoforms *h*CA IX and *h*CA XII being the best inhibited. Shifting the methyl into 4-position (compound **5l**) produced a further increase in *h*CA IX selectivity. The introduction of a second methyl group (compound **5m**) produced an increase in *h*CA IX, *h*CA XII/*h*CA I selectivity as compared to the 2-methyl analog **5j**.

The unsubstituted thiourea **6a** showed high inhibitory activity on *h*CA XII as well as about 8-fold selectivity on *h*CA I and *h*CA II. The introduction of a nitro group into 4-position (compound **6b**) gave selectivity on *h*CA IX if compared to *h*CA I (about 22-fold) and at minor extent if compared to *h*CA II and *h*CA XII (about 4- and 3-fold, respectively).

The replacement of the nitro group with a trifluoromethyl one (compound **6c**) produced the best activity on *h*CA II in thiourea series, also displaying high activity on *h*CA IX and *h*CA XII.

The introduction of a 2-chlorine atom (compound **6d**) produced reversal of *h*CA IX and *h*CA XII inhibition potency as compared to the unsubstituted analog **6a** while the selectivity towards these isoforms/*h*CA I was preserved. The shift of the chlorine into 3-position (compound **6e**) did not affect activity as well as selectivity. The shift of the chlorine into 4-position (compound **6f**) produced reduction in *h*CA II activity as compared to the 2- and 3-chlorine analogs. Interestingly, thiourea **6f** showed improvement of inhibitory activity on all the tested isoforms as compared to the urea analog **5d**. The introduction of a second chlorine atom (compound **6g**) produced increase in *h*CA IX activity as compared to the 3- and 4-chlorine derivatives followed by a high *h*CA IX/*h*CA I, *h*CA II selectivity. The shift of the second chlorine in 2-position (compound **6h**) caused about a 10-fold increase in *h*CA XII activity as compared to the 3,4-dichloro analog. Moving the second chlorine in 6-position (compound **6i**) produced an increase in *h*CA XII inhibitory activity as compared to 2-chlorine analog as well as a high increase in *h*CA XII/*h*CA I and *h*CA II selectivity.

The replacement of the 2-chlorine with a methoxy group (compound **6j**) slightly improved the activity on all *h*CA isoforms attended by about 35-fold *h*CA IX, *h*CA XII/*h*CA I selectivity. Interestingly, the shift of the methoxy group into 4-position (compound **6l**) or in 3-position (compound **6k**) reduced *h*CA IX and *h*CA XII activity and selectivity. The presence of a 3,4,5-trimethoxy group (compound **6m**) produced reduction in activity on *h*CA II as compared to 4-methoxy and 3-methoxy analogs but also a better *h*CA XII selectivity if compared to the 4-methoxy analog.

The introduction of a fluorine atom at 2-position (compound **6n**) produced high activity on *h*CA II, *h*CA IX and *h*CA XII. On shifting the fluorine atom on 3-position (compound **6o**) the low nanomolar *h*CA IX and *h*CA XII activity was maintained, with about 100- and 20-fold selectivity as compared to *h*CA I and *h*CA II, respectively. The 2,6-difluoro derivative **6q** displayed the same activity profile of **6o** with a reduction in selectivity. The 4-fluorine derivative **6p** showed reduction in activity and selectivity as compared to all fluorine-substituted thiourea derivatives. Furthermore, fluorine-substituted thioureas showed better *h*CA XII inhibitory activity than the urea analogs.

The 2-methylsubstitued thiourea **6r** showed the best activity on *h*CA IX. The shift of methyl group into 3-position (compound **6s**) caused about 8-fold increase in *h*CA II and *h*CA XII while the activity on *h*CA IX is almost unchanged as compared to the 2-methyl analog. The shift on the methyl group into 4-position (compound **6t**) caused a decrease in activity on all isoforms as compared to 2- and 3-methyl analogs, but the selectivity of *h*CA IX and *h*CA XII versus *h*CA I was maintained. The introduction of a second methyl group (compound **6u**) produced the best *h*CA IX and *h*CA XII selective compound of the methyl-substituted thioureas.

### 2.3. Molecular Docking

Eight selected compounds, four ureas and four thioureas (**5g**, **5m**, **5o**, **5q**, **6l**, **6j**, **6o** and **6u**), were docked in the four CA isoforms analyzed in this work to explore their interactions with CA active sites. The docking poses (Figures S1 and S2) are in agreement with what was already described in our previous study [20]. As expected, in this case the leading interaction is also represented by the coordination between the negatively charged nitrogen and the Zinc ion with the sulfonamide group deeply fitted into the active site. Moreover, as seen in other papers, the hydrogen of the sulfonamide establishes an H-Bond with the T199 which helps stabilize the system.

Despite the greater flexibility of this series of compounds which is responsible for the high variability of the observed binding pose, is it possible to retrieve some interaction that characterized the binding poses on the different isoforms. Concerning *h*CA II, the interactions with ASN62 and/or ASN67 are quite frequent as already observed. GLN92 is the recurrent interaction with *h*CA IX, especially for the thioureas series. Last but not least, the recurrent interaction with the *h*CA XII isoform is represented by the H-Bond between the ureidic/thioureidic -NH and SER132.

## 3. Materials and Methods

### 3.1. Chemistry

All commercially available solvents and reagents were used without further purification. ^1^H NMR spectra were recorded on an Inova 500 spectrometer (Varian, Palo Alto, CA, USA). ^1^H and ^13^C NMR spectra for compounds **5m**, **5o**, **6j** and **6u** were recorded on Bruker Avance III HD 600 spectrometer. The chemical shifts (δ) are reported in part per million downfield from tetramethylsilane (TMS), which was used as internal standard. The spectra were recorded in hexadeuteriodimethylsulphoxide (DMSO-*d*_6_). Infrared spectra were recorded on a Vector 22 spectrometer (Bruker, Bremen, Germany) in Nujol mulls. The main bands are given in cm^−1^. Positive-ion electrospray ionization (ESI) mass spectra were recorded on a double-focusing MAT 95 instrument (Finnigan, Waltham, MA, USA) with BE geometry. Melting points (mp) were determined with a SMP1 Melting Point apparatus (Stuart Scientific, Stone, UK) and are uncorrected. All products reported showed ^1^H NMR spectra in agreement with the assigned structures. The purity of the tested compounds was determined by combustion elemental analyses conducted by the Microanalytical Laboratory of the Chemistry Department of the University of Ferrara with a MT-5 CHN recorder elemental analyser (Yanagimoto, Kyoto, Japan) and the values found were within 0.4% of theoretical values. As previously reported, 4-(4-(Hydrazinecarbonyl)piperidine-1-carbonyl)benzenesulfonamide (**4**), ureas **5a**, **5c**, **5e**, **5h**, **5l**, **5m**, and **5r**–**u** were synthesized [18]. Briefly, the key intermediate **4** was prepared by this procedure

A 4-(Aminosulfonyl)benzoic acid (**1**) (4.2 g, 20 mmol), EDCI (3.9 g, 22 mmol) and HOBt (2.7 g, 20 mmol) were dissolved in anhydrous CH3CN (100 mL). The resulting mixture was stirred at rt for 30 min, then ethyl isonipecotate (2) (3.1 g, 20 mmol) was added. The mixture was stirred at rt for 12 h. The solvent was removed in vacuo and the residue was dissolved in ethyl acetate (30 mL) and washed sequentially with water (2 × 10 mL), saturated NaHCO_3_ aqueous solution (2 × 10 mL), 10% aqueous citric acid (2 × 10 mL) and brine (2 × 10 mL). The organic layer was dried over anhydrous sodium sulfate (Na_2_SO_4_), filtered and evaporated under reduced pressure. The residue was tritured with isopropyl ether (iPr_2_O) and the formed solid was filtered off, dried to obtain ethyl 1-(4-sulfamoylbenzoyl)piperidine-4-carboxylate (**3**) in 77% yield [18]. A mixture of crude ester **3** (4.9 g, 15 mmol) and hydrazine monohydrate (2.5 mL, 45 mmol) in EtOH was refluxed overnight. After cooling, the formed precipitate was filtered off, washed with water (3 × 10 mL), dried and used in the next step without further purification. Yield 78% [18].

#### 3.1.1. General Procedure for the Preparation of Benzenesulfonamidohydrazido Ureas (**5a**–**u**)

The appropriate isocyanate (1 mmol) was added to a solution of 4-(4-(hydrazinecarbonyl)piperidine-1-carbonyl)benzenesulfonamide **4** (0.33 g, 1 mmol) in dry EtOH (5 mL). The reaction mixture was refluxed overnight and then stirred until room temperature was reached. The formed precipitate was separated by suction, washed with Et_2_O (2 × 5 mL) and recrystalized from EtOH.

- *N-(2-Chlorophenyl)-2-(1-(4-sulfamoylbenzoyl)piperidine-4-carbonyl)hydrazinecarboxamide (***5b***).* Yield 67% m.p. 214–215 °C. ESIMS (*m*/*z*): 480, 482 (M+H)^+^.^1^H NMR (DMSO-*d*_6_): δ 1.58 (m, 2H, CH_2_), 1.70, 1.94 (m, 2H, CH_2_), 2.53, 2.90 (m, 2H, CH_2_), 3.10, 3.51 (m, 2H, CH_2_), 4.45 (m, 1H, CH), 7.03 (m, 1H, Ar), 7.28 (m, 1H, Ar), 7.43 (m, 1H, Ar), 7.46 (s, 2H, NH_2_), 7.57 (d, *J* = 7.5 Hz, 2H, Ar), 7.87 (d, *J* = 8.5 Hz, 2H, Ar), 8.07 (m, 1H, Ar), 8.17 (s, 1H, NH), 8.74 (s, 1H, NH), 9.83 (s, 1H, NH). IR (Nujol) 3334, 1608 cm^−1^. Elemental analysis: calculated for C_20_H_22_ClN_5_O_5_S (479.94) %C 50.05, %H 4.62, %N 14.59, found %C 50.09, %H 4.60 %N 14.64.
- *N-(4-Chlorophenyl)-2-(1-(4-sulfamoylbenzoyl)piperidine-4-carbonyl)hydrazinecarboxamide (***5d***).* Yield 72% m.p. 209–210 °C. ESIMS (*m*/*z*): 497 (M+H)^+^. ^1^H NMR (DMSO-*d*_6_): δ 1.55 (m, 2H, CH_2_), 1.74, 2.32 (m, 2H, CH_2_), 2.50, 2.88 (m, 2H, CH_2_), 3.06, 3.49 (m, 2H, CH_2_), 4.45 (m, 1H, CH), 7.29 (m, 1H, Ar), 7.44 (m, 4H, Ar and NH_2_), 7.57 (m, 3H, Ar), 7.87 (d, *J* = 8.5 Hz, 2H, Ar), 8.07 (s, 1H, NH), 9.01 (s, 1H, NH), 9.68 (s, 1H, NH). IR (Nujol) 3303, 3161, 1615 cm^−1^. Elemental analysis: calculated for C_20_H_22_ClN_5_O_5_S (496.00) %C 48.43, %H 4.47, %N 14.12, found %C 48.48, %H 4.46 %N 14.17.
- *N-(2-fluorophenyl)-2-(1-(4-sulfamoylbenzoyl)piperidine-4-carbonyl)hydrazinecarboxamide (***5f***).* Yield 82% m.p. 229–230 °C. ESIMS (*m*/*z*): 464 (M+H)^+^. ^1^H NMR (DMSO-*d*_6_): δ 1.57 (br s, 2H, CH_2_) 1.69, 1.84 (br s, 2H, CH_2_), 2.53, 2.90 (br s, 2H, CH_2_), 3.10, 3.51 (br s, 2H, CH_2_), 4.44 (br s, 1H, CH), 6.99 (m, 1H, Ar), 7.13 (m, 1H, Ar), 7.22 (m, 1H, Ar), 7.45 (s, 2H, NH_2_), 7.56 (d, *J* = 8.5 Hz, 2H, Ar), 7.88 (d, *J* = 8.5 Hz, 2H, Ar), 8.30 (m, 1H, Ar), 8.34 (s, 1H, NH), 8.51 (s, 1H, NH), 9.78 (s, 1H, NH). IR (Nujol) 3269, 1667, 1614 cm^−1^. Elemental analysis: calculated for C_20_H_22_FN_5_O_5_S (463.48) %C 51.83, %H 4.78, %N 15.11, found %C 51.77, %H 4.76, %N 15.15.
- *N-(3-fluorophenyl)-2-(1-(4-sulfamoylbenzoyl)piperidine-4-carbonyl)hydrazinecarboxamide (***5g***).* Yield 40% m.p. 214–215 °C. ESIMS (*m*/*z*): 464 (M+H)^+^. ^1^H NMR (DMSO-*d*_6_): δ 1.57 (br s, 2H, CH_2_) 1.71, 1.86 (br s, 2H, CH_2_), 2.54, 2.89 (br s, 2H, CH_2_), 3.08, 3.44 (br s, 2H, CH_2_), 4.45 (br s, 1H, CH), 6.76 (m, 1H, Ar), 7.15 (m, 1H, Ar), 7.27 (m, 1H, Ar), 7.45 (s, 3H, Ar and NH_2_), 7.57 (d, *J* = 8.5 Hz, 2H, Ar), 7.88 (d, *J* = 8.5 Hz, 2H, Ar), 8.13 (s, 1H, NH), 8.96 (s, 1H, NH), 9.70 (s, 1H, NH). IR (Nujol) 3355, 3264, 1673, 1615 cm^−1^. Elemental analysis: calculated for C_20_H_22_FN_5_O_5_S (463.48) %C 51.83, %H 4.78, %N 15.11, found %C 51.88, %H 4.77, %N 15.07.
- *N-(2,6-difluorophenyl)-2-(1-(4-sulfamoylbenzoyl)piperidine-4-carbonyl)hydrazinecarboxamide (***5i***).* Yield 34% m.p. 194–195 °C. ESIMS (*m/z*): 482 (M+H)^+^. ^1^H NMR (DMSO-*d*_6_): δ 1.56 (br s, 2H, CH_2_) 1.70, 1.85 (br s, 2H, CH_2_), 2.47, 2.87 (br s, 2H, CH_2_), 3.08, 3.50 (br s, 2H, CH_2_), 4.44 (br s, 1H, CH), 7.11 (m, 2H, Ar), 7.28 (m, 1H, Ar), 7.45 (s, 2H, NH_2_), 7.57 (d, *J* = 8.5 Hz, 2H, Ar), 7.87 (d, *J* = 8.5 Hz, 2H, Ar), 8.22 (s, 1H, NH), 8.31 (s, 1H, NH), 9.74 (s, 1H, NH). IR (Nujol) 3321, 3220, 1645, 1613 cm^−1^. Elemental analysis: calculated for C_20_H_21_F_2_N_5_O_5_S (481,47) %C 49.89, %H 4.40, %N 14.55, found %C 49.83, %H 4.41, %N 14.59.
- *2-(1-(4-Sulfamoylbenzoyl)piperidine-4-carbonyl)-N-(o-tolyl)hydrazinecarboxamide (***5j***).* Yield 87% m.p. 238–240 °C. ESIMS (*m/z*): 460 (M+H)^+^. ^1^H NMR (DMSO-*d*_6_): δ 1.57 (br s, 2H, CH_2_), 1.60, 1.85 (br s, 2H, CH_2_), 2.25 (s, 3H, CH_3_), 2.53, 2.89 (br s, 2H, CH_2_), 3.10, 3.51 (br s, 2H, CH_2_), 4.45 (br s, 1H, CH), 6.76 (d, *J* = 7.5 Hz, 1H, Ar), 7.12 (m, 1H, Ar), 7.23 (m, 2H, Ar), 7.45 (s, 2H, NH_2_), 7.57 (d, *J* = 8.5 Hz, 2H, Ar), 7.88 (d, *J* = 8.5 Hz, 2H, Ar), 7.95 (s, 1H, NH), 8.61 (s, 1H, NH), 9.66 (s, 1H, NH). IR (Nujol) 3358, 3243, 1639, 1615 cm^−1^. Elemental analysis: calculated for C_21_H_25_N_5_O_5_S (459.52) %C 54.89, %H 5.48, %N 15.24, found %C 54.94, %H 5.46, %N 15.18.
- *2-(1-(4-Sulfamoylbenzoyl)piperidine-4-carbonyl)-N-(m-tolyl)hydrazinecarboxamide (***5k***).* Yield 91% m.p. 222–225 °C. ESIMS (*m/z*): 460 (M+H)^+^. ^1^H NMR (DMSO-*d*_6_): δ 1.58 (br s, 2H, CH_2_), 1.74, 1.86 (br s, 2H, CH_2_), 2.26 (s, 3H, CH_3_), 2.53, 2.90 (br s, 2H, CH_2_), 3.10, 3.51 (br s, 2H, CH_2_), 4.46 (br s, 1H, CH), 6.77 (d, *J* = 7.5 Hz, 1H, Ar), 7.13 (m, 1H, Ar), 7.23 (m, 2H, Ar), 7.46 (s, 2H, NH_2_), 7.58 (d, *J* = 8.5 Hz, 2H, Ar), 7.89 (d, *J* = 8.5 Hz, 2H, Ar), 8.63 (s, 1H, NH), 9.03 (s, 1H, NH), 9.68 (s, 1H, NH). IR (Nujol) 3288, 3054, 1639, 1615 cm^−1^. Elemental analysis: calculated for C_21_H_25_N_5_O_5_S (459.52) %C 54.89, %H 5.48, %N 15.24, found %C 54.94, %H 5.46, %N 15.18.
- *N-(2,6-Dimethylphenyl)-2-(1-(4-sulfamoylbenzoyl)piperidine-4-carbonyl)hydrazinecarboxanide (***5m***)*. Yield 85% m.p. 212–213 °C. ESIMS (*m/z*): 474 (M+H)^+^. ^1^H NMR (DMSO-*d*_6_): δ 1.57 (m, 2H, CH_2_), 1.71, 1.83 (m, 2H, CH_2_), 2.15 (s, 6H, 2CH_3_), 2.51, 2.87 (m, 2H, CH_2_), 3.08, 3.51 (m, 2H, CH_2_), 4.45 (m, 1H, CH), 7.03 (m, 3H Ar) 7.45 (s, 2H, NH_2_), 7.58 (d, *J* = 8.0 Hz, 2H, Ar), 7.63 (s, 1H, NH), 7.83 (s, 1H, NH), 7.88 (m, 3H, Ar), 9.68 (s, 1H, NH). ^13^C NMR (DMSO-*d*_6_): δ 173.8, 167.7, 156.3, 144.6, 139.4, 136.0, 135.3, 127.5 (2C), 127.2 (2C), 126.0, 125.8 (2C), 64.9, 46.5, 40.9, 28.3, 27.8, 18.1 (2C), 15.1. IR (Nujol) 3323, 3224, 1611 cm^−1^. Anal. Calcd for C_22_H_27_N_5_O_5_S (473.55) %C 55.80, %H 5.75, %N 14.79, found %C 55.85, %H 5.76, %N 14.75
- *N-(3,5-Dimethylphenyl)-2-(1-(4-sulfamoylbenzoyl)piperidine-4-carbonyl)hydrazinecarboxamide (***5n***).* Yield 90%. m.p. 216–217 °C. ESIMS (*m/z*): 474 (M+H)^+^. ^1^H NMR (DMSO-*d*_6_): δ 1.56 (m, 2H, CH_2_), 1.70, 1.84 (m, 2H, CH_2_), 2.19 (s, 6H, CH_3_), 2.53, 2.89 (m, 2H, CH_2_), 3.09, 3.50 (m, 2H, CH_2_), 4.45 (m, 1H, CH), 6.58 (s, 1H, Ar), 7.04 (s, 2H, Ar), 7.43 (s, 2H, NH_2_), 7.56 (d, *J* = 8.0 Hz, 2H, Ar), 7.87 (d, *J* = 8.5 Hz, 2H, Ar), 7.91 (s, 1H, NH), 8.51 (s, 1H, NH), 9.65 (s, 1H, NH). IR (Nujol) 3289, 3068, 1644, 1556 cm^−1^. Elemental analysis: calculated for C_22_H_27_N_5_O_5_S (473.55) %C 55.80, %H 5.75, %N 14.79, found %C 55.85, %H 5.76, %N 14.72.
- *(2-Methoxyphenyl)-2-(1-(4-sulfamoylbenzoyl)piperidine-4-carbonyl)hydrazinecarboxamide* (**5o***)*. Yield 84% m.p. 219–220 °C. ESIMS (*m/z*): 476 (M+H)^+^. ^1^H NMR (DMSO-*d*_6_): δ 1.60 (br s, 2H, CH_2_), 1.70, 1.86 (br s, 2H, CH_2_), 2.53-2.91 (br s, 2H, CH_2_), 3.11, 3.52 (br s, 2H, CH_2_), 3.84 (s, 3H, OCH_3_), 4.46 (br s, 1H, CH), 6.87 (m, 1H, Ar), 6.93 (m, 1H, Ar), 7.00 (m, 1H, Ar), 7.46 (s, 2H, NH_2_), 7.59 (d, *J* = 8.5 Hz, 2H, Ar), 7.90 (d, *J* = 8.5 Hz, 2H, Ar), 8.04 (d, *J* = 8.0 Hz, 1H, Ar), 8.09 (s, 1H, NH), 8.59 (s, 1H, NH), 9.78 (s, 1H, NH). ^13^C NMR (DMSO-*d*_6_): δ 173.8, 167.8, 155.1, 147.5, 144.7, 139.5, 128.4, 127.4 (2C), 125.9 (2C), 121.9, 120.6, 118.0, 110.7, 55.7, 46.2, 40.9, 28.5, 27.9, 18.6. IR (Nujol) 3298, 3100, 1663, 1606 cm^−1^. Elemental analysis: calculated for C_21_H_25_N_5_O_6_S (475,52) %C 53.04, %H 5.30, %N 14.73, found %C 53.09, %H 5.31, %N 14.69. *m*/*z* 476.
- *(3-Methoxyphenyl)-2-(1-(4-sulfamoylbenzoyl)piperidine-4-carbonyl)hydrazinecarboxamide (***5p***).* Yield 62% m.p. 218–220 °C. ESIMS (*m*/*z*): 476 (M+H)^+^. ^1^H NMR (DMSO-*d*_6_): δ 1.56 (br s, 2H, CH_2_), 1.70, 1.85 (br s, 2H, CH_2_), 2.54, 2.87 (br s, 2H, CH_2_), 3.09, 3.51 (br s, 2H, CH_2_), 3.71 (s, 3H, OCH_3_), 4.45 (br s, 1H, CH), 6.54 (m, 1H, Ar), 6.94 (m, 1H, Ar), 7.14 (m, 2H, Ar), 7.44 (s, 2H, NH_2_), 7.57 (d, *J* = 8.5 Hz, 2H, Ar), 7.88 (d, *J* = 8.5 Hz, 2H, Ar), 7.98 (s, 1H, NH), 8.70 (s, 1H, NH), 9.67 (s, 1H, NH). IR (Nujol) 3317, 1651, 1614 cm^−1^. Elemental analysis: calculated for C_21_H_25_N_5_O_6_S (475,52) %C 53.04, %H 5.30, %N 14.73, found %C 52.99, %H 5.31, %N 14.77.
- *(4-Methoxyphenyl)-2-(1-(4-sulfamoylbenzoyl)piperidine-4-carbonyl)hydrazinecarboxamide (***5q***).* Yield 78% m.p. 228–230 °C. ESIMS (*m*/*z*): 476 (M+H)^+^. ^1^H NMR (DMSO*-d_6_*): δ 1.58 (br s, 2H, CH_2_), 1.74, 1.86 (br s, 2H, CH_2_), 2.54, 2.86 (br s, 2H, CH_2_), 3.08, 3.51 (br s, 2H, CH_2_), 3.70 (s, 3H, OCH_3_), 4.45 (br s, 1H, CH), 6.54 (d, *J* = 9.5 Hz, 1H, Ar), 7.33 (d, *J* = 8.5 Hz, 1H, Ar), 7.46 (s, 2H, NH_2_), 7.58 (m, 3H, Ar), 7.88 (m, 3H, Ar), 8.53 (s, 1H, NH), 9.02 (s, 1H, NH), 9.65 (s, 1H, NH). IR (Nujol) 3315, 3216, 1680, 1617 cm^−1^. Elemental analysis: calculated for C_21_H_25_N_5_O_6_S (475,52) %C 53.04, %H 5.30, %N 14.73, found %C 52.98, %H 5.32, %N 14.76.

#### 3.1.2. General Procedure for the Preparation of N-aryl-4-Sulfamoylbenzoyl-piperidine-4-carbonyl-hydrazincarbothioamides (**6a**–**y**)

A mixture of 4-(4-(hydrazinecarbonyl)piperidine-1-carbonyl)benzenesulfonamide **4** (0.33 g, 1 mmol) and the appropriate isothiocyanate (1 mmol) in absolute EtOH (5 mL) was refluxed overnight. After cooling, the formed precipitate was filtered off, washed with Et_2_O (2 × 5 mL) and recrystalized from EtOH.

- *N-phenyl-2-(1-(4-sulfamoylbenzoyl)piperidine-4-carbonyl)hydrazine-1-carbothioamide (***6a***).* Yield 98% m.p. 210–211 °C. ESIMS (*m/z*): 462 (M+H)^+^. ^1^H NMR (DMSO-*d*_6_): δ 1.56 (m, 2H, CH_2_), 1.76, 1.92 (m, 2H, CH_2_), 2.54, 2.87 (m, 2H, CH_2_), 3.08, 3.51 (m, 2H, CH_2_), 4.44 (m, 1H, CH), 7.15 (m, 1H, Ar), 7.32 (m, 3H, Ar), 7.44 (s, 3H, Ar and NH_2_), 7.56 (d, *J* = 7.5 Hz, 2H, Ar), 7.87 (m, 3H, Ar and NH), 9.51 (s, 1H, NH), 9.92 (s, 1H, NH). IR (Nujol) 3334, 3242, 3050, 1687, 1613 cm^−1^. Elemental analysis: calculated for C_20_H_23_N_5_O_4_S_2_ (461.56) %C 52.05, %H 5.02, %N 15.17, found %C 52.11, %H 5.02, %N 15.14.
- *N-(4-Nitrophenyl)-2-(1-(4-sulfamoylbenzoyl)piperidine-4-carbonyl)hydrazinecarbothioamide (***6b***).* Yield 85% m.p. 236–237 °C. ESIMS (*m/z*): 507 (M+H)^+^. ^1^H NMR (DMSO-*d*_6_) δ 1.56 (m, 2H, CH_2_), 1.76, 1.91 (m, 2H, CH_2_), 2.56, 2.89 (m, 2H, CH_2_), 3.09, 3.52 (m, 2H, CH_2_), 4.45 (m, 1H, CH), 7.43 (s, 2H, NH_2_), 7.56 (d, *J* = 8.0 Hz, 2H, Ar), 7.87 (m, 4H, Ar), 8.19 (d, *J* = 9.0 Hz, 2H, Ar), 9.86 (s, 1H, NH), 9.93 (s, 1H, NH), 9.97 (s, 1H, NH). IR (Nujol) 3317, 3220, 3137, 1678, 1598 cm^−1^. Elemental analysis: calculated for C_20_H_22_N_6_O_6_S_2_ (506.46) %C 47.42, %H 4.38, %N 16.59, found %C 47.47, %H 4.39, %N 16.63.
- *4-(4-(4-(4-(Trifluoromethyl)phenyl)piperazine-1-carbonyl)piperidine-1-carbonyl)benzenesulfonamide (***6c***).* Yield 47% m.p. 201–203 °C. ESIMS (*m/z*): 530 (M+H)^+^. ^1^H NMR (DMSO-*d*_6_): δ 1.56 (m, 2H, CH_2_), 1.76, 1.91 (m, 2H, CH_2_), 2.54, 2.88 (m, 2H, CH_2_), 3.08, 3.51 (m, 2H, CH_2_), 4.45 (m, 1H, CH), 7.42 (s, 2H, NH_2_), 7.56 (d, *J* = 8.0 Hz, 2H, Ar), 7.66 (d, *J* = 8.5 Hz, 2H, Ar), 7.73 (d, *J* = 8.5 Hz, 2H, Ar), 7.86 (d, *J* = 8.5 Hz, 2H, Ar), 9.75 (s, 2H, NH), 9.96 (s, 1H, NH). IR (Nujol) 3300, 1686, 1619 cm^−1^. Elemental analysis: calculated for C_21_H_22_F_3_N_5_O_4_S_2_ (529.55) %C 47.63, %H 4.19, %N 13.23, found %C 47.58, %H 4.20, %N 13.27.
- *N-(2-Chlorophenyl)-2-(1-(4-sulfamoylbenzoyl)piperidine-4-carbonyl)hydrazinecarbothioamide (***6d***)*. Yield 98% m.p. 209–210 °C. ESIMS (*m/z*): 497 (M+H)^+^. ^1^H NMR (DMSO-*d*_6_): δ 1.57 (m, 2H, CH_2_), 1.77, 1.93 (m, 2H, CH_2_), 2.63, 2.88 (m, 2H, CH_2_), 3.08, 3.53 (m, 2H, CH_2_), 4.46 (m, 1H, CH), 7.27 (m, 1H, Ar), 7.34 (m, 1H, Ar), 7.46 (s, 2H, NH_2_), 7.49 (m, 2H, Ar), 7.57 (d, *J* = 8.0 Hz, 2H, Ar), 7.87 (d, *J* = 8.5 Hz, 2H, Ar), 9.34 (s, 1H, NH), 9.69 (s, 1H, NH), 10.01 (s, 1H, NH). IR (Nujol) 3362, 3254, 1682, 1619 cm^−1^. Elemental analysis: calculated for C_20_H_22_ClN_5_O_4_S_2_ (496.00) %C 48.43, %H 4.47, %N 14.12, found %C 48.38, %H 4.45, %N 14.17.
- *N-(3-Chlorophenyl)-2-(1-(4-sulfamoylbenzoyl)piperidine-4-carbonyl)hydrazinecarbothioamide (***6e***)*. Yield 61% m.p. 226–227 °C. ESIMS (*m/z*): 497 (M+H)^+^. ^1^H NMR (DMSO-*d*_6_): δ 1.52 (m, 2H, CH_2_), 1.72, 1.89 (m, 2H, CH_2_), 2.53, 2.85 (m, 2H, CH_2_), 3.05, 3.49 (m, 2H, CH_2_), 4.43 (m, 1H, CH), 7.19 (d, *J* = 7.0 Hz, 1H, Ar), 7.25 (m, 3H, Ar), 7.42 (s, 2H, NH_2_), 7.55 (d, *J* = 8.0 Hz, 2H, Ar), 7.86 (d, *J* = 8.5 Hz, 2H, Ar), 8.33 (s, 1H, NH), 9.24 (s, 1H, NH), 9.77 (s, 1H, NH). IR (Nujol) 3277, 3175, 3087, 1677, 1544 cm^−1^. Elemental analysis: calculated for C_20_H_22_ClN_5_O_4_S_2_ (496.00) %C 48.43, %H 4.47, %N 14.12, found %C 48.36, %H 4.48, %N 14.09.
- *N-(4-Chlorophenyl)-2-(1-(4-sulfamoylbenzoyl)piperidine-4-carbonyl)hydrazinecarbothioamide (***6f***)*. Following the general procedure, the title compound was prepared starting from 4-chlorophenylisothiocyanate. Yield 79% m.p. 222–223 °C. ESIMS (*m/z*): 497 (M+H)^+^. ^1^H NMR (DMSO-*d*_6_): δ 1.55 (s, 2H, CH_2_), 1.74, 1.91 (m, 2H, CH_2_), 2.63, 2.88 (m, 2H, CH_2_), 3.08, 3.51 (m, 2H, CH_2_), 4.46 (m, 1H, CH), 7.36 (d, *J* = 8.5 Hz, 2H, Ar), 7.42 (s, 2H, NH_2_), 7.46 (m, 2H, Ar), 7.56 (d, *J* = 7.5 Hz, 2H, Ar), 7.86 (d, *J* = 8.0 Hz, 2H, Ar), 9.59 (s, 2H, NH), 9.91 (s, 1H, NH). IR (Nujol) 3322, 3290, 3185, 1686, 1590 cm^−1^. Elemental analysis: calculated for C_20_H_22_ClN_5_O_4_S_2_ (496.00) %C 48.43, %H 4.47, %N 14.12, found %C 48.48, %H 4.48, %N 14.08. *m/z* 597.
- *N-(3,4-Dichlorophenyl)-2-(1-(4-sulfamoylbenzoyl)piperidine-4-carbonyl)hydrazinecarbothioamide (***6g***)*. Yield 97% m.p. 232–233 °C. ESIMS (*m/z*): 531 (M+H)^+^. ^1^H NMR (DMSO-*d*_6_): δ 1.55 (m, 2H, CH_2_), 1.76, 1.91 (m, 2H, CH_2_), 1.53, 2.88 (m, 2H, CH_2_), 3.08, 3.52 (m, 2H, CH_2_), 4.45 (m, 1H, CH), 7.43 (s, 2H, NH_2_), 7.47 (d, *J* = 8.0 Hz, 2H, Ar), 7.56 (d, *J* = 7.0 Hz, 2H, Ar), 7.82 (s, 1H, Ar), 7.86 (d, *J* = 8.5 Hz, 2H, Ar), 9.64 (s, 1H, NH), 9.76 (s, 1H, NH), 9.95 (s, 1H, NH). IR (Nujol) 3343, 3271, 3149, 1683, 1540 cm^−1^. Elemental analysis: calculated for C_20_H_21_Cl_2_N_5_O_4_S_2_ (530.45) %C 45.29, %H 3.99, %N 13.20, found %C 45.33, %H 3.97, %N 13.25.
- *N-(2,4-Dichlorophenyl)-2-(1-(4-sulfamoylbenzoyl)piperidine-4-carbonyl)hydrazinecarbothioamide (***6h***)*. Yield 81% m.p. 220–221 °C. ESIMS (*m/z*): 531 (M+H)^+^. ^1^H NMR (DMSO-*d*_6_): δ 1.55 (m, 2H, CH_2_), 1.74, 1.89 (m, 2H, CH_2_), 2.53, 2.87 (m, 2H, CH_2_), 3.07, 3.50 (m, 2H, CH_2_), 4.44 (m, 1H, CH), 7.39 (m, 2H, Ar), 7.42 (s, 2H, NH_2_), 7.54 (s, 1H, Ar), 7.55 (d, *J* = 8.0 Hz, 2H, Ar), 7.86 (d, *J* = 8.5 Hz, 2H, Ar), 9.35 (s, 1H, NH), 9.74 (s, 1H, NH), 9.99 (s, 1H, NH). IR (Nujol) 3243, 1681 cm^−1^. Elemental analysis: calculated for C_21_H_21_N_5_O_5_S_2_ (530.45) %C 45.29, %H 3.99, %N 13.20, found %C 45.23, %H 3.98, %N 13.23.
- *N-(2,6-Dichlorophenyl)-2-(1-(4-sulfamoylbenzoyl)piperidine-4-carbonyl)hydrazinecarbothioamide (***6i***)*. Yield 90% m.p. 179–180 °C. ESIMS (*m/z*): 531 (M+H)^+^. ^1^H NMR (DMSO-*d*_6_): δ 1.57 (m, 2H, CH_2_), 1.79, 1.95 (m, 2H, CH_2_), 2.53, 2.87 (m, 2H, CH_2_), 3.08, 3.53 (m, 2H, CH_2_), 4.46 (m, 1H, CH), 7.33 (m, 1H, Ar), 7.44 (s, 2H, NH_2_), 7.49 (m, 2H, Ar), 7.57 (d, *J* = 8.0 Hz, 2H, Ar), 7.87 (d, *J* = 8.5 Hz, 2H, Ar), 9.42 (s, 1H, NH), 9.71 (s, 1H, NH), 10.00 (s, 1H, NH). IR (Nujol) 3451, 3228, 1691, 1619 cm^−1^. Elemental analysis: calculated for C_21_H_21_N_5_O_5_S_2_ (530.45) %C 45.29, %H 3.99, %N 13.20, found %C 45.24, %H 4.02, %N 13.17.
- *N-(2-methoxyphenyl)-2-(1-(4-sulfamoylbenzoyl)piperidine-4-carbonyl)hydrazine-1-carbothioamide (***6j***)*. Yield 98% m.p. 214–215 °C. ESIMS (*m/z*): 492 (M+H)^+^. ^1^H NMR (DMSO-*d*_6_): δ 1.61 (m, 2H, CH_2_), 1.76, 1.93 (m, 2H, CH_2_), 2.59, 2.91 (m, 2H, CH_2_), 3.12, 3.54 (m, 2H, CH_2_), 3.80 (s, 3H, OCH_3_), 4.48 (m, 1H, CH), 6.93 (t, *J* = 8.27, 1H, Ar), 7.04 (d, *J* = 8.26, 1H, Ar), 7.15 (t, *J* = 8.50, 1H, Ar), 7.47 (s, 2H, NH_2_), 7.59 (d, *J* = 8.5 Hz, 2H, Ar), 7.90 (d, *J* = 8.5 Hz, 2H, Ar), 8.09 (m, 1H, Ar), 8.91 (s, 1H, NH), 9.65 (s, 1H, NH), 10.08 (s, 1H, NH). ^13^C NMR (DMSO-*d*_6_): δ 167.8, 151.3, 144.7, 139.4, 127.6, 127.3 (2C), 125.9 (2C), 124.6, 119.8 (2C), 111.3, 56.1, 55.8 (2C), 46.5, 40.9, 28.3, 27.8, 18.6. IR (Nujol) 3305, 3272, 3223, 1683, 1590 cm^−1^. Elemental analysis: calculated for C_21_H_25_N_5_O_5_S_2_ (491.58) %C 51.31, %H 5.13, %N 13.04, found %C 51.37, %H 5.11, %N 13.07.
- *N-(3-methoxyphenyl)-2-(1-(4-sulfamoylbenzoyl)piperidine-4-carbonyl)hydrazine-1-carbothioamide (***6k***)*. Yield 80% m.p. 214–215 °C. ESIMS (*m/z*): 492 (M+H)^+^. ^1^H NMR (DMSO-*d*_6_): δ 1.56 (m, 2H, CH_2_), 1.76, 1.92 (m, 2H, CH_2_), 2.52, 2.89 (m, 2H, CH_2_), 3.09, 3.52 (m, 2H, CH_2_), 3.74 (s, 2H, OCH_3_), 4.46 (m, 1H, CH), 6.73 (m, 1H, Ar), 7.00 (m, 1H, Ar), 7.23 (m, 2H, Ar), 7.45 (s, 2H, NH_2_), 7.57 (d, *J* = 8.5 Hz, 2H, Ar), 7.88 (d, *J* = 8.5 Hz, 2H, Ar), 9.53 (s, 2H, NH), 9.91 (s, 1H, NH). IR (Nujol) 3312, 3277, 3205, 1684, 1601, 1591 cm^−1^. Elemental analysis: calculated for C_21_H_25_N_5_O_5_S_2_ (491.58) %C 51.31, %H 5.13, %N 13.04, found %C 51.38, %H 5.11, %N 13.06.
- *N-(4-Methoxyphenyl)-2-(1-(4-sulfamoylbenzoyl)piperidine-4-carbonyl)hydrazinecarbothioamide (***6l***)*. Yield 84% m.p. >250 °C. ESIMS (*m/z*): 492 (M+H)^+^. ^1^H NMR (DMSO-*d*_6_) δ 1.54 (m, 2H, CH_2_), 1.75, 1.91 (m, 2H, CH_2_), 1.54, 1.87 (m, 2H, CH_2_), 3.07, 3.51 (m, 2H, CH_2_), 3.73 (s, 3H, OCH_3_), 4.44 (m, 1H, CH), 6.87 (d, *J* = 8.0 Hz, 2H, Ar), 7.24 (d, *J* = 7.5 Hz, 2H, Ar), 7.43 (s, 2H, NH_2_), 7.56 (d, *J* = 8.0 Hz, 2H, Ar), 7.86 (d, *J* = 8.5 Hz, 2H, Ar), 9.38 (s, 1H, NH), 9.43 (s, 1H, NH), 9.86 (s, 1H, NH). IR (Nujol) 3335, 323, 1680, 1543 cm^−1^. Elemental analysis: calculated for C_21_H_25_N_5_O_5_S_2_ (491.58) %C 51.31, %H 5.13, %N 13.05, found %C 51.36, %H 5.11, %N 13.01. *m/z* 492.
- *2-(1-(4-Sulfamoylbenzoyl)piperidine-4-carbonyl)-N-(3,4,5-trimethoxyphenyl)hydrazinecarbothioamide (***6m***)*. Yield 44% m.p. >250 °C. ESIMS (*m/z*): 552 (M+H)^+^. ^1^H NMR (DMSO-*d*_6_): δ 1.55 (m, 2H, CH_2_), 1.75, 1.90 (m, 2H, CH_2_), 2.53, 2.87 (m, 2H, CH_2_), 3.08, 3.52 (m, 2H, CH_2_), 3.63 (s, 3H, OCH_3_), 3.73 (s, 6H, OCH_3_), 4.44 (m, 1H, CH), 6.82 (s, 2H, Ar), 7.43 (s, 2H, NH_2_), 7.56 (d, *J* = 8 Hz, 2H, Ar), 7.86 (d, *J* = 8.5 Hz, 2H, Ar), 9.48 (s, 2H, NH), 9.88 (s, 1H, NH). IR (Nujol) 3533, 3284, 3168, 1692, 1565 cm^−1^. Elemental analysis: calculated for C_23_H_29_N_5_O_7_S_2_ (551.64) %C 50.08, %H 5.30, %N 12.70, found %C 50.01, %H 5.32, %N 12.66.
- *N-(2-Fluorophenyl)-2-(1-(4-sulfamoylbenzoyl)piperidine-4-carbonyl)hydrazinecarbothioamide (***6n***)*. Yield 98% m.p. 219–220 °C. ESIMS (*m/z*): 480 (M+H)^+^.^1^H NMR (DMSO-*d*_6_): δ 1.56 (m, 2H, CH_2_), 1.77, 1.93 (m, 2H, CH_2_), 2.63, 2.88 (m, 2H, CH_2_), 3.09, 3.52 (m, 2H, CH_2_), 4.46 (m, 1H, CH), 7.17 (m, 1H, Ar), 7.21 (m, 1H, Ar), 7.44 (s, 2H, NH_2_), 7.25 (m, 2H, Ar), 7.57 (d, *J* = 8.0 Hz, 2H, Ar), 7.88 (d, *J* = 8.5 Hz, 2H, Ar), 9.32 (s, 1H, NH), 9.70 (s, 1H, NH), 9.96 (s, 1H, NH). IR (Nujol) 3305, 3199, 1683, 1592 cm^−1^. Elemental analysis: calculated for C_20_H_22_FN_5_O_4_S_2_ (479.55) %C 50.09, %H 4.62, %N 14.60, found %C 50.17, %H 4.60, %N 14.63.
- *N-(3-Fluorophenyl)-2-(1-(4-sulfamoylbenzoyl)piperidine-4-carbonyl)hydrazinecarbothioamide (***6o***)*. Yield 46% m.p. 214–215 °C. ESIMS (*m/z*): 480 (M+H)^+^.^1^H NMR (DMSO-*d*_6_): δ 1.56 (m, 2H, CH_2_), 1.76, 1.92 (m, 2H, CH_2_), 2.55, 2.89 (m, 2H, CH_2_), 3.10, 3.53 (m, 2H, CH_2_), 4.46 (m, 1H, CH), 6.97 (s, 1H, Ar), 7.26 (m, 1H, Ar), 7.35 (m, 2H, Ar), 7.45 (s, 2H, NH_2_), 7.57 (d, *J* = 8.0 Hz, 2H, Ar), 7.88 (d, *J* = 8.5 Hz, 2H, Ar), 9.68 (s, 2H, NH), 9.96 (s, 1H, NH). IR (Nujol) 3333, 3266, 1686, 1620 cm^−1^. Elemental analysis: calculated for C_20_H_22_FN_5_O_4_S_2_ (479.55) %C 50.09, %H 4.62, %N 14.60, found %C 50.02, %H 4.64, %N 14.64. *m*/*z* 480.
- *N-(4-Fluorophenyl)-2-(1-(4-sulfamoylbenzoyl)piperidine-4-carbonyl)hydrazinecarbothioamide (***6p***).* Yield 85% m.p. 215–216 °C. ESIMS (*m/z*): 480 (M+H)^+^. ^1^H NMR (DMSO-*d*_6_) δ 1.55 (m, 2H, CH_2_), 1.76, 1.91 (m, 2H, CH_2_), 2.53, 2.87 (m, 2H, CH_2_), 3.08, 3.51 (m, 2H, CH_2_), 4.45 (m, 1H, CH), 7.14 (d, *J* = 8.5 Hz, 2H, Ar), 7.39 (m, 2H, Ar), 7.45 (s, 2H, NH_2_), 7.56 (d, *J* = 8.0 Hz, 2H, Ar), 7.87 (d, *J* = 8.0 Hz, 2H, Ar), 9.54 (s, 2H, NH), 9.90 (s, 1H, NH). IR (Nujol) 3320, 3175, 1685, 1563 cm^−1^. Elemental analysis: calculated for C_20_H_22_FN_5_O_4_S_2_ (479.55) %C 50.09, %H 4.62, %N 14.60, found %C 50.03, %H 4.60, %N 14.64.
- *N-(2,6-Difluorophenyl)-2-(1-(4-sulfamoylbenzoyl)piperidine-4-carbonyl)hydrazinecarbothioamide (***6q***)*. Yield 77% m.p. 242–243 °C. ESIMS (*m/z*): 498 (M+H)^+^. ^1^H NMR (DMSO-*d*_6_) δ 1.55 (m, 2H, CH_2_), 1.76, 1.92 (m, 2H, CH_2_), 2.55, 2.86 (m, 2H, CH_2_), 3.07, 3.38 (m, 2H, CH_2_), 4.45 (m, 1H, CH), 7.11 (d, *J* = 8.0 Hz, 2H, Ar), 7.33 (d, *J* = 7.0 Hz, 1H, Ar), 7.43 (s, 2H, NH_2_), 7.56 (d, *J* = 8.0 Hz, 2H, Ar), 7.86 (d, *J* = 8.5 Hz, 2H, Ar), 9.15 (s, 1H, NH), 9.83 (s, 1H, NH), 10.02 (s, 1H, NH). IR (Nujol) 3314, 3279, 3201, 1658, 1566 cm^−1^. Elemental analysis: calculated for C_20_H_21_F_2_N_5_O_4_S_2_ (497.54) %C 48.28, %H 4.25, %N 14.08, found %C 48.33, %H 4.26, %N 14.05.
- *2-(1-(4-sulfamoylbenzoyl)piperidine-4-carbonyl)-N-(o-tolyl)hydrazine-1-carbothioamide (***6r***).* Yield 98% m.p. 209–210 °C. ESIMS (*m/z*): 476 (M+H)^+^. ^1^H NMR (DMSO-*d*_6_): δ 1.56 (m, 2H, CH_2_), 1.77, 1.92 (m, 2H, CH_2_), 2.14 (s, 2H, CH_3_), 2.52, 2.87 (m, 2H, CH_2_), 3.08, 3.52 (m, 2H, CH_2_), 4.46 (m, 1H, CH), 7.16 (m, 4H, Ar), 7.45 (s, 2H, NH_2_), 7.57 (d, *J* = 8.5 Hz, 2H, Ar), 7.87 (d, *J* = 8.5 Hz, 2H, Ar), 9.29 (s, 1H, NH), 9.42 (s, 1H, NH), 9.92 (s, 1H, NH). IR (Nujol) 3300, 3192, 1685, 1591 cm^−1^. Elemental analysis: calculated for C_21_H_25_N_5_O_4_S_2_ (475.58) %C 53.04, %H 5.30, %N 14.73, found %C 52.98, %H 5.32, %N 14.77.
- *2-(1-(3-sulfamoylbenzoyl)piperidine-4-carbonyl)-N-(p-tolyl)hydrazine-1-carbothioamide (***6s***)*. Yield 72% m.p. 214–215 °C. ESIMS (*m/z*): 476 (M+H)^+^. ^1^H NMR (DMSO-*d*_6_): δ 1.56 (m, 2H, CH_2_), 1.77, 1.92 (m, 2H, CH_2_), 2.29 (s, 2H, CH_3_), 2.52, 2.87 (m, 2H, CH_2_), 3.08, 3.52 (m, 2H, CH_2_), 4.46 (m, 1H, CH), 6.99 (m, 1H, Ar), 7.19 (m, 3H Ar), 7.46 (s, 2H, NH_2_), 7.59 (d, *J* = 8.5 Hz, 2H, Ar), 7.87 (d, *J* = 8.5 Hz, 2H, Ar), 9.49 (s, 2H, NH), 9.91 (s, 1H, NH). IR (Nujol) 3316, 3291, 3197, 1684, 1591 cm^−1^. Elemental analysis: calculated for C_21_H_25_N_5_O_4_S_2_ (475.58) %C 53.04, %H 5.30, %N 14.73, found %C 52.98, %H 5.32, %N 14.76.
- *2-(1-(3-sulfamoylbenzoyl)piperidine-4-carbonyl)-N-(p-tolyl)hydrazine-1-carbothioamide (***6t***)*. Yield 80% m.p. 234–235 °C. ESIMS (*m/z*): 476 (M+H)^+^. ^1^H NMR (DMSO-*d*_6_): δ 1.56 (m, 2H, CH_2_), 1.76, 1.92 (m, 2H, CH_2_), 2.28 (s, 2H, CH_3_), 2.53, 2.88 (m, 2H, CH_2_), 3.09, 3.52 (m, 2H, CH_2_), 4.45 (m, 1H, CH), 7.13 (d, *J* = 7.5 Hz, 2H, Ar), 7.28 (d, *J* = 7.5 Hz, 2H, Ar), 7.45 (s, 2H, NH_2_), 7.57 (d, *J* = 8.5 Hz, 2H, Ar), 7.87 (d, *J* = 8.5 Hz, 2H, Ar), 9.44 (s, 2H, NH), 9.89 (s, 1H, NH). IR (Nujol) 3304, 3272, 3151, 1686, 1621 cm^−1^. Elemental analysis: calculated for C_21_H_25_N_5_O_4_S_2_ (475.58) %C 53.04, %H 5.30, %N 14.73, found %C 53.09, %H 5.28, %N 14.21.
- *N-(2,6-Dimethylphenyl)-2-(1-(4-sulfamoylbenzoyl)piperidine-4-carbonyl)hydrazinecarbothioamide (***6u***)*. Yield 64% m.p. 233–234 °C. ESIMS (*m/z*): 490 (M+H)^+^. ^1^H NMR (DMSO-*d*_6_): δ 1.57 (m, 2H, CH_2_), 1.71, 1.83 (m, 2H, CH_2_), 2.15 (s, 6H, 2CH_3_), 2.51, 2.87 (m, 2H, CH_2_), 3.08, 3.51 (m, 2H, CH_2_), 4.45 (m, 1H, CH), 7.03 (m, 3H Ar) 7.45 (s, 2H, NH_2_), 7.58 (d, *J* = 8.0 Hz, 2H, Ar), 7.83 (s, 1H, NH), 7.88 (m, 3H, Ar and NH), 9.68 (s, 1H, NH). ^13^C NMR (DMSO-*d*_6_): δ 173.22, 167.8, 167.7, 144.6, 139.5, 136.9, 136.6, 127.5, 127.3 (2C), 127.2 (2C), 126.8, 125.9 (2C), 46.5, 41.0, 40.0, 28.6, 28.0, 17.9 (2C) IR (Nujol) 3330, 3238, 1689 cm^−1^. Elemental analysis: calculated for C_22_H_27_N_5_O_4_S_2_ (489.61) %C 53.97, %H 5.56, %N 14.30, found %C 54.03, %H 5.55, %N 14.26.
- *N-Benzyl-2-(1-(4-sulfamoylbenzoyl)piperidine-4-carbonyl)hydrazinecarbothioamide (***6v***).* Following the general procedure, the title compound was prepared starting from benzylisothiocyanate. Yield 83% m.p. >250 °C. ESIMS (*m/z*): 476 (M+H)^+^. ^1^H NMR (DMSO-*d*_6_): δ 1.52 (m, 2H, CH_2_), 1.73, 1.88 (m, 2H, CH_2_), 2.45, 2.84 (m, 2H, CH_2_), 3.10, 3.49 (s, 2H, CH_2_), 4.42 (m, 1H, CH), 4.70 (s, 2H, CH_2_), 7.19 (m, 1H, Ar), 7.24 (m, 4H, Ar), 7.46 (s, 2H, NH_2_), 7.59 (d, *J* = 8.0 Hz, 2H, Ar), 7.88 (d, *J* = 8.5 Hz, 2H, Ar), 8.33 (s, 1H, NH), 9.24 (s, 1H, NH), 9.77 (s, 1H, NH). IR (Nujol) 3333, 3244, 1688, 1560 cm^−1^. Elemental analysis: calculated for C_21_H_25_N_5_O_4_S_2_ (475.58) %C 53.03, %H 5.30, %N 14.73, found %C 52.96, %H 5.29, %N 14.76.
- *N-(4-Methoxybenzyl)-2-(1-(4-sulfamoylbenzoyl)piperidine-4-carbonyl)hydrazinecarbothioamide (***6w***)*. Yield 64% m.p. 240–241 °C. ESIMS (*m/z*): 506 (M+H)^+^. ^1^H NMR (DMSO-*d*_6_): δ 1.51 (m, 2H, CH_2_), 1.72, 1.88 (m, 2H, CH_2_), 2.54, 2.85 (m, 2H, CH_2_), 3.04, 3.49 (m, 2H, CH_2_), 3.71 (s, 3H, OCH_3_), 4.42 (m, 1H, CH), 4.62 (s, 2H, CH_2_), 6.84 (d, *J* = 8.5 Hz, 2H, Ar), 7.19 (d, *J* = 8.5 Hz, 2H, Ar), 7.42 (s, 2H, NH_2_), 7.54 (d, *J* = 8.5 Hz, 2H, Ar), 7.85 (d, *J* = 8 Hz, 2H, Ar), 8.23 (s, 1H, NH), 9.18 (s, 1H, NH), 9.73 (s, 1H, NH). IR (Nujol) 3347, 3249, 3150, 1669, 1548 cm^−1^. Elemental analysis: calculated for C_22_H_27_N_5_O_5_S_2_ (505.61) %C 52.26, %H 5.38, %N 13.85, found %C 52.31, %H 5.36, %N 13.82.
- *N-(1-Phenylethyl)-2-(1-(4-sulfamoylbenzoyl)piperidine-4-carbonyl)hydrazinecarbothioamide (***6x***).* Yield 49% m.p. 196–197 °C. ESIMS (*m/z*): 490 (M+H)^+^. ^1^H NMR (DMSO-*d*_6_): δ 1.41 (d, *J* = 7.0, 3H, CH_3_), 1.53 (m, 2H, CH_2_), 1.72, 1.88 (m, 2H, CH_2_), 2.62, 2.86 (m, 2H, CH_2_), 3.06, 3.49 (m, 2H, CH_2_), 4.43 (m, 1H, CH), 5.57 (m, 1H, CH), 7.20 (m, 1H, Ar), 7.29 (m, 4H, Ar), 7.42 (s, 2H, NH_2_), 7.55 (d, *J* = 8.5 Hz, 2H, Ar), 7.86 (d, *J* = 8.5 Hz, 2H, Ar), 7.99 (s, 1H, NH), 9.14 (s, 1H, NH), 9.71 (s, 1H, NH). IR (Nujol) 3335, 3230, 3087, 1688, 1597 cm^−1^. Elemental analysis: calculated for C_22_H_27_N_5_O_4_S_2_ (489.61) %C 53.97, %H 5.56, %N 14.30, found %C 53.92, %H 5.58, %N 14.32.
- *N-Cyclohexyl-2-(1-(4-sulfamoylbenzoyl)piperidine-4-carbonyl)hydrazinecarbothioamide (***6y***)*. Yield 67% m.p. 183–184 °C. ESIMS (*m/z*): 468 (M+H)^+^. ^1^H NMR (DMSO-*d*_6_) δ 1.06 (m, 2H, CH_2_), 1.22 (m, 4H, CH_2_), 1.54 (m, 4H, CH_2_), 1.56 (m, 2H, CH_2_), 1.77, 1.86 (m, 2H, CH_2_), 2.53, 2.86 (m, 2H, CH_2_), 3.06, 3.50 (m, 2H, CH_2_), 4.03 (m, 1H, CH), 4.43 (m, 1H, CH), 7.36 (s, 1H, NH), 7.43 (s, 2H, NH_2_), 7.55 (d, *J* = 7.5 Hz, 2H, Ar), 7.86 (d, *J* = 8.0 Hz, 2H, Ar), 8.99 (s, 1H, NH), 9.64 (s, 1H, NH). IR (Nujol) 3324, 3177, 1672, 1555 cm^−1^. Elemental analysis: calculated for C_20_H_29_N_5_O_4_S_2_ (467.61) %C 51.37, %H 6.25, %N 14.98, found %C 51.42, %H 6.26, %N 14.94.

### 3.2. Carbonic Anhydrase Inhibition

An Applied Photophysics stopped-flow instrument has been used for assaying the CA catalyzed CO_2_ hydration activity using the Khalifah procedure [23]. The used indicator was phenol red (0.2 mM), the absorbance maximum was of 557 nm, and the buffer was 20 mM Hepes (pH 7.5), whereas 20 mM Na_2_SO_4_ were employed for maintaining the ionic strength constant. Initial rates of the CA-catalyzed CO_2_ hydration reaction were followed for a 10–100 s, working at CO_2_ concentrations from 1.7 to 17 mM. Six traces of the initial 5–10% of the reaction have been used for each inhibitor for the assessment of the initial velocity. Uncatalyzed rates were subtracted from the observed total rates. Standard acetazolamide and tested compounds stock solutions (0.1 mM) were prepared in 10% DMSO aqueous solution and were diluted up to 0.01 nM with the assay buffer. Inhibitor and enzyme solutions were preincubated together for 15 min, for assuring the formation of the E–I complex. The inhibition constants were obtained by non-linear least squares using the Cheng–Prusoff equation, as reported earlier [24,25,26] and represent the mean from at least three different determinations. All CA isoforms were recombinant ones obtained in-house as reported earlier. Their concentrations in the assay system were of 5.7–11.9 nM [21,27,28].

### 3.3. Molecular Docking

Molecular Docking simulations were carried out using Glide, the docking package of the Schrödinger suite [29]. The crystal structures of CAI (pdb 3w6h), CAII (pdb 3hs4), CAIX (pdb 3aia) and CAXII (pdb 1jd0) were retrieved from RCSB Protein Data Bank web server (http://www.rcsb.org/ (accessed on 1 March 2022)). All pdb files were pre-processed using the Protein Preparation workflow available on Maestro [30]. 3D ligands were prepared using an in-house python script developed using RDKit toolkit [31] and minimized using MMFF94 forcefield. The receptor grid was centred on the co-crystallized ligand, grid size was defined as 20 × 20 × 20 Å, and prepared following the standard protocol. Molecular docking was performed using the XP available method and the top scored pose was selected for the analysis.

## 4. Conclusions

In the present study, we described a small library of benzenesulfonamides as potential *h*CAIs, endowed with a piperidine ring, using hydrazinocarbonyl ureido/thioureido moiety as tail of inhibitors. Ureido derivatives **5a**–**u** and thioureido derivatives **6a**–**y** displayed different activities and a broad spectrum of selectivity against *h*CAI, *h*CAII, *h*CAIX and *h*CAXII. Overall, the presence of one or two halogens in different positions on the aromatic ring, strongly influence the activity and selectivity towards the CA isoforms, observing also significant differences moving from chlorine to fluorine and maintaining the same position in the ring. Regarding ureido derivatives, the 4-fluorophenyl derivative **5h** displayed the best activity against the cancer-related isoform *h*CAIX, with Ki 2.1 nM while the analogue 4-chlorophenyl **5d** resulted as about 18-fold less active against the same isoform. The introduction of methoxy group in *ortho* and *para*-position (compounds **5o**–**5q**) resulted in a high potency and selectivity against both *h*CAIX and *h*CAXII whereas the shifting of methoxy group in *meta*-position (compound **5p**) resulted in a decrease in selectivity. Compound **5u**, endowed with g-methoxybenzyl group, resulted as the best compound of the series against *h*CAXII, with Ki 6.4 nM and about 6-fold more selective if compared with *h*CAII, and *h*CAIX. Moving on thioureido derivatives, the 3,4-dichlorophenyl derivative **6g** inhibited *h*CAIX at low nanomolar levels, with a Ki 4.7 nM, also displaying good selectivity if compared with other isoforms. One of the most interesting compounds of the series resulted as the 3-fluorophenyl derivative **6o**, with excellent potency and selectivity against both *h*CAIX and *h*CAXII. A similar trend was also observed for compound **6u**, provided with 2,6-dimethylphenyl group. Finally, molecular docking analysis revealed the plausible key interactions that might explain the high activity and selectivity of these compounds.

## Figures and Tables

**Table 1:** Inhibition data of human CA isoforms CA I, II, IX and XII with sulfonamides **5a**–**u** reported here and the standard sulfonamide inhibitor acetazolamide (AAZ) by a stopped flow CO_2_ hydrase assay [23].

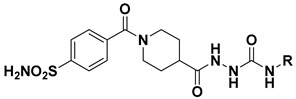					
Compound	R	Ki (nM)			
		*h*CA I	*h*CA II	*h*CA IX	*h*CA XII
**5a**	Phenyl	93.3	29.3	16.5	22.8
**5b**	2-Chlorophenyl	163.8	15.3	25.1	13.6
**5c**	3-Chlorophenyl	217.9	5.1	37.8	44.6
**5d**	4-Chlorophenyl	447.3	108.6	93.7	100.8
**5e**	2,6-Dichlorophenyl	259.4	68.8	28.9	16.4
**5f**	2-Fluorophenyl	562.1	24.6	22.5	8.4
**5g**	3-Fluorophenyl	277.2	17.7	89.7	19.4
**5h**	4-Fluorophenyl	60.6	12.1	2.1	24.0
**5i**	2,6-Difluorophenyl	184.1	8.6	10.1	12.7
**5j**	2-Methylphenyl	67.6	25.4	34.5	60.4
**5k**	3-Methylphenyl	94.5	114.3	8.2	15.6
**5l**	4-Methylphenyl	81.7	22.0	8.1	36.7
**5m**	2,6-Dimethylphenyl	363.6	62.1	25.1	51.2
**5n**	3,5-Dimethylphenyl	165.4	47.8	34.4	44.7
**5o**	2-Methoxyphenyl	451.5	185.0	8.6	19.6
**5p**	3-Methoxyphenyl	280.7	45.6	24.0	17.0
**5q**	4-Methoxyphenyl	487.2	99.7	8.9	9.1
**5r**	2,4-Dimethoxyphenyl	321.8	19.8	23.2	15.6
**5s**	Naphthyl	521.5	101.3	26.7	60.6
**5t**	Benzyl	77.8	48.2	40.0	20.1
**5u**	4-Methoxybenzyl	129.8	40.7	45.3	6.4
**AAZ**	/	250	12.5	25	5.7

**Table 2:** Inhibition data of human CA isoforms CA I, II, IX and XII with sulfonamides **6a**–**y** reported here and the standard sulfonamide inhibitor acetazolamide (AAZ) by a stopped flow CO_2_ hydrase assay [23].

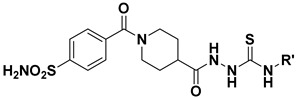					
Compound	R	Ki (nM)			
		*h*CAI	*h*CAII	*h*CAIX	*h*CAXII
**6a**	Phenyl	30.1	26.5	28.8	3.2
**6b**	4-Nitrophenyl	297.9	60.4	13.6	45.0
**6c**	4-(Trifluoromethyl)phenyl	52.0	3.2	7.2	4.5
**6d**	2-Chlorophenyl	172.9	20.6	8.2	27.1
**6e**	3-Chlorophenyl	322.8	16.6	18.0	9.7
**6f**	4-Chlorophenyl	130.9	41.6	26.1	17.7
**6g**	3,4-Dichlorophenyl	442.3	22.4	4.7	26.9
**6h**	2,4-Dichlorophenyl	228.6	53.4	18.0	2.6
**6i**	2,6-Dichlorophenyl	1337	21.5	24.2	10.0
**6j**	2-Methoxyphenyl	160.3	13.1	4.2	4.6
**6k**	3-Methoxyphenyl	145.6	11.2	25.1	29.9
**6l**	4-Methoxyphenyl	211.4	15.1	22.1	27.8
**6m**	3,4,5-Trimethoxyphenyl	428.1	36.8	16.6	27.3
**6n**	2-Fluorophenyl	430.5	15.2	6.9	4.9
**6o**	3-Fluorophenyl	590.1	101.7	5.6	4.2
**6p**	4-Fluorophenyl	79.6	23.0	20.1	15.9
**6q**	2,6-Difluorophenyl	65.4	28.2	5.9	5.6
**6r**	2-Methylphenyl	277.2	38.5	9.0	18.4
**6s**	3-Methylphenyl	56.5	4.3	9.3	2.6
**6t**	4-Methylphenyl	613.2	58.0	28.4	31.0
**6u**	2,6-Dimethylphenyl	307.9	89.6	4.7	9.5
**6v**	Benzyl	198.1	71.4	24.2	25.0
**6w**	4-Methoxybenzyl	178.3	29.3	31.7	29.9
**6x**	Phenylethyl	76.6	7.6	2.9	10.4
**6y**	Cyclohexyl	117.6	5.7	1.7	19.6
**AAZ**	/	250	12.5	25	5.7

## Data Availability

Not applicable.

## Supplementary Materials

The following supporting information can be downloaded at: https://www.mdpi.com/article/10.3390/molecules27175370/s1, Figures S1 and S2 best docking pose in CAI, CAII, CAIX and CAXII for **5g**, **5m**, **5o, 5q**, **6l**, **6j**, **6o**, **6u**; NMR spectra of the new ureas **5** and thioureas **6**, CAI, CAII, CAIX and CAXII inhibition curves for **5g**, **5m**, **5o, 5q**, **6l**, **6j**, **6o**, **6u**.

## Sample Availability

Samples of the compounds **5** and **6** are available from the authors.
