# Supplementary material for: Synthesis of Sulfonamides Incorporating Piperidinyl-Hydrazidoureido and Piperidinyl-Hydrazidothioureido Moieties and Their Carbonic Anhydrase I, II, IX and XII Inhibitory Activity
Source: Molecules. 2022 Aug 23;27(17):5370. doi: 10.3390/molecules27175370 (PMC9457746; doi:10.3390/molecules27175370)
Supplement: Supplementary file 1 [file molecules-27-05370-s001.zip › molecules-1874765-supplementary.pdf]

SUPPORTING INFORMATION

Synthesis of sulfonamides incorporating piperidinyl-hydrazidoureido and piperidinyl-hydrazidothioureido moieties and their carbonic anhydrase I, II, IX and XII inhibitory activity.

Davide Moi [1], Alessandro Deplano [2], Andrea Angeli [3], Gianfranco Balboni [1], Claudiu T. Supuran [3,*] and Valentina Onnis [1,*]

[1] Department of Life and Environmental Sciences, Unit of Pharmaceutical, Pharmacological and Nutraceutical Sciences, University of Cagliari, Monserrato University Campus, 09042 Monserrato, Italy; davide.moi2@gmail.com (D.M.); gbalboni@unica.it (G.B.)

[2] Pharmacelera, Torre R, 4a Planta, Despatx A05, Parc Cientific de Barcelona, Baldiri Reixac 8, 08028 Barcelona, Spain; deplano.a@gmail.com

[3] Polo Scientifico Neurofarba Department, Laboratorio di Chimica Bioinorganica, Università Degli Studi di Firenze, Room 188, Via della Lastruccia 3, Sesto Fiorentino, 50019 Florence, Italy; andrea.angeli@unifi.it

* Correspondence: claudiu.supuran@unifi.it (C.T.S.); vonnis@unica.it (V.O.)

Table of contents

	Pag.
Table S1-S2: best docking pose in CA I, CA II, CA IX and CA XII for **5g, 5m**, **5o**, **5q, 6l, 6j, 6o** and **6u.**	**S3-S4**
NMR Spectra of the ureas **5** and thioureas **6.**	**S5-S26**
CA-I, CA-II, CA-IX and CA-XII Inhibition curves for **5g, 5m, 5o, 5q, 6l, 6j, 6o** and **6u.**	**S27-S30**

Figure **S1**: Compounds **5o** and **6j** best docking pose in CA I, CA II, CA IX and CA XII.

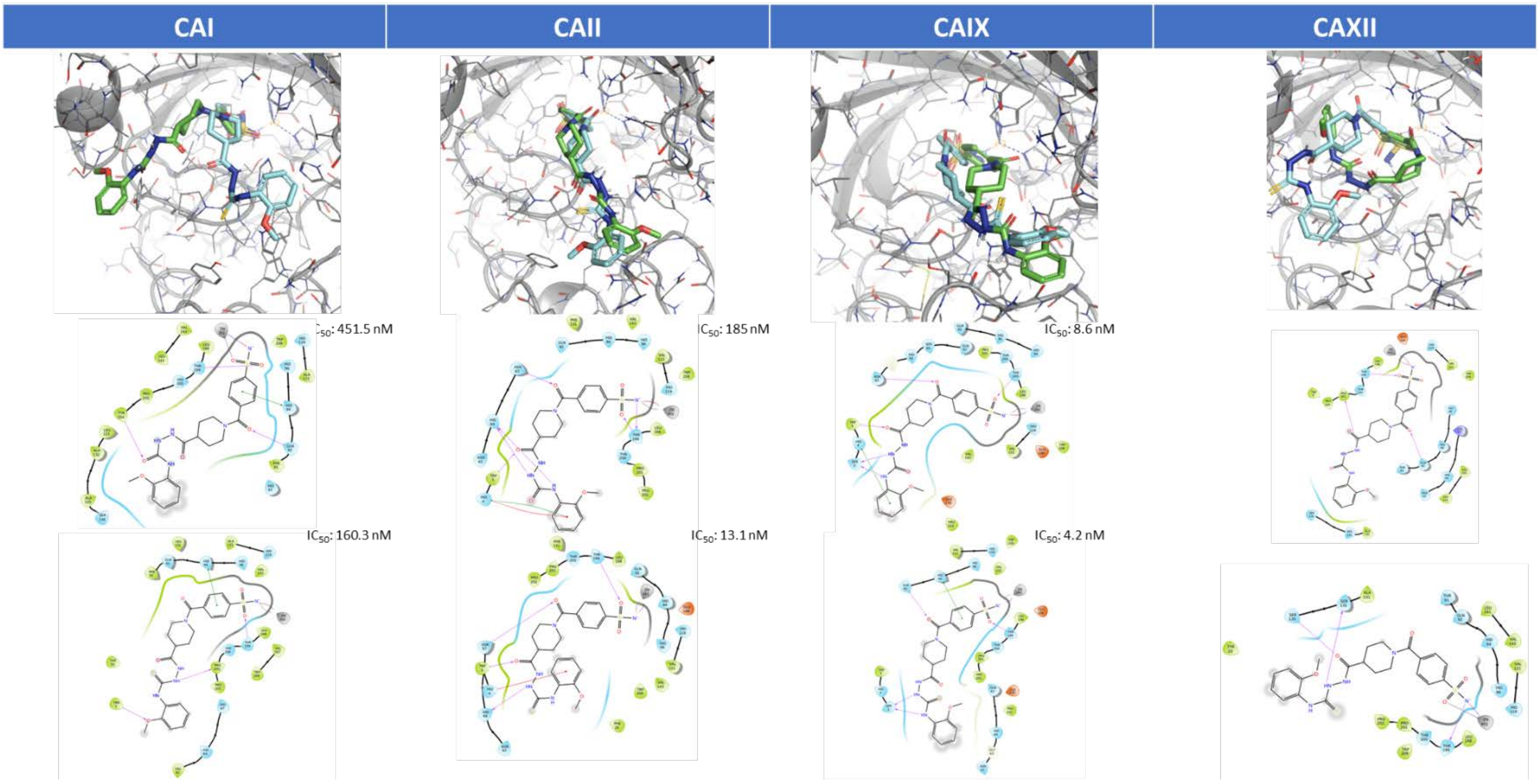

Figure **S2**: Compounds **5m** and **6u** best docking pose in CA I, CA II, CA IX and CA XII

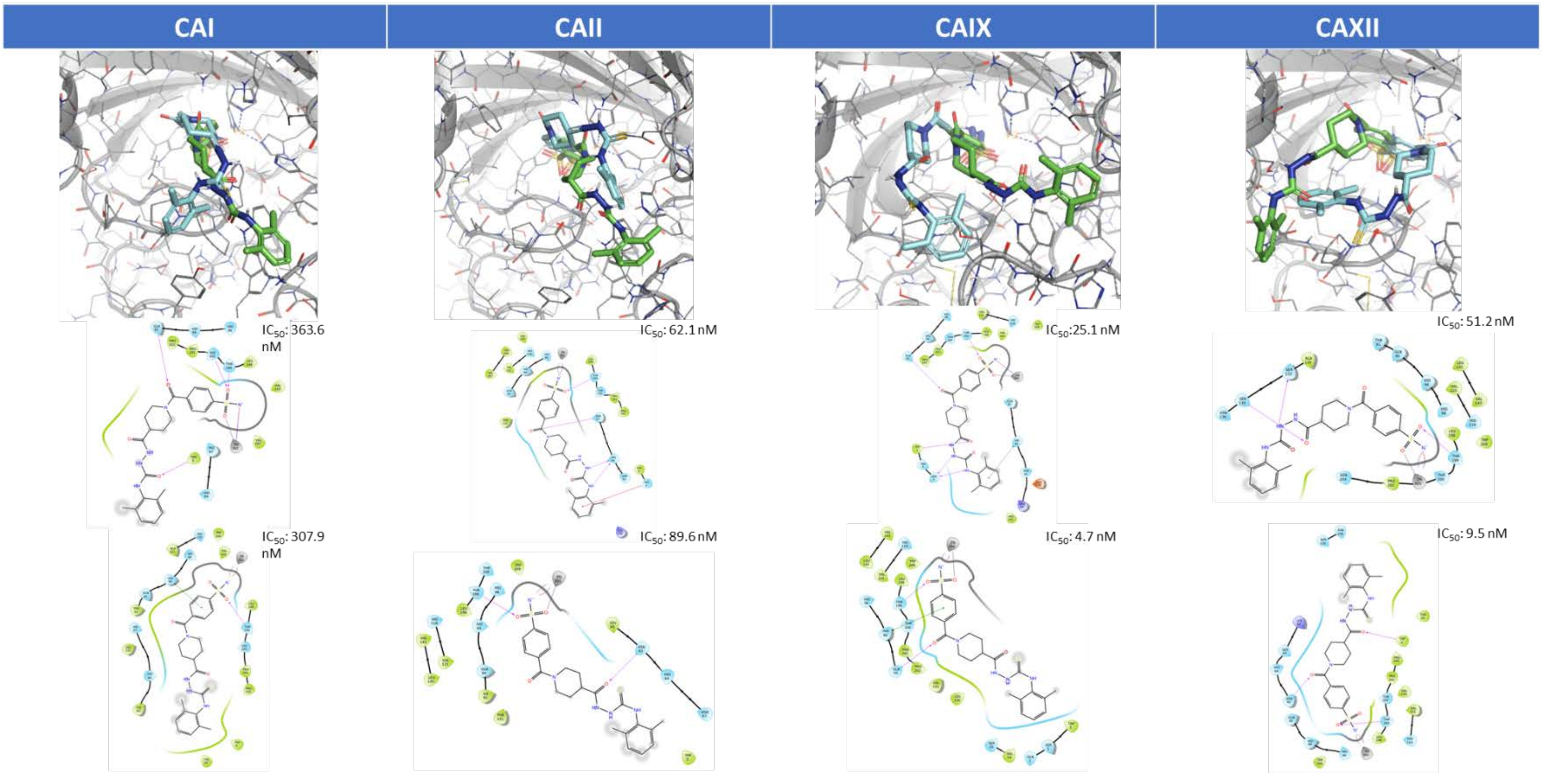

Figure **S3**: Compounds **5q** and **6l** best docking pose in CA I, CA II, CA IX and CA XII

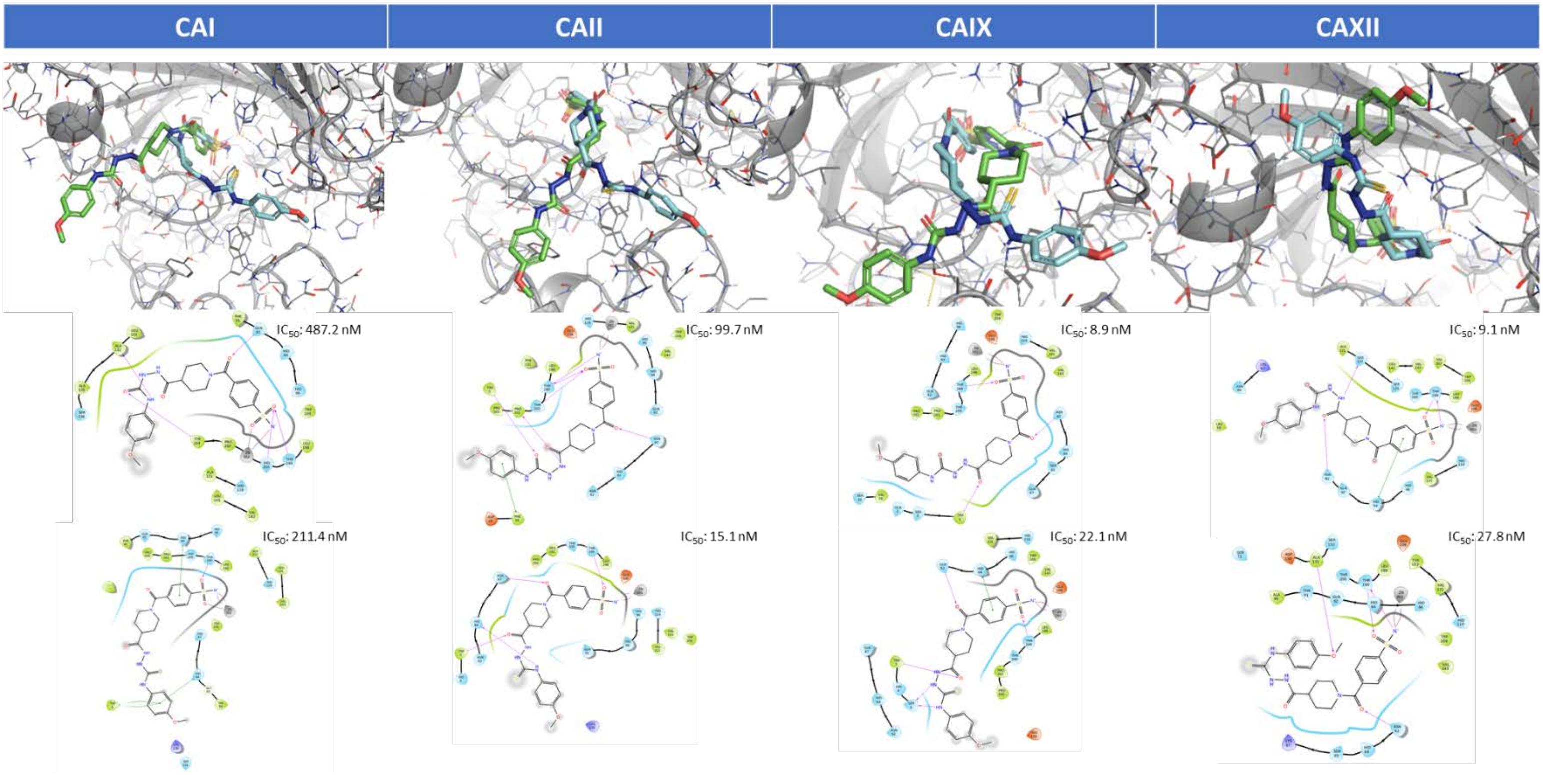

Figure **S4**: Compounds **5g** and **6o** best docking pose in CA I, CA II, CA IX and CA XII

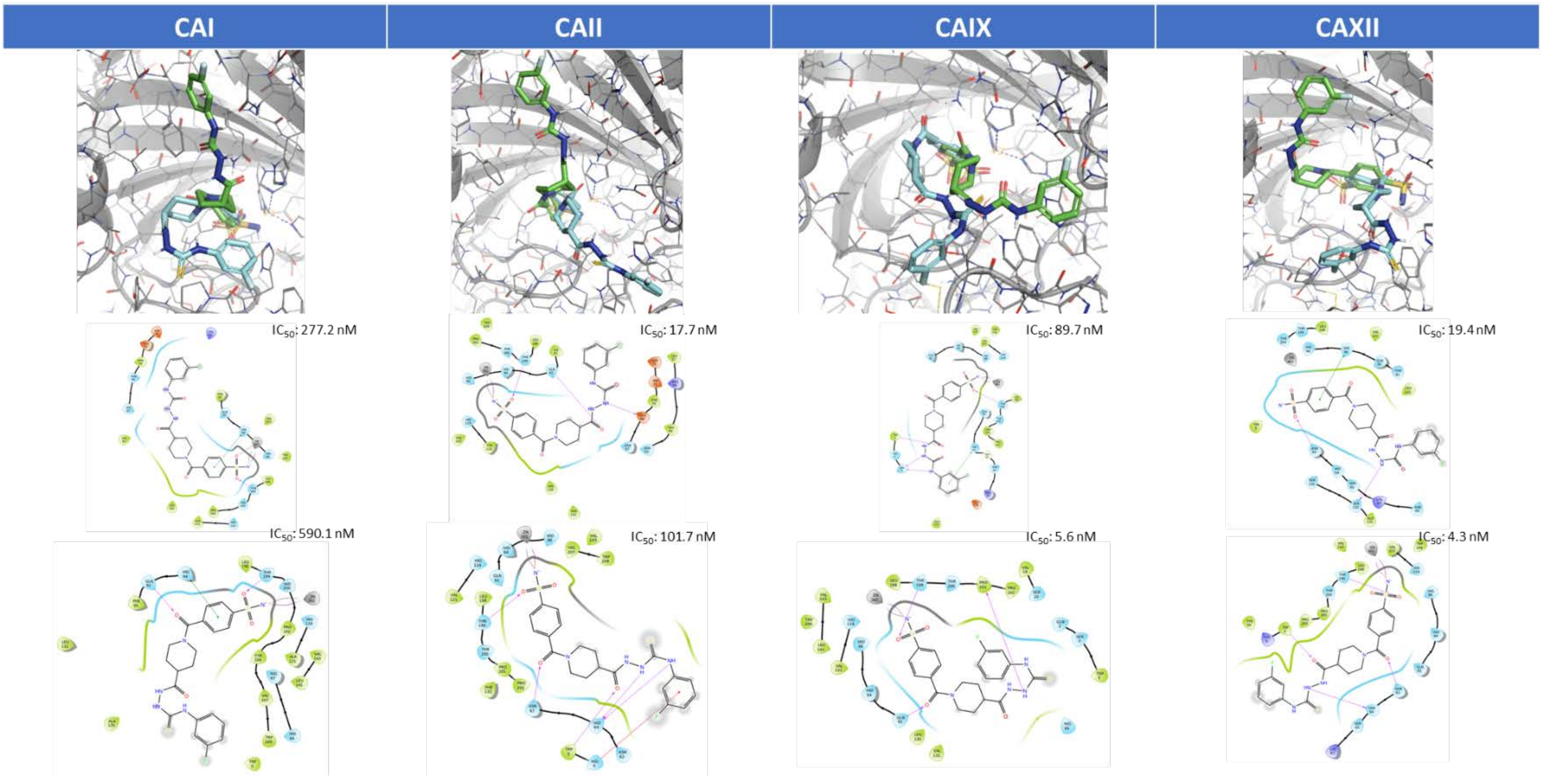

^{1}H, ^{13}C NMR Spectra for 5m, 5o, 6j, 6u

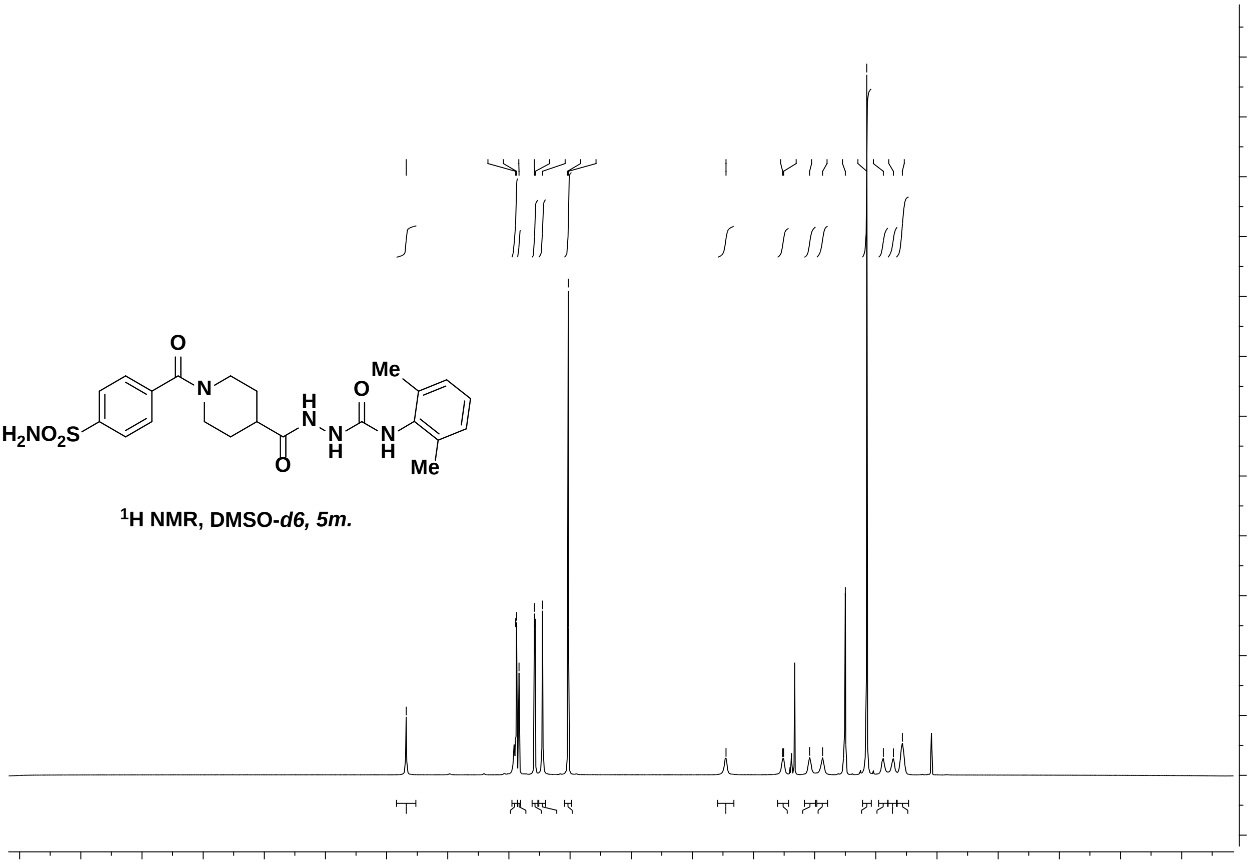

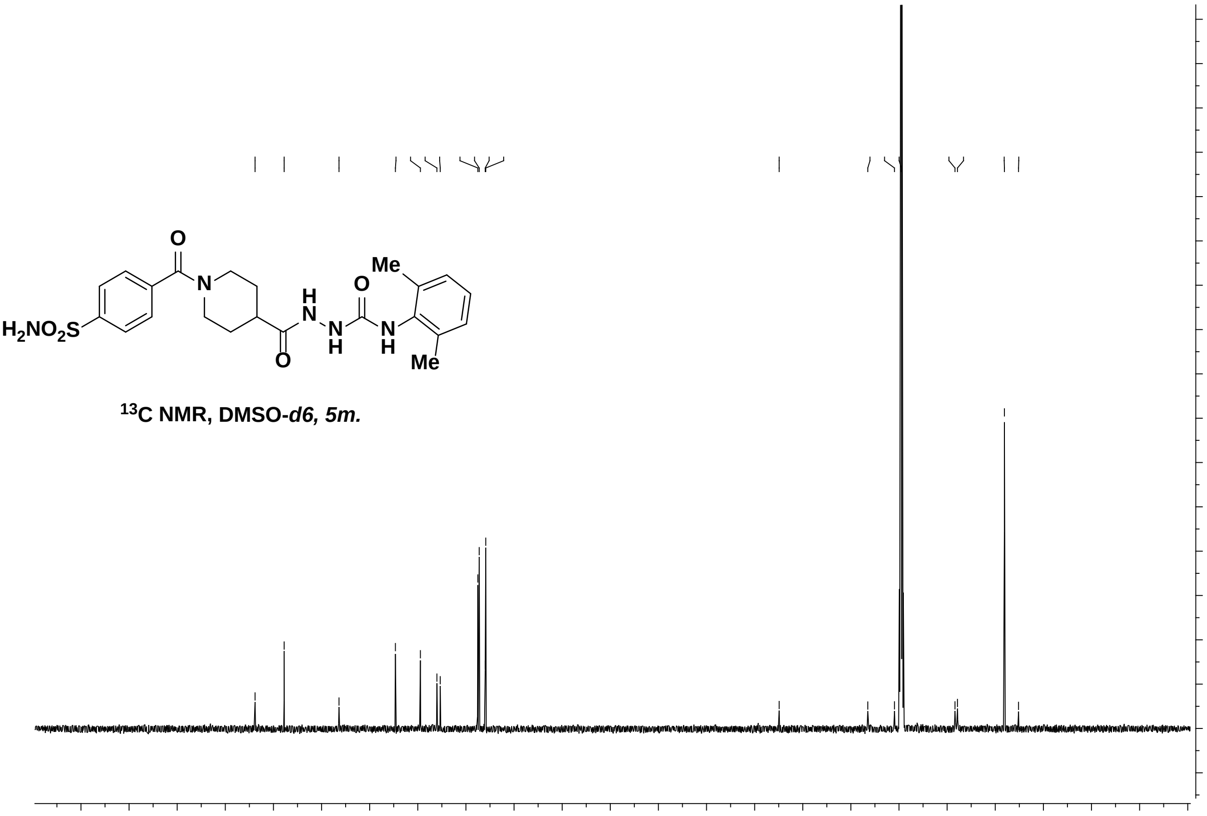

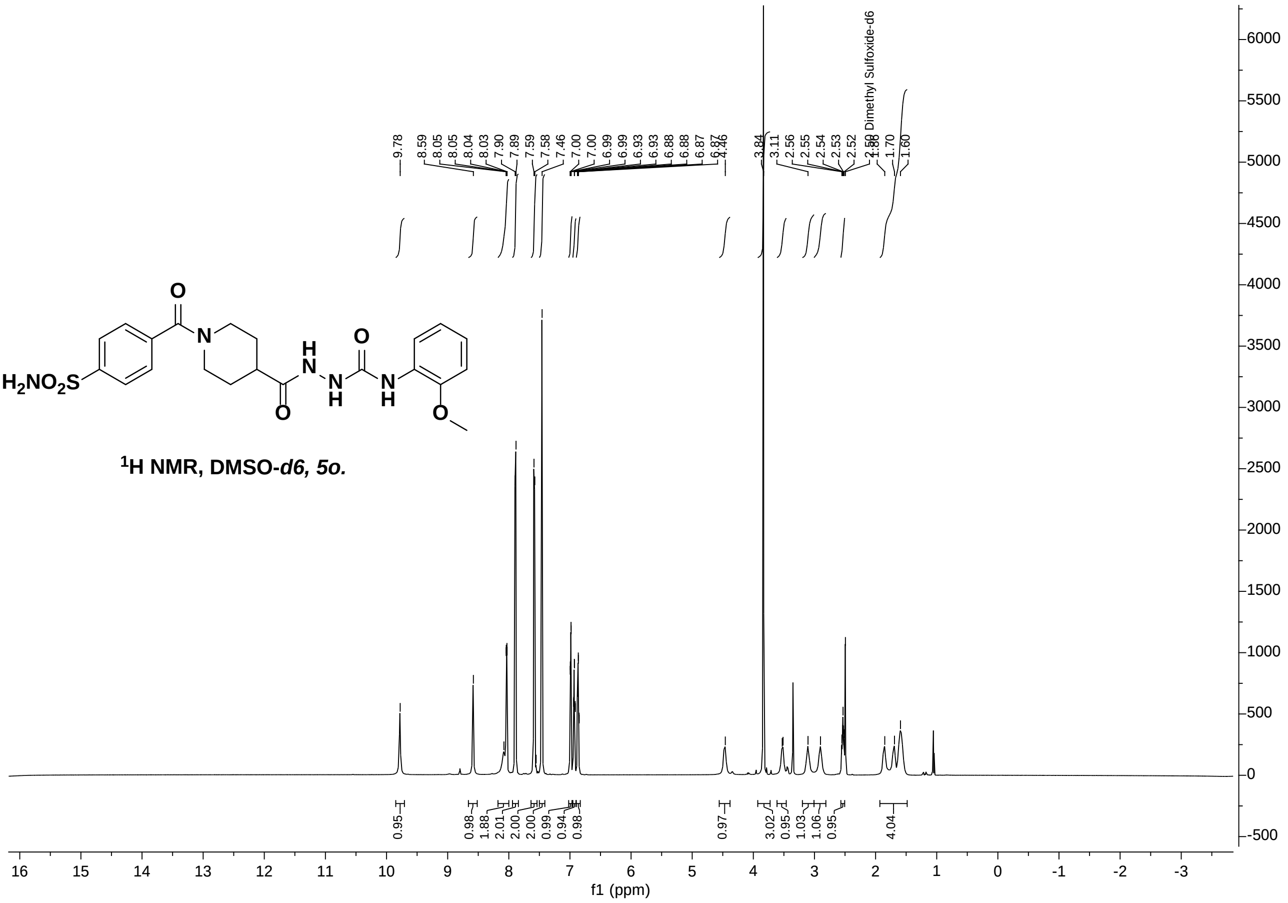
9.78
8.59
8.05
8.05
8.04
8.03
7.90
7.89
7.59
7.58
7.46
7.00
7.00
6.99
6.99
6.93
6.93
6.88
6.88
6.87
6.87
4.46
3.84
3.11
2.56
2.55
2.54
2.53
2.52
2.50 Dimethyl Sulfoxide-d6
1.88
1.70
1.60
0.95
0.98
1.88
2.01
2.00
2.00
0.99
0.94
0.98
0.97
3.02
0.95
1.03
1.06
0.95
4.04
H2NO2S
O
N
H
N
N
H
O
O
N
H
O
1H NMR, DMSO-d6, 5o.
f1 (ppm)

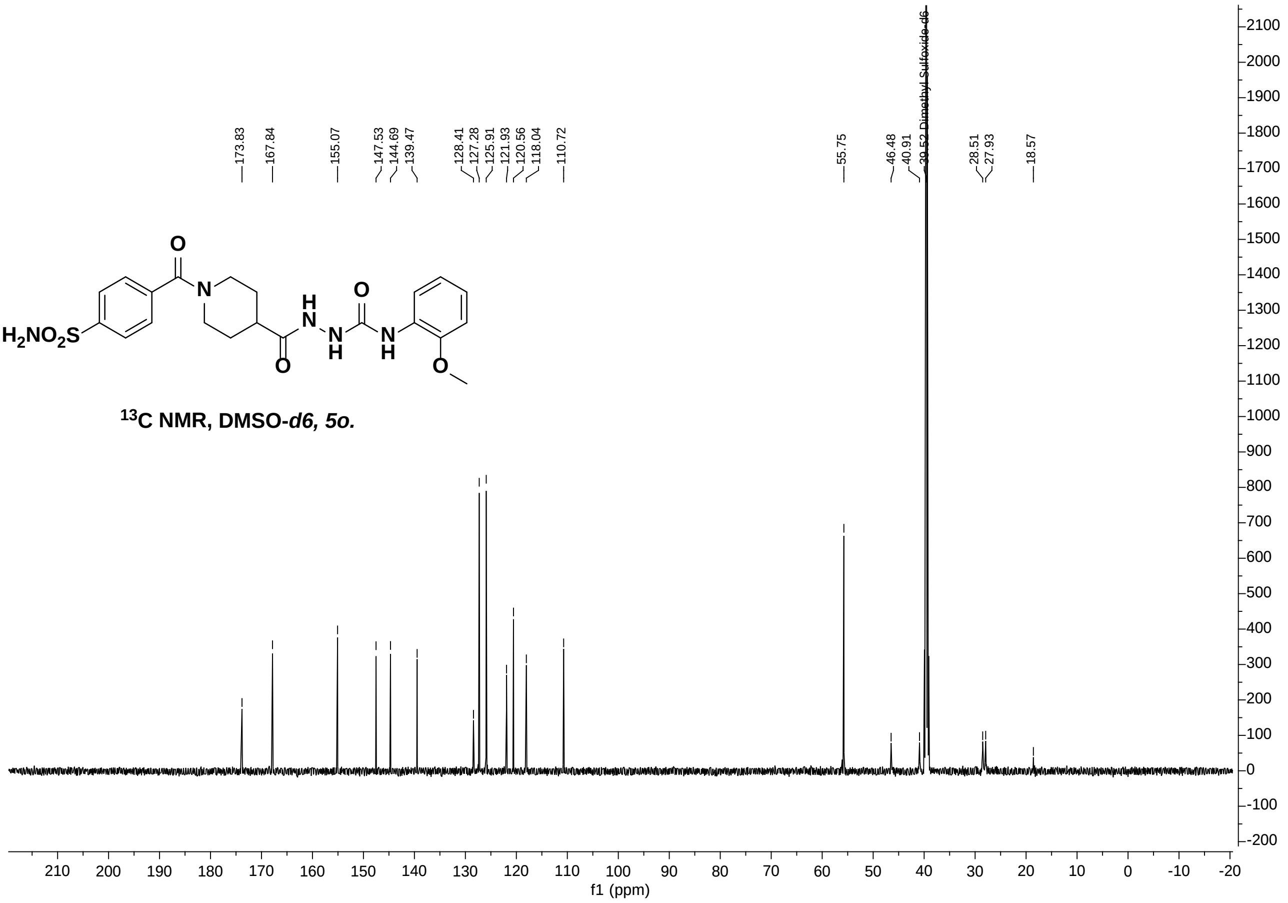
173.83
167.84
155.07
147.53
144.69
139.47
128.41
127.28
125.91
121.93
120.56
118.04
110.72
55.75
46.48
40.91
39.52 Dimethyl Sulfoxide-d6
28.51
27.93
18.57
H2NO2S
O
N
H
N
N
H
O
O
N
H
O
13C NMR, DMSO-d6, 5o.
f1 (ppm)

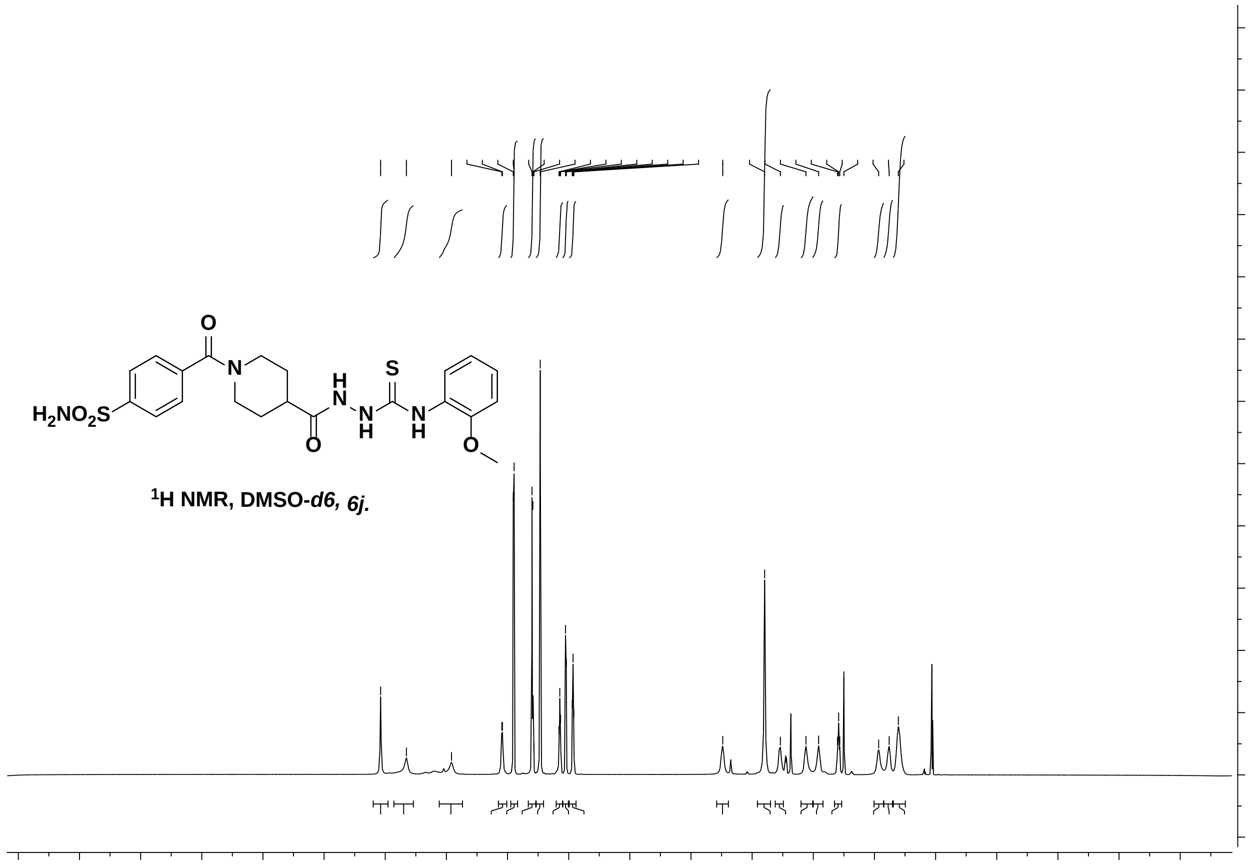

^{1}H NMR, DMSO-*d6, 6j.*

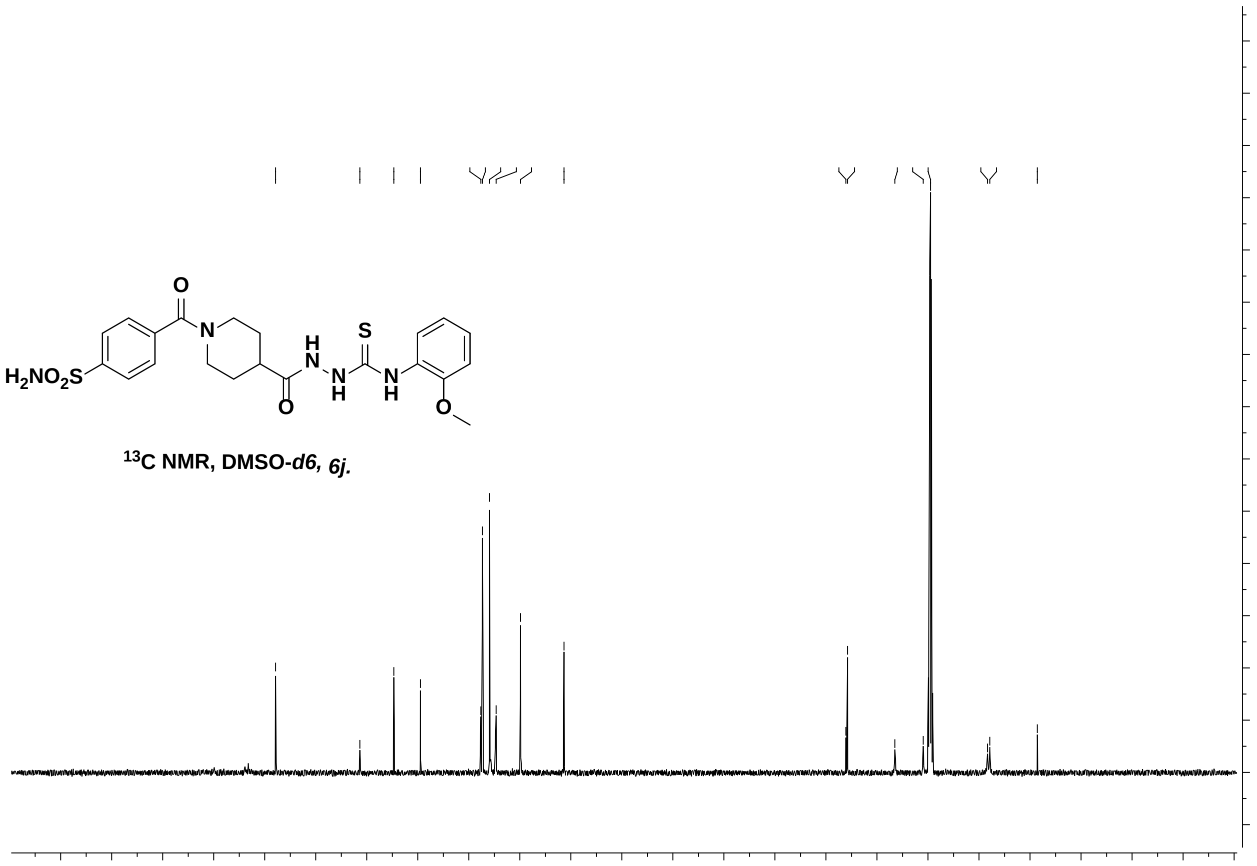

^{13}C NMR, DMSO-*d6, 6j.*

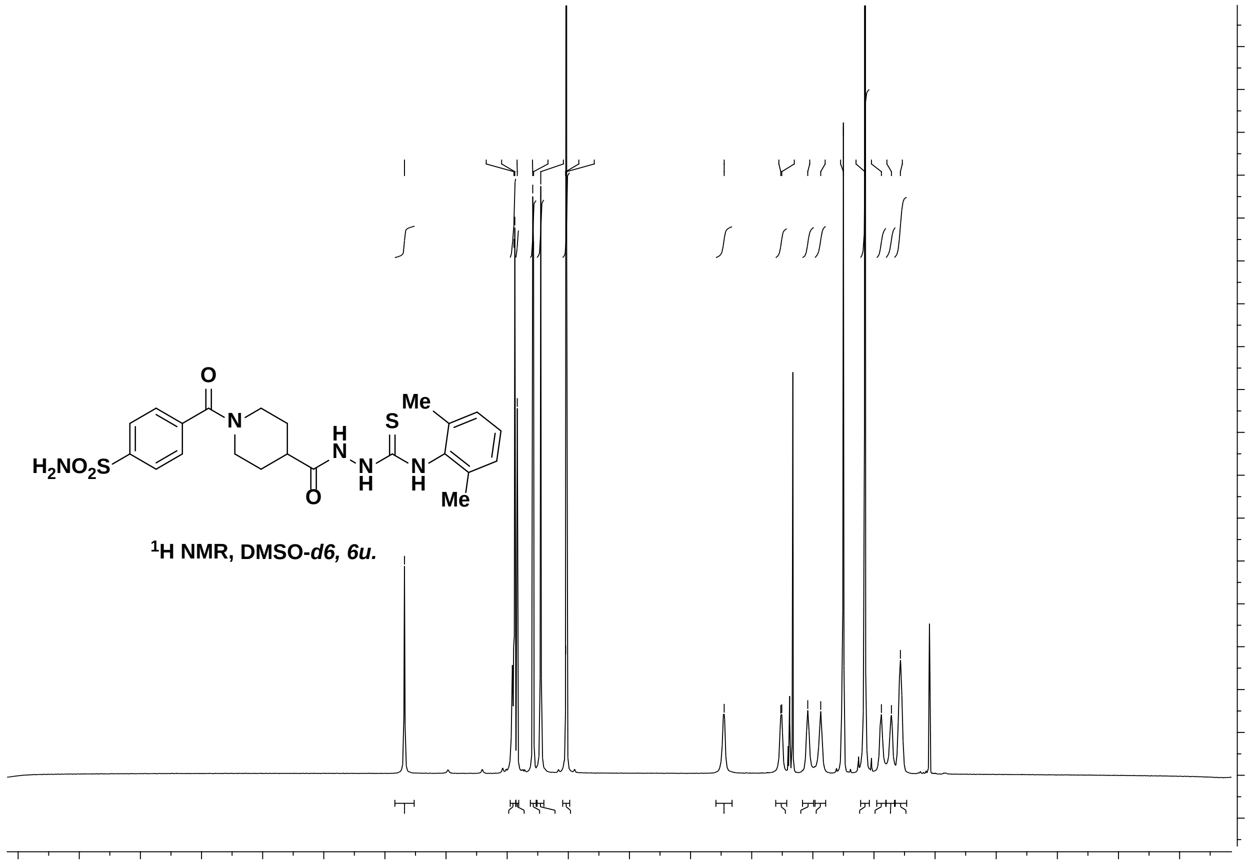
H2NO2S
O
N
H
N
N
H
S
N
H
Me
Me
O
1H NMR, DMSO-d6, 6u.

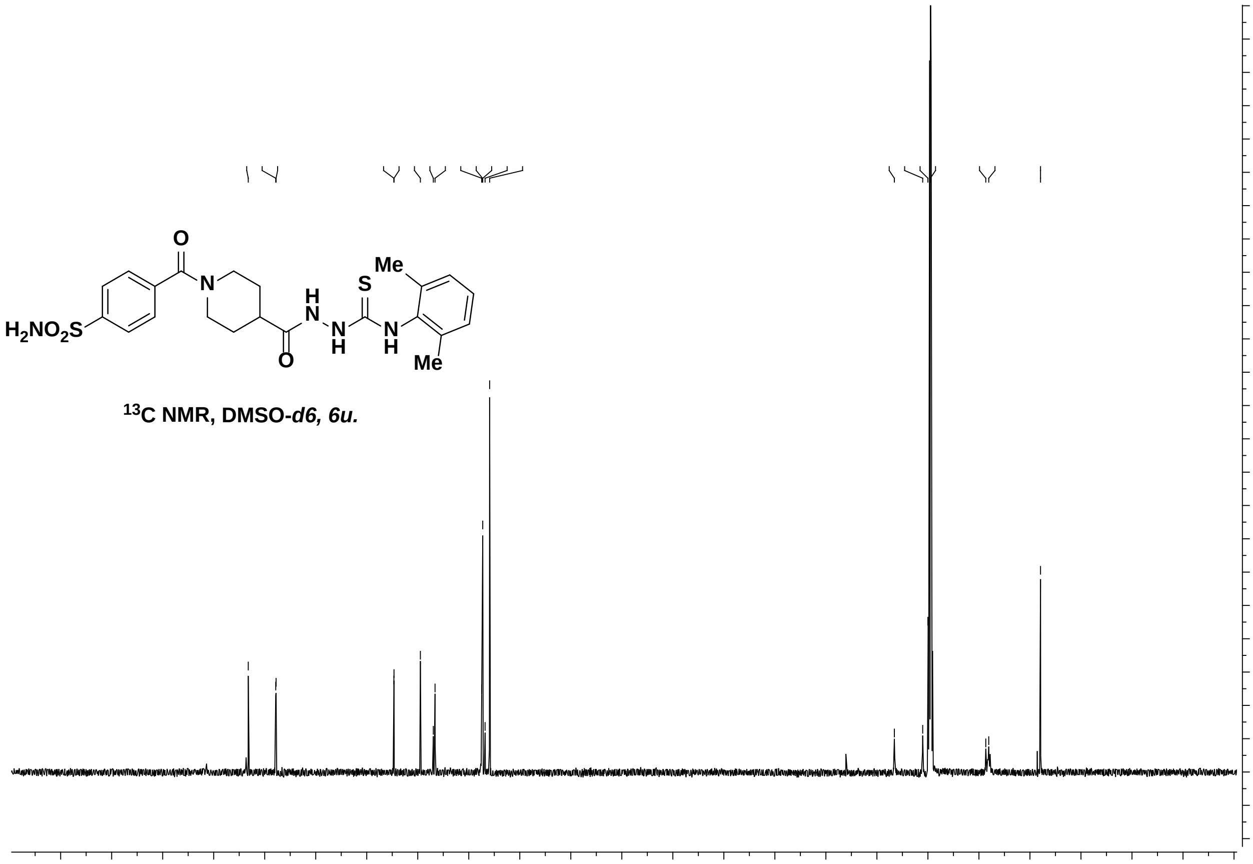
H2NO2S
O
N
H
N
N
H
S
N
H
Me
Me
O
13C NMR, DMSO-d6, 6u.

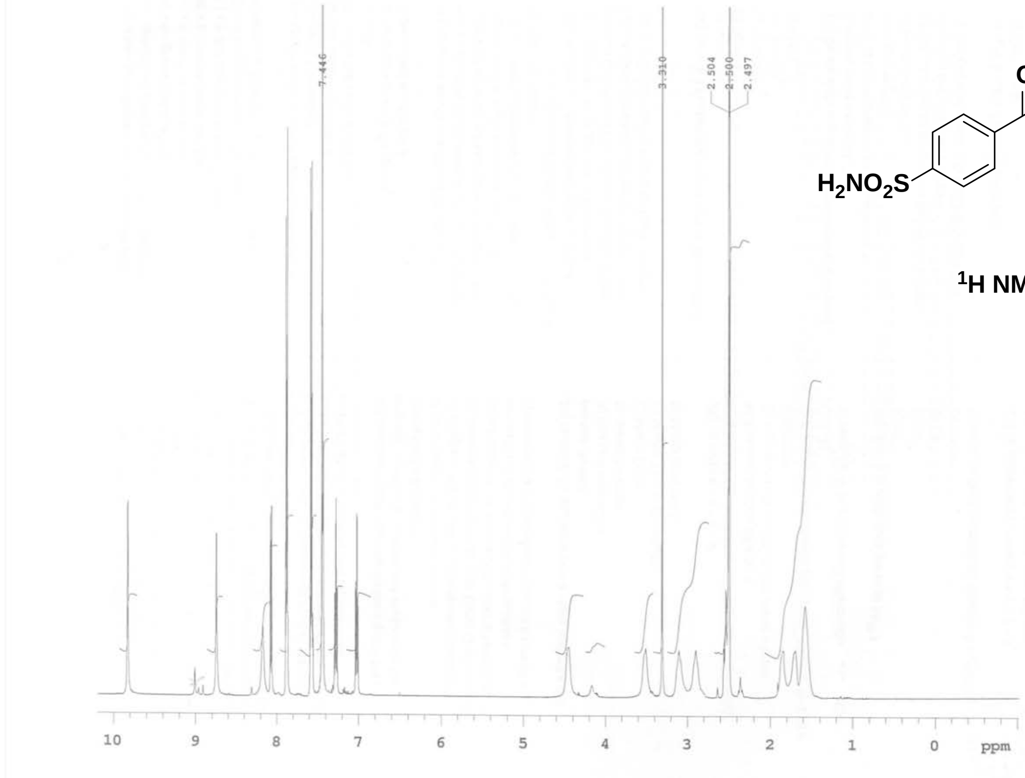

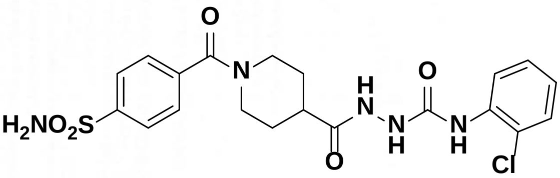

^{1}H NMR, DMSO-*d6, 5b.*

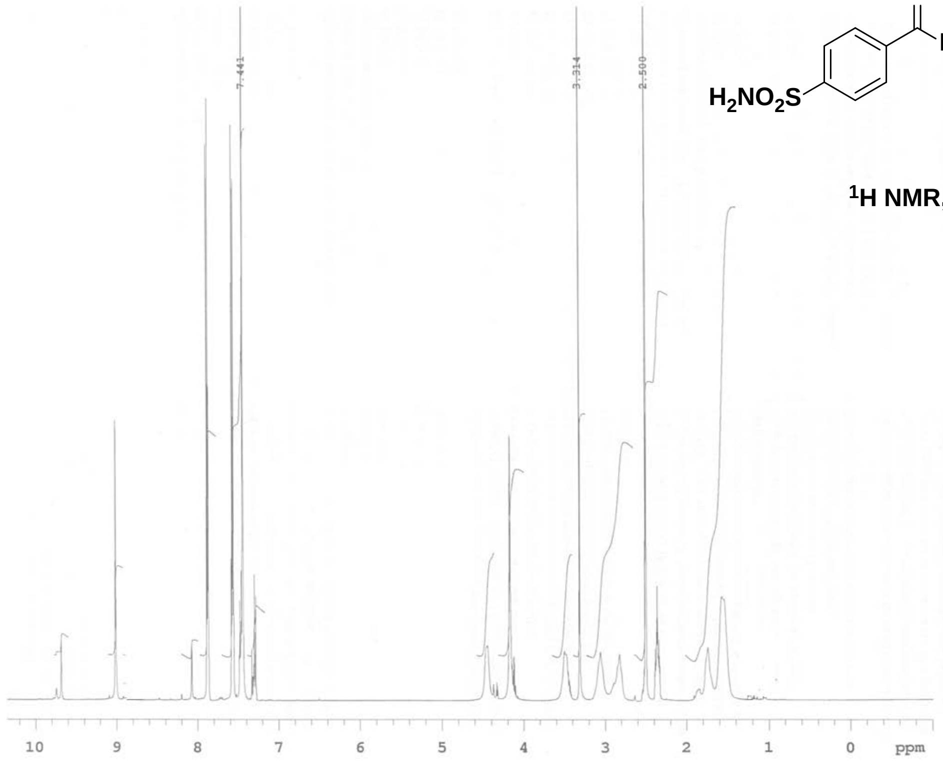

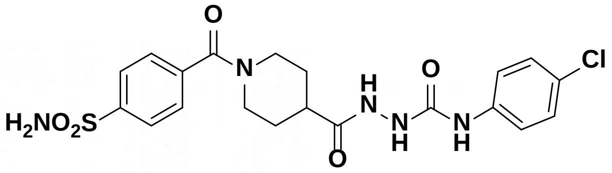

^{1}H NMR, DMSO-*d6, 5d.*

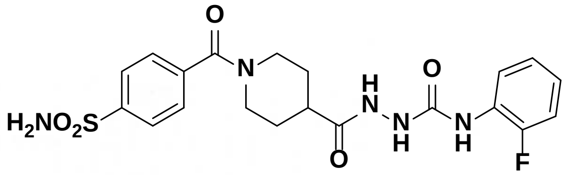

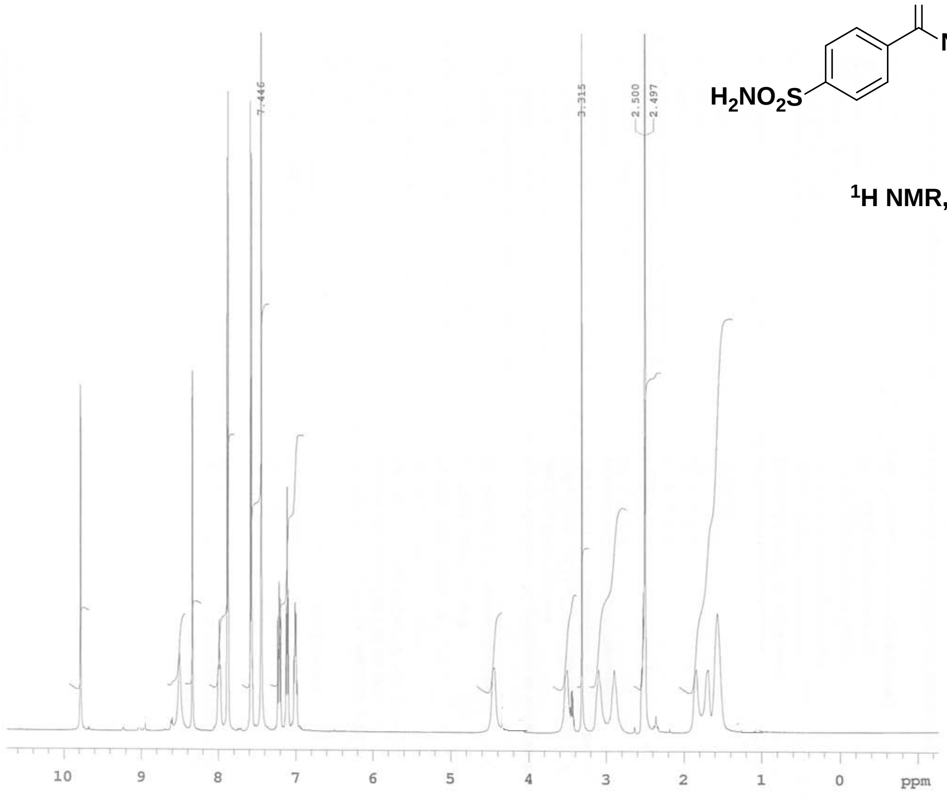

^{1}H NMR, DMSO-*d6, 5f.*

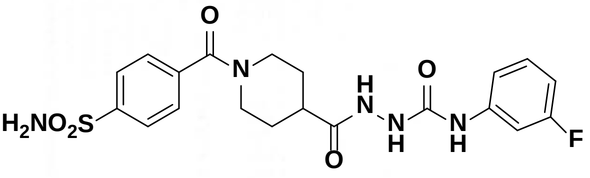

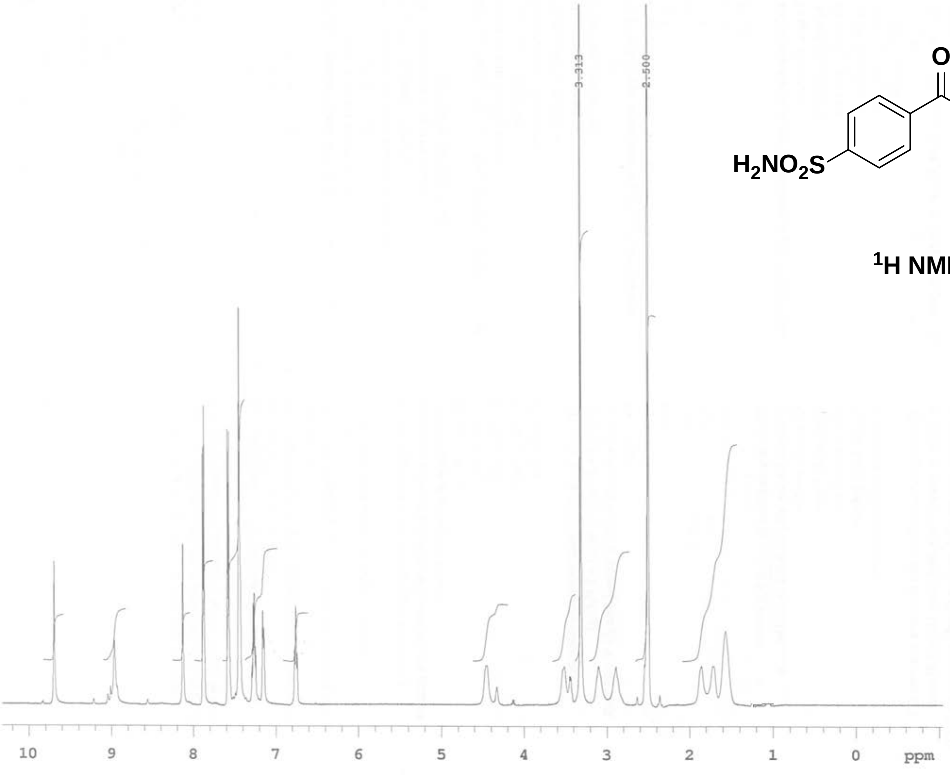

^{1}H NMR, DMSO-*d6, 5g*

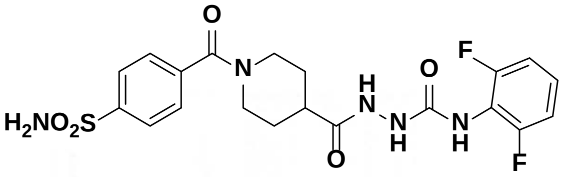

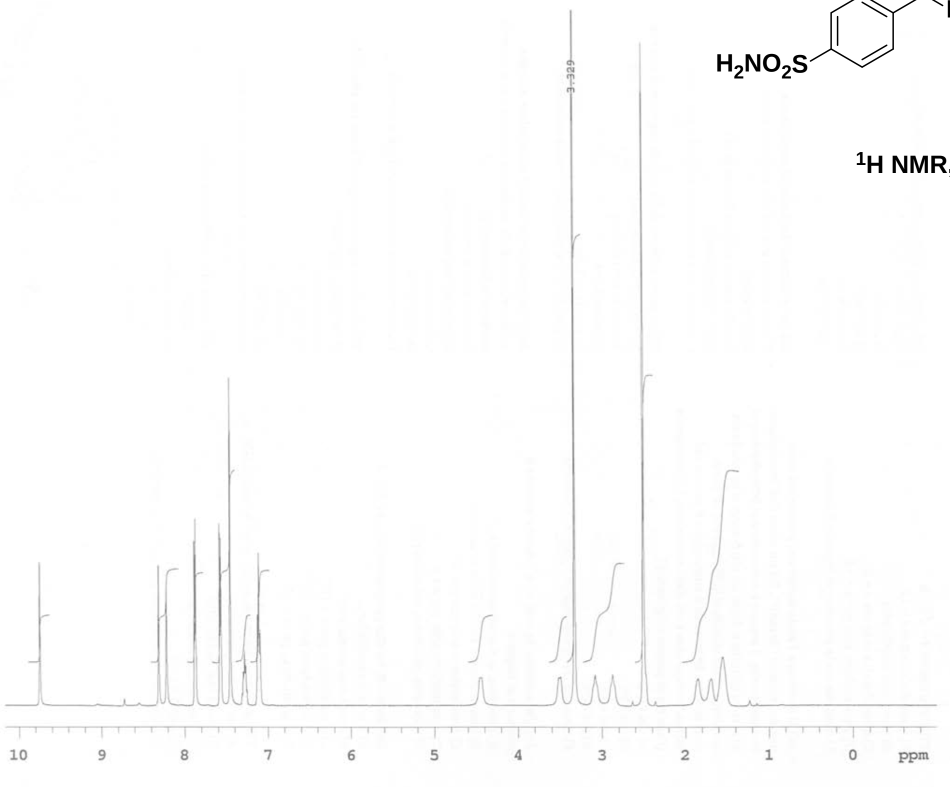

^{1}H NMR, DMSO-*d6, 5i*

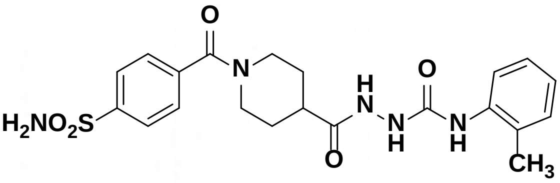

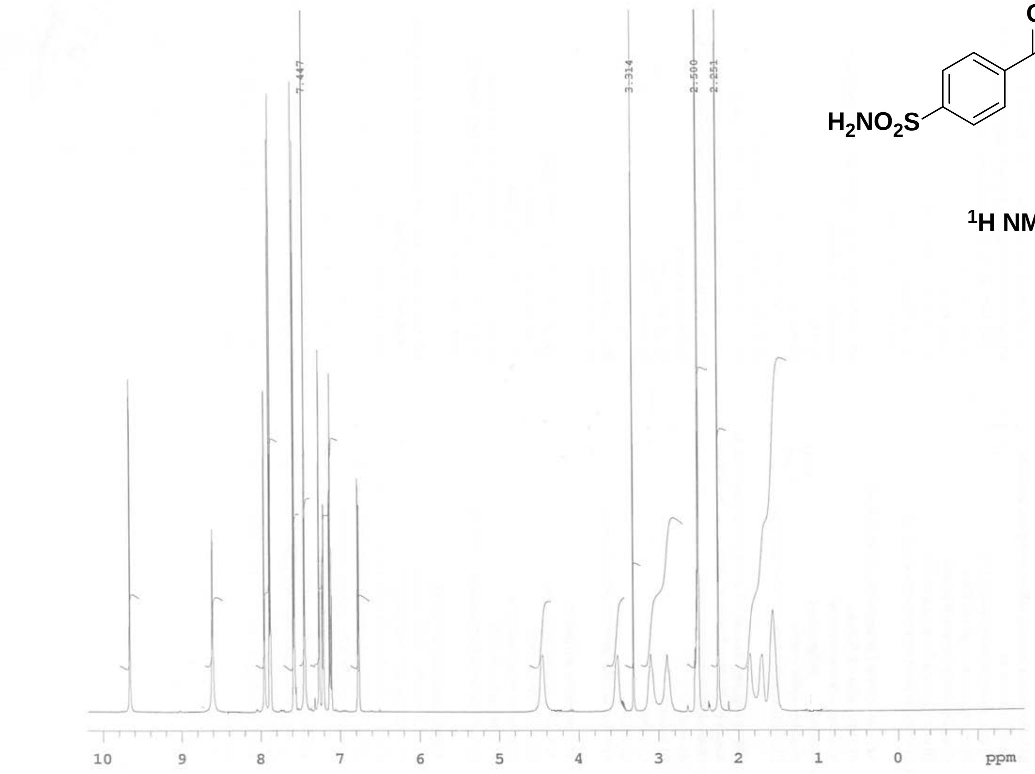

^{1}H NMR, DMSO-*d6, 5j*

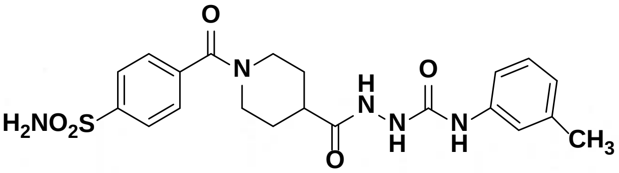

^{1}H NMR, DMSO-*d6, 5k*

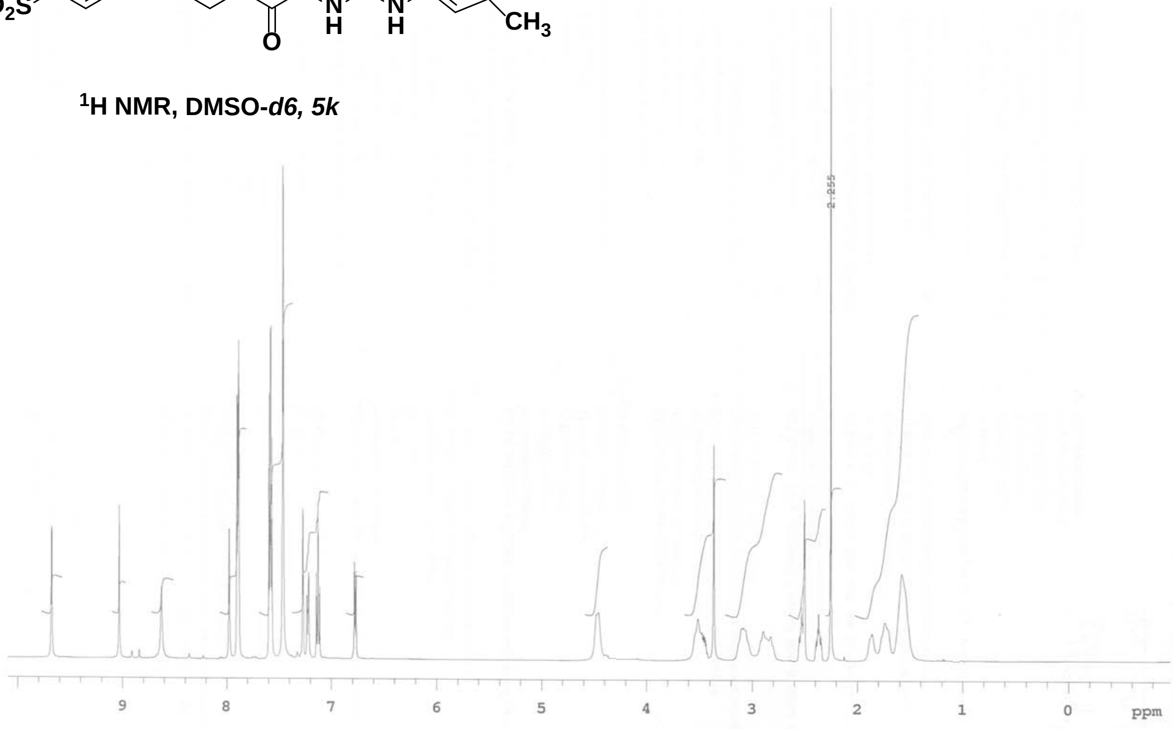

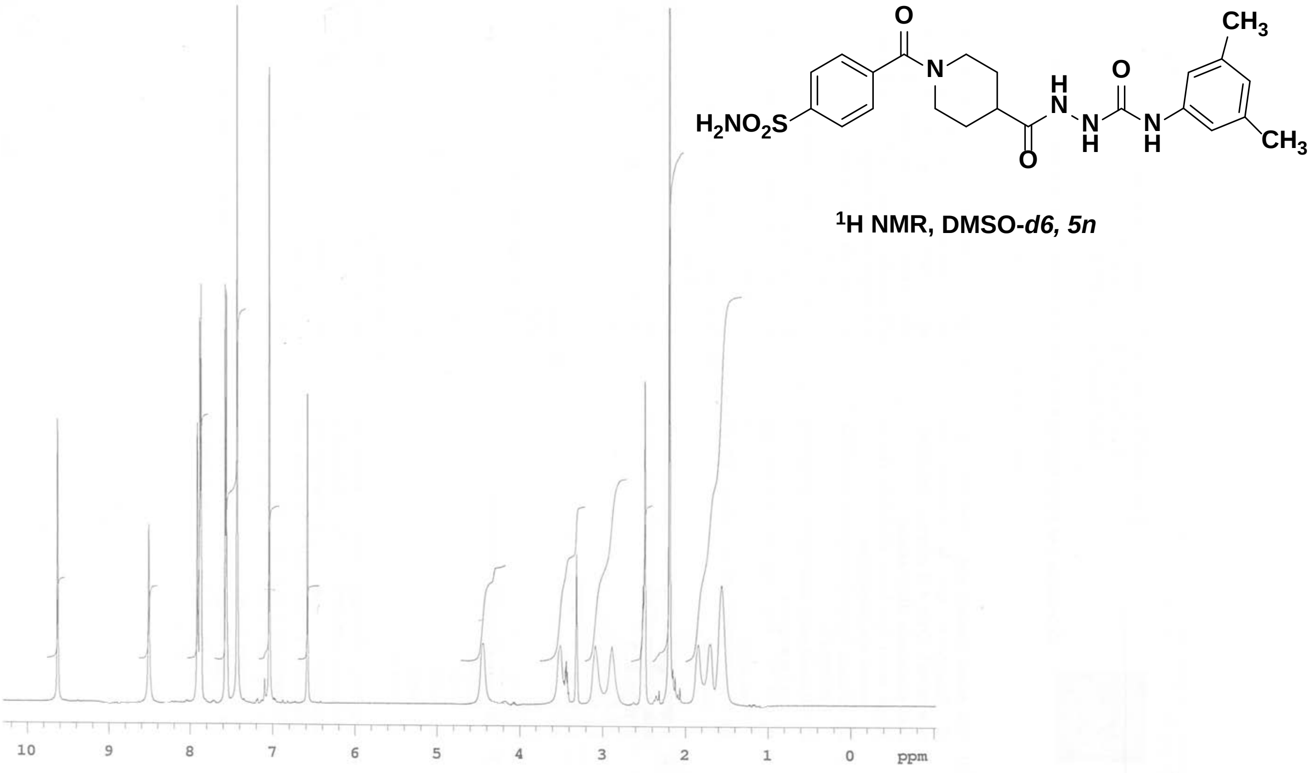

^{1}H NMR, DMSO-*d6, 5n*

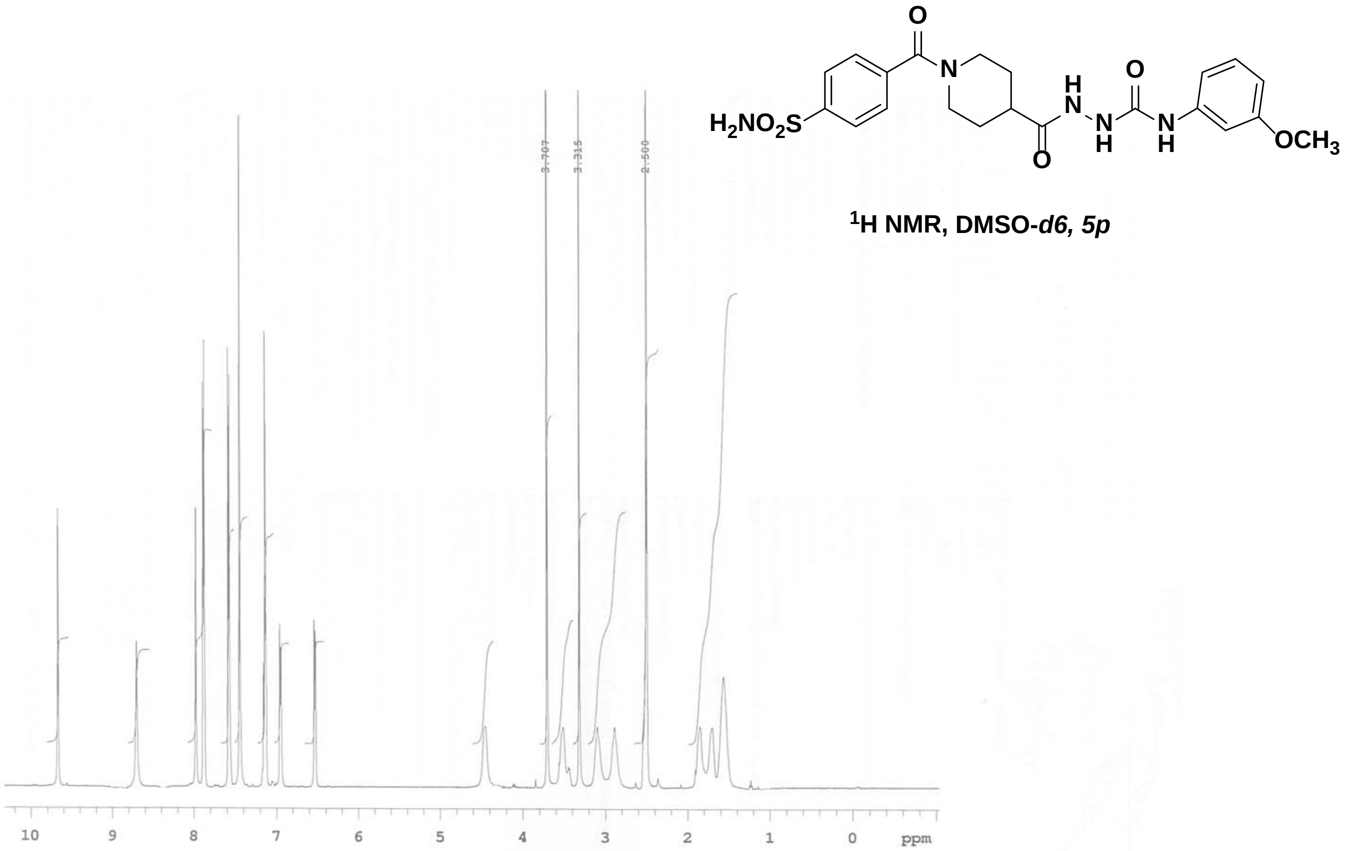

^{1}H NMR, DMSO-*d6, 5p*

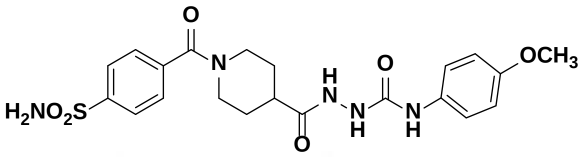

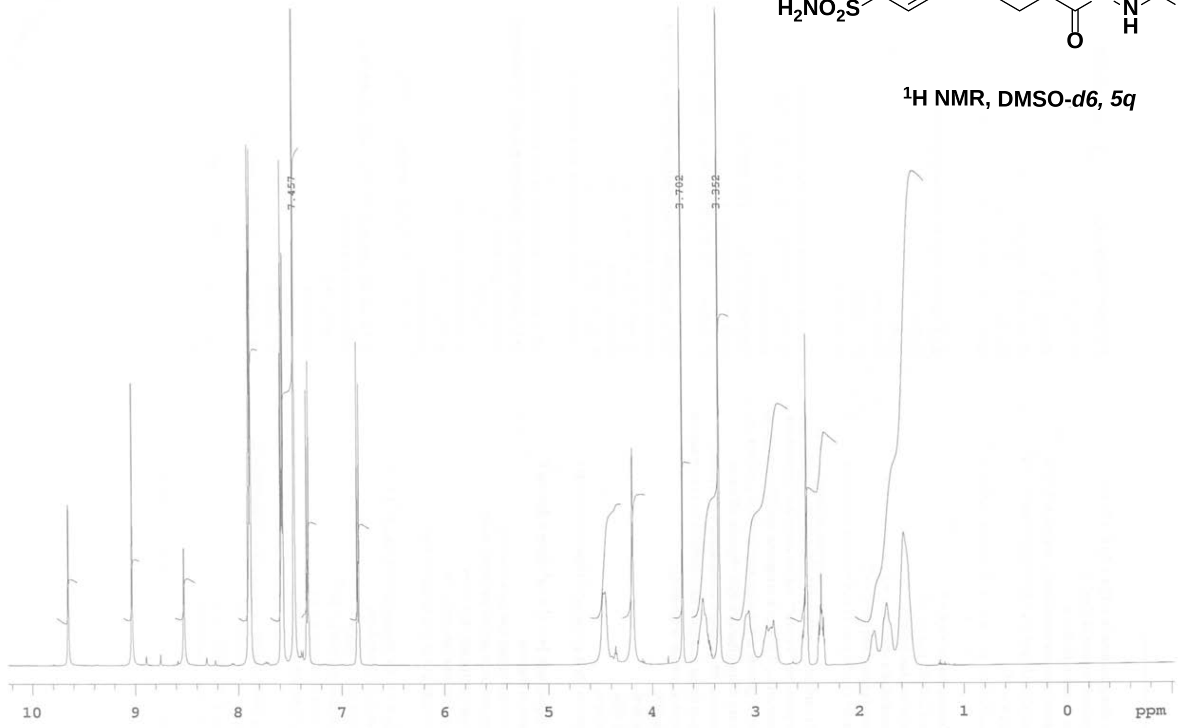

^{1}H NMR, DMSO-*d6, 5q*

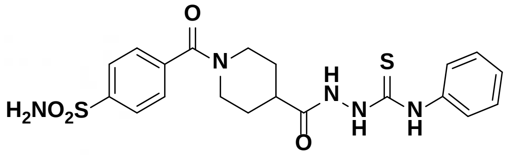

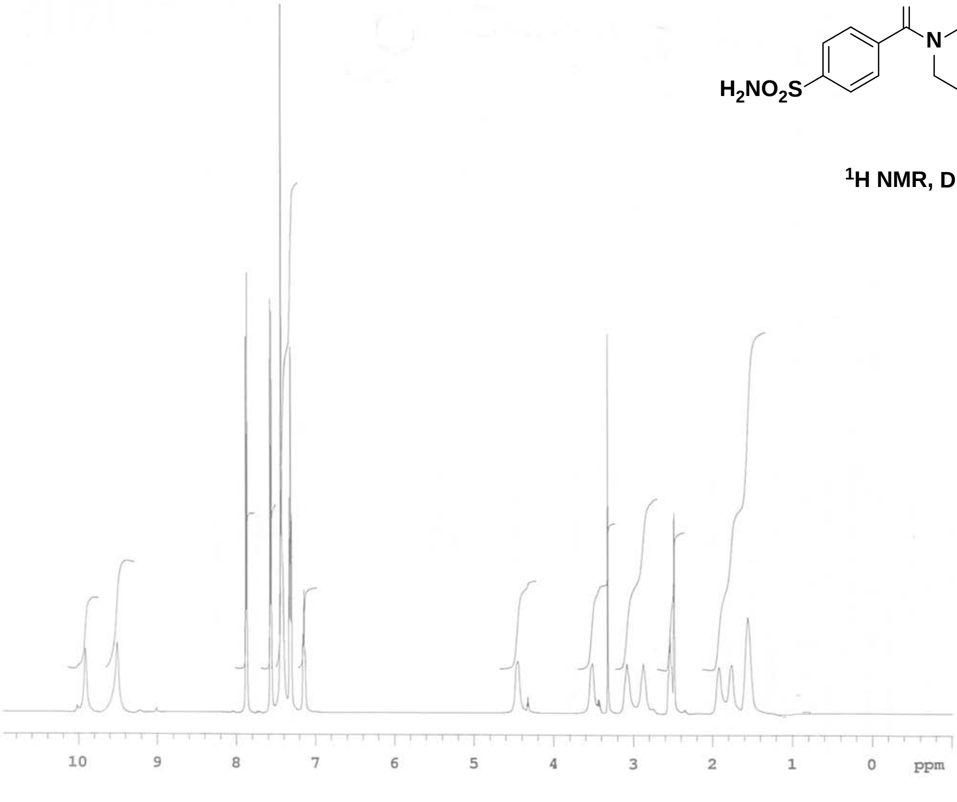

^{1}H NMR, DMSO-*d6, 6a*

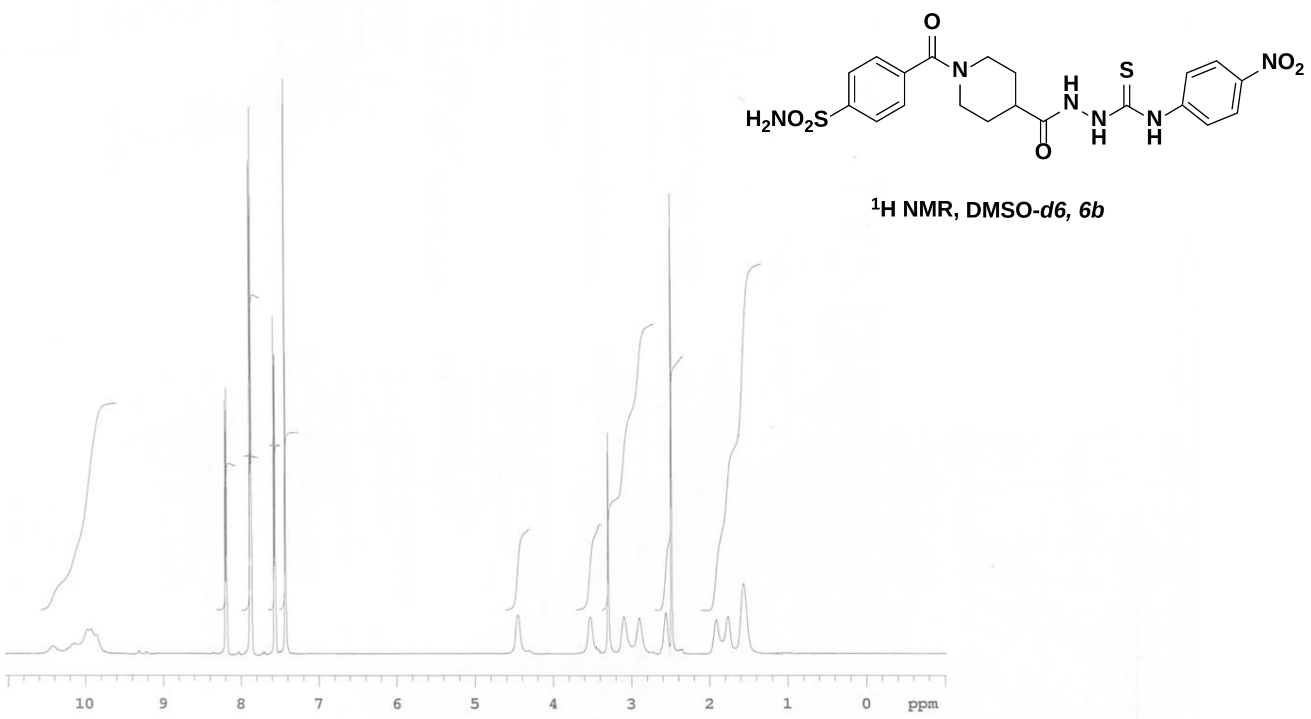

^{1}H NMR, DMSO-*d6, 6b*

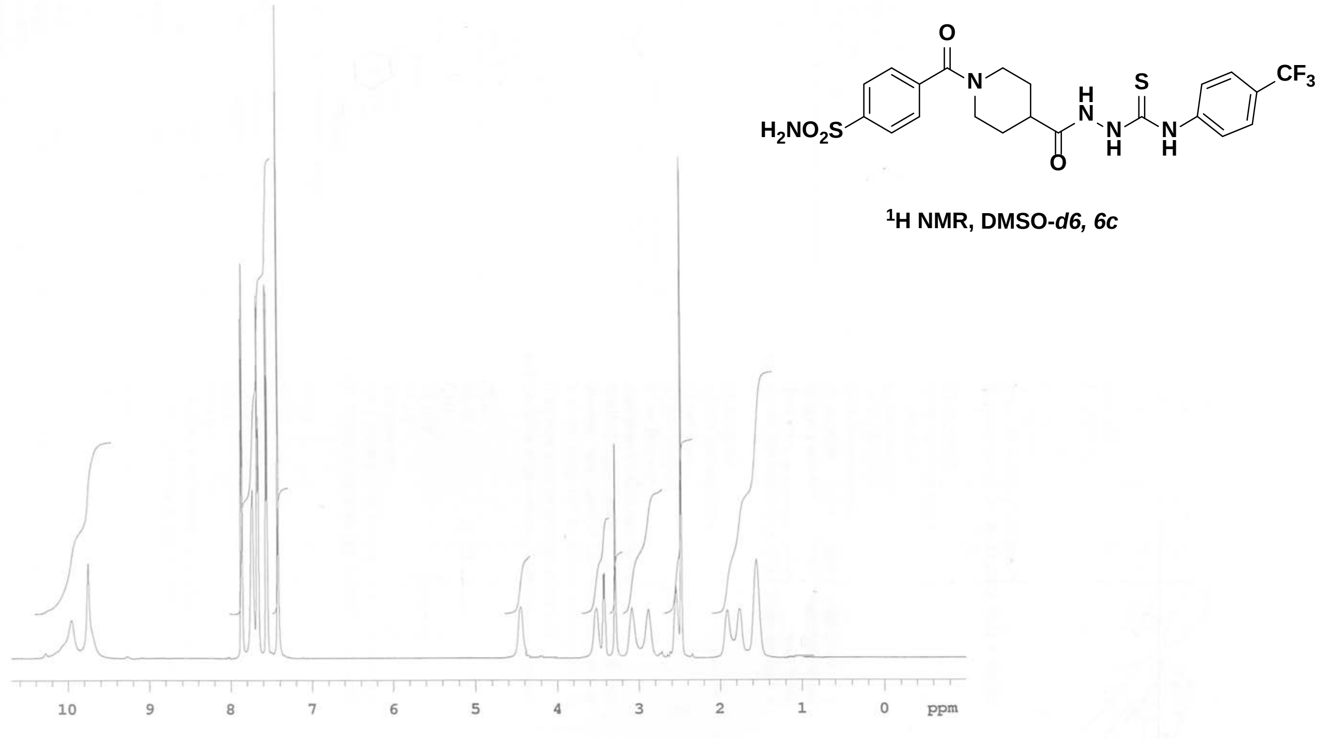

^{1}H NMR, DMSO-*d6, 6c*

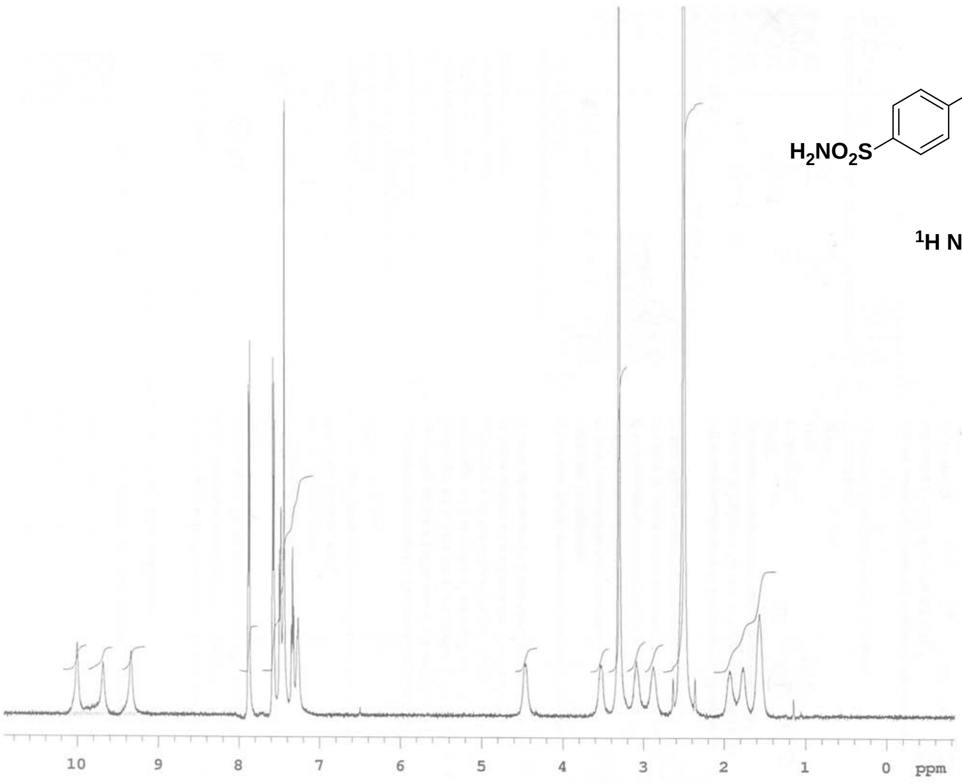

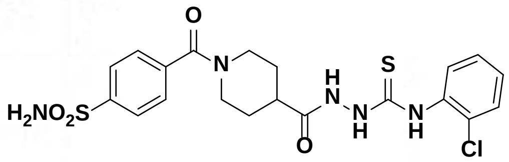

^{1}H NMR, DMSO-*d6, 6d*

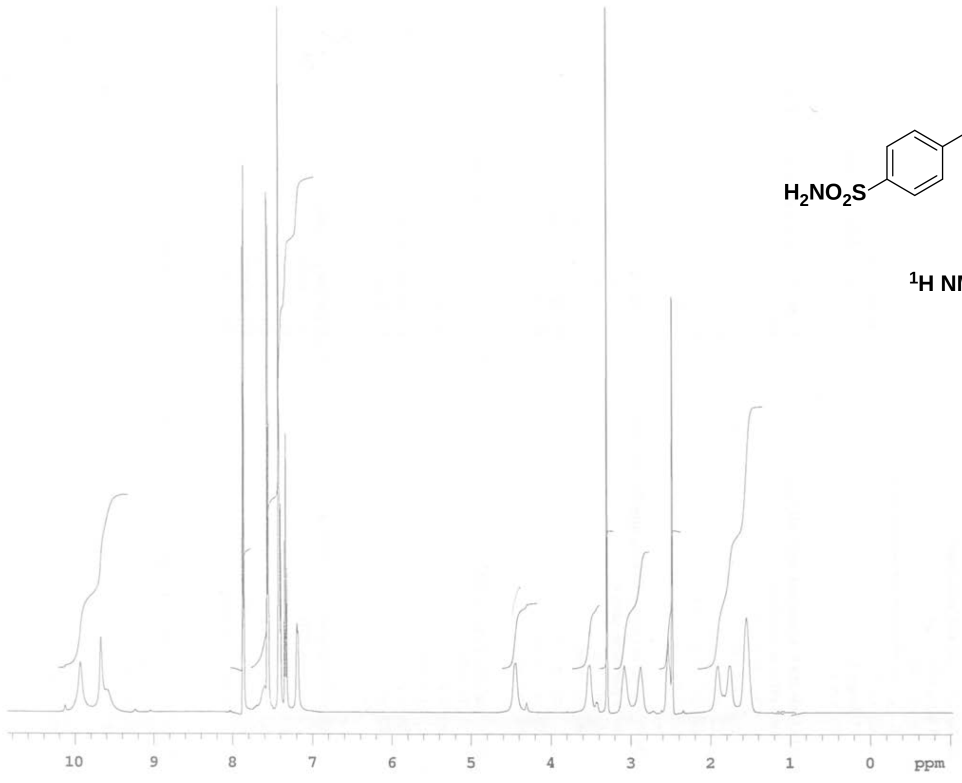

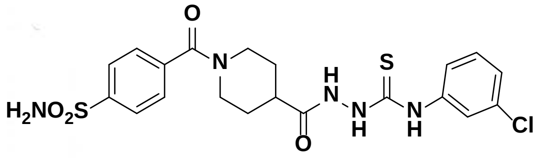

^{1}H NMR, DMSO-*d6, 6e*

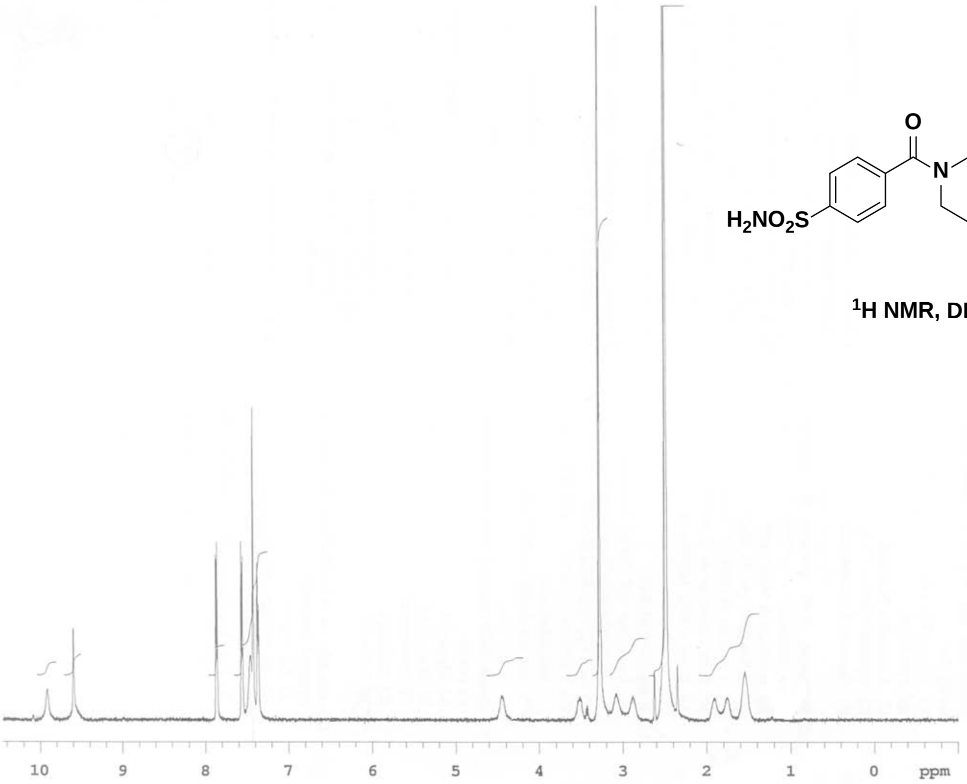

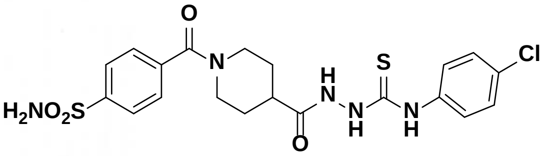

^{1}H NMR, DMSO-*d6, 6f*

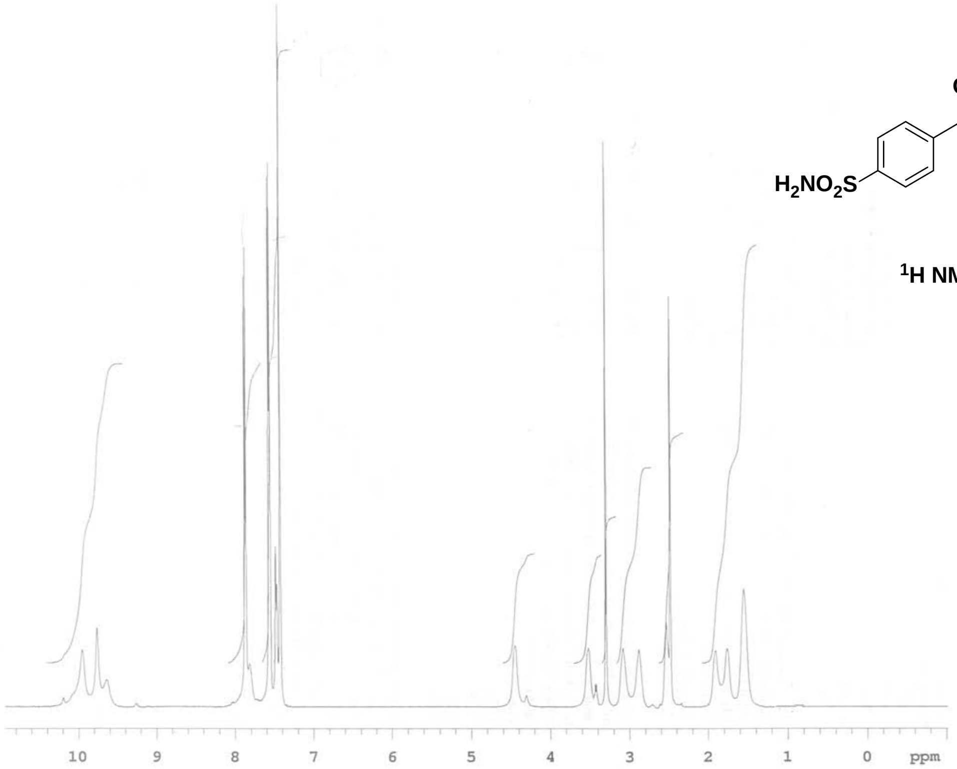

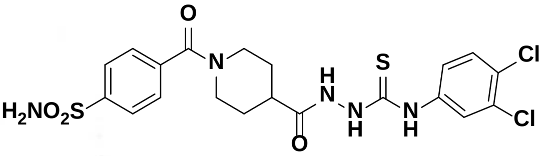

^{1}H NMR, DMSO-*d6, 6g*

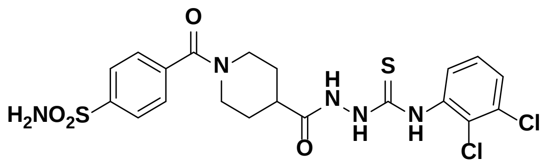

^{1}H NMR, DMSO-*d6, 6h*

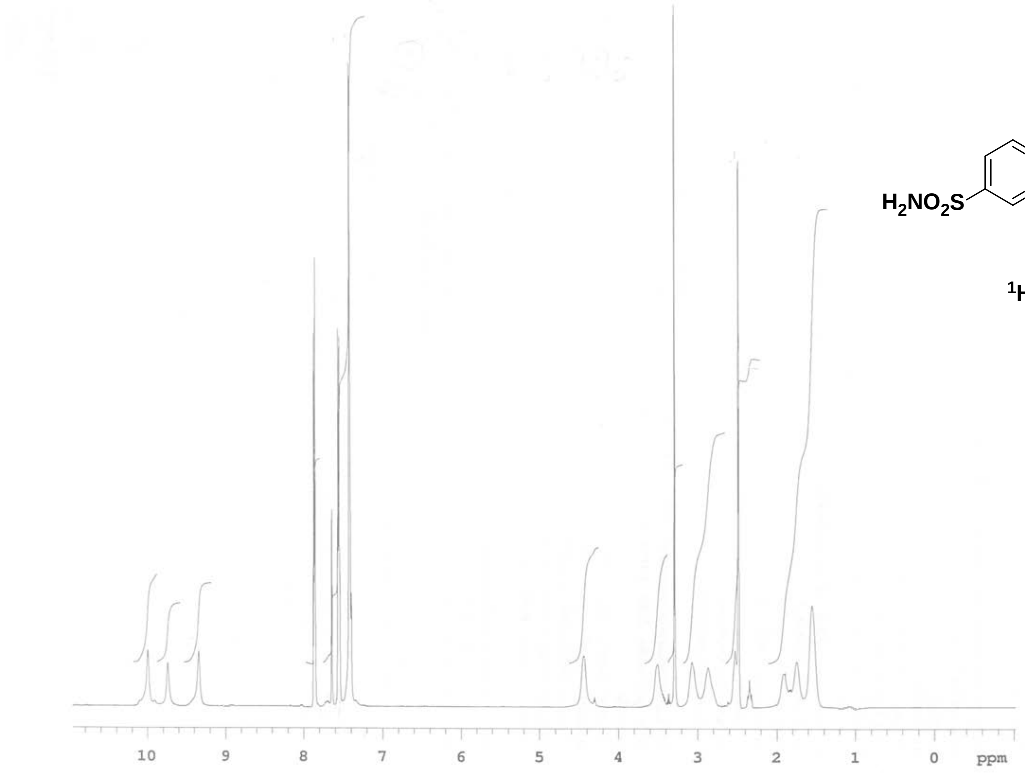

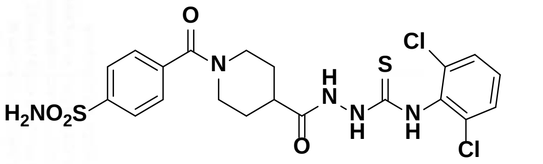

^{1}H NMR, DMSO-*d6, 6i*

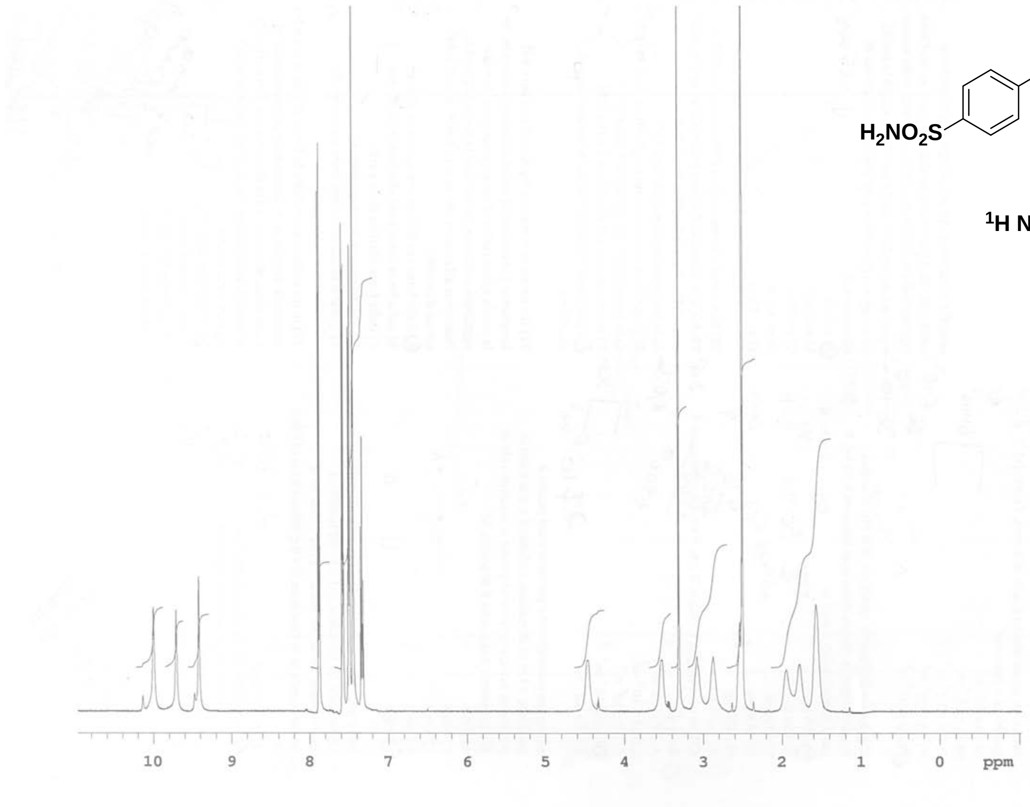

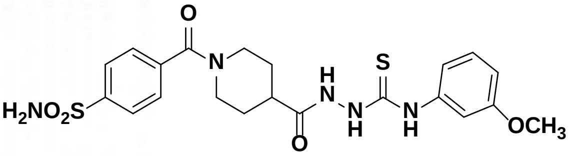

^{1}H NMR, DMSO-*d6, 6k*

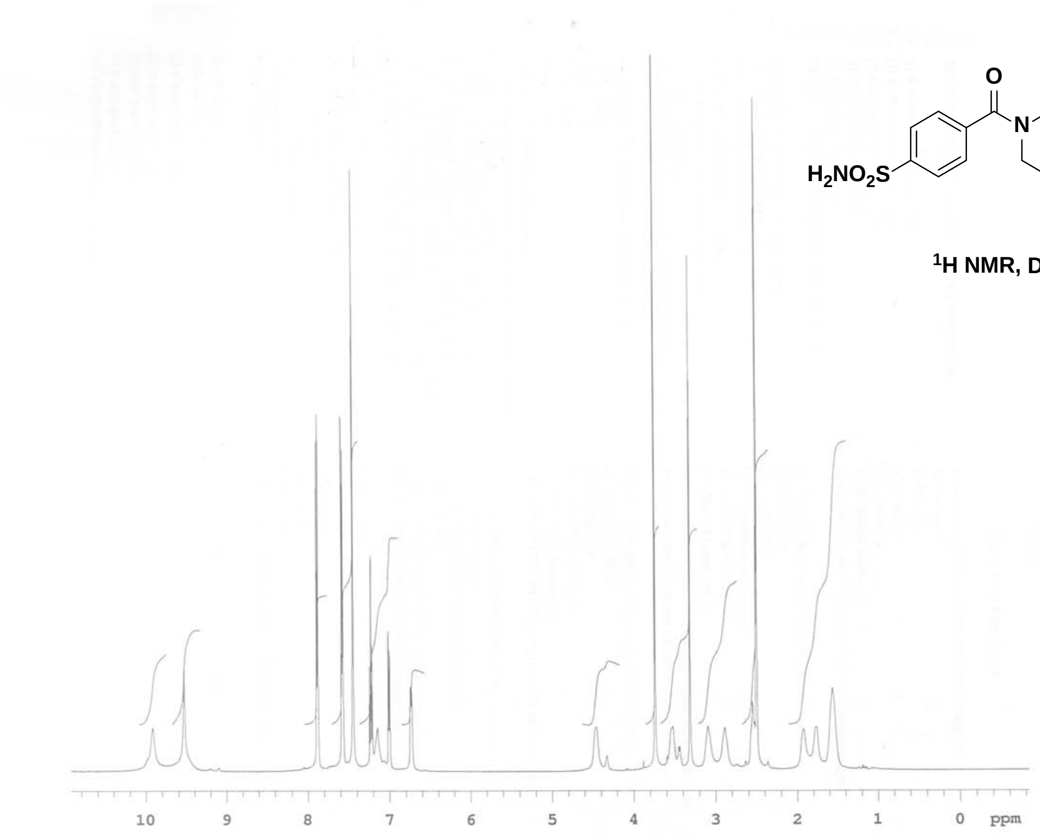

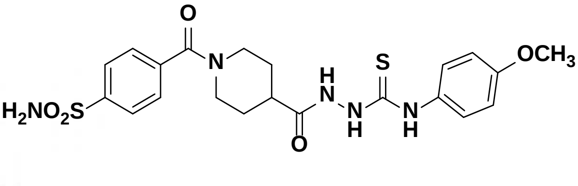

^{1}H NMR, DMSO-*d6, 6l*

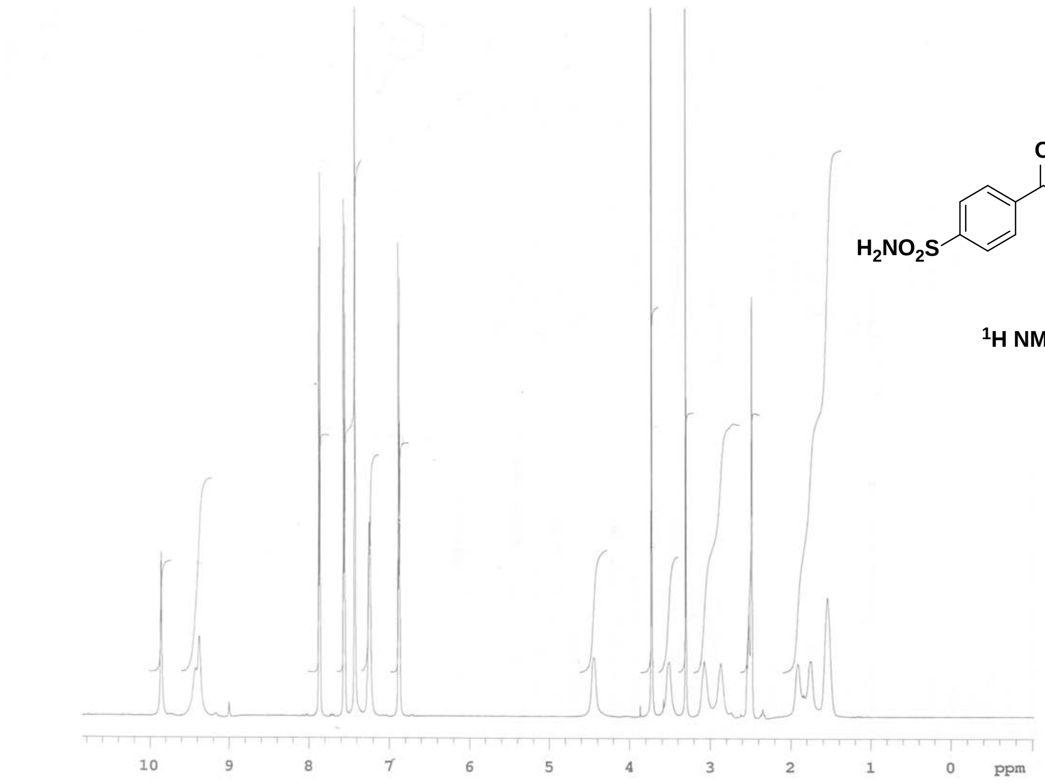

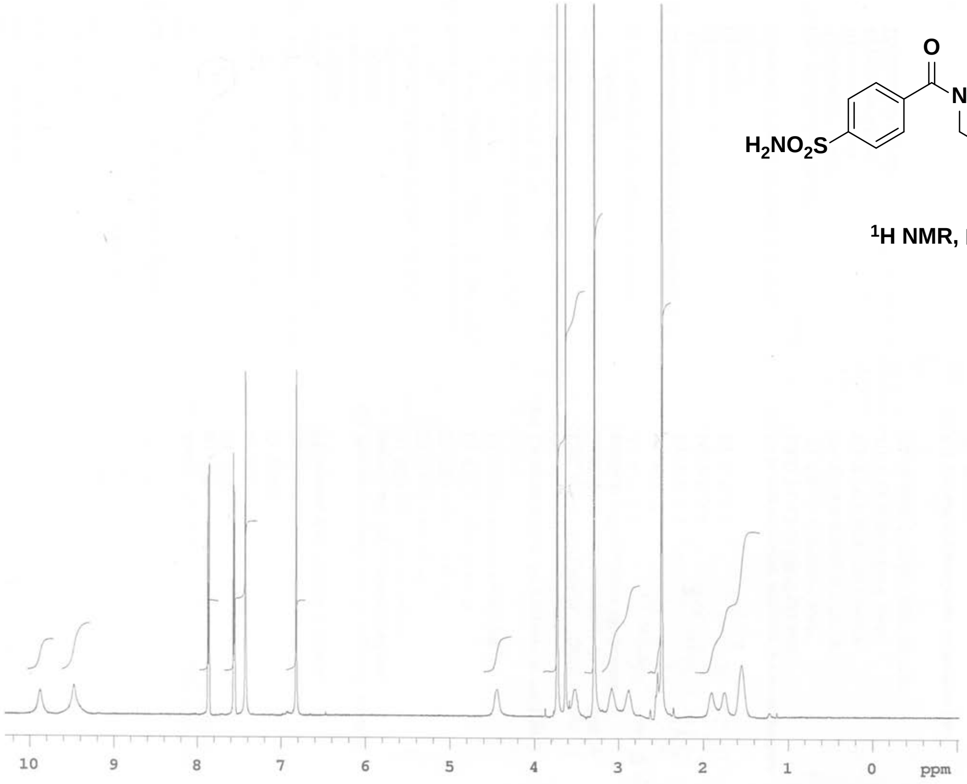

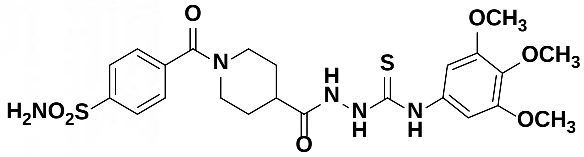

^{1}H NMR, DMSO-*d6, 6m*

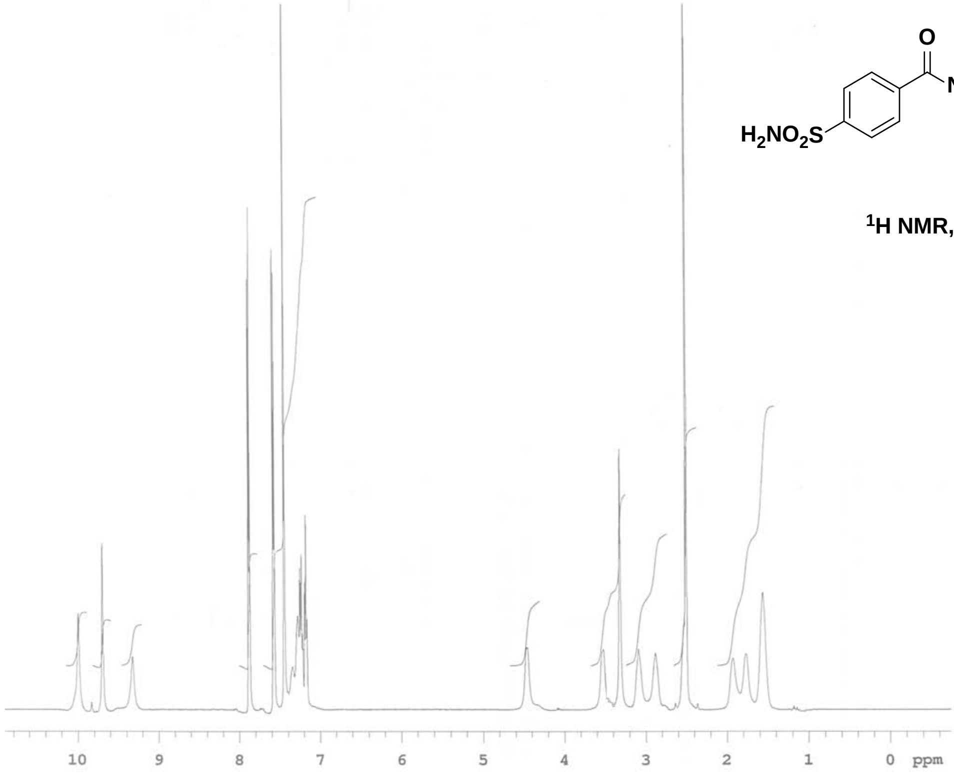

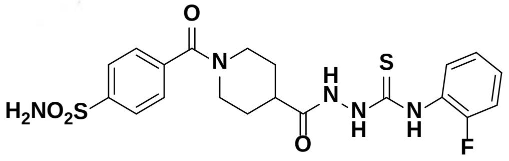

^{1}H NMR, DMSO-*d6, 6n*

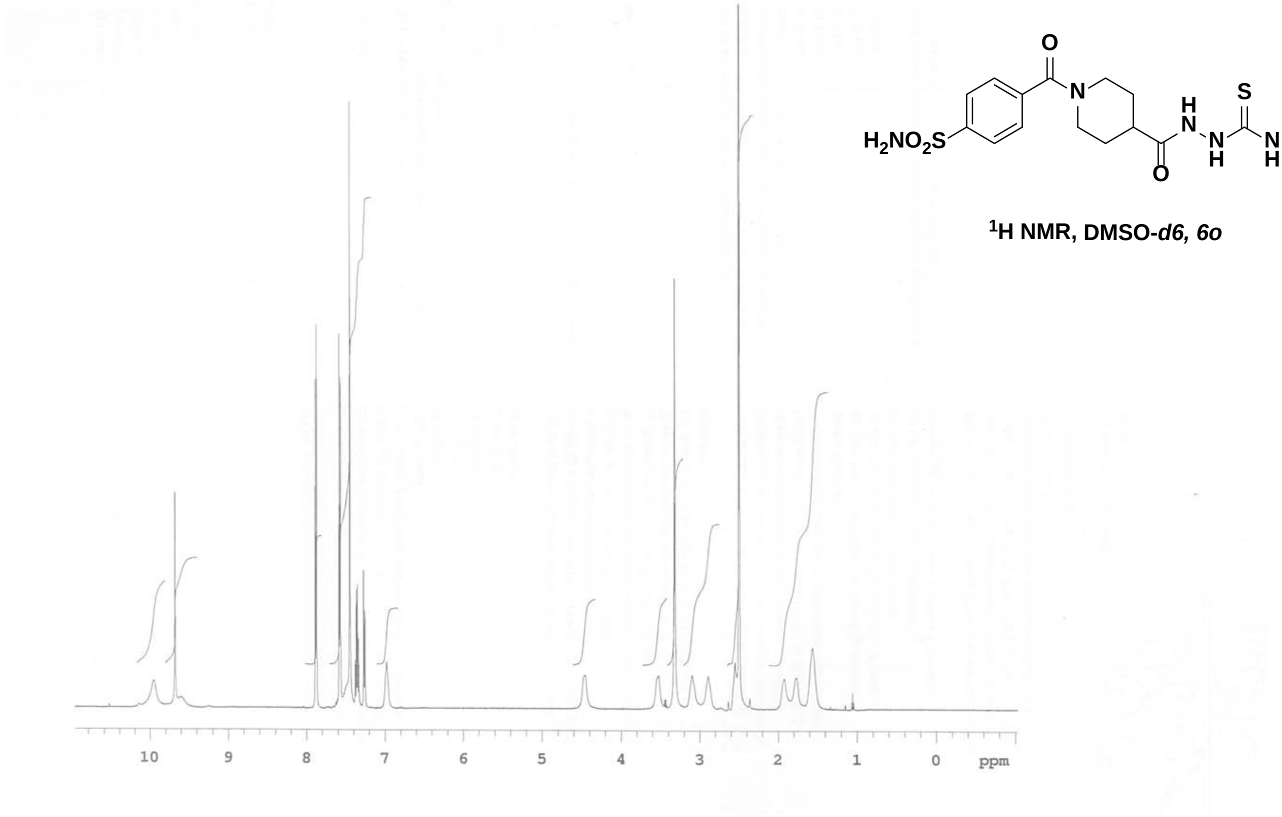

^{1}H NMR, DMSO-*d6, 6o*

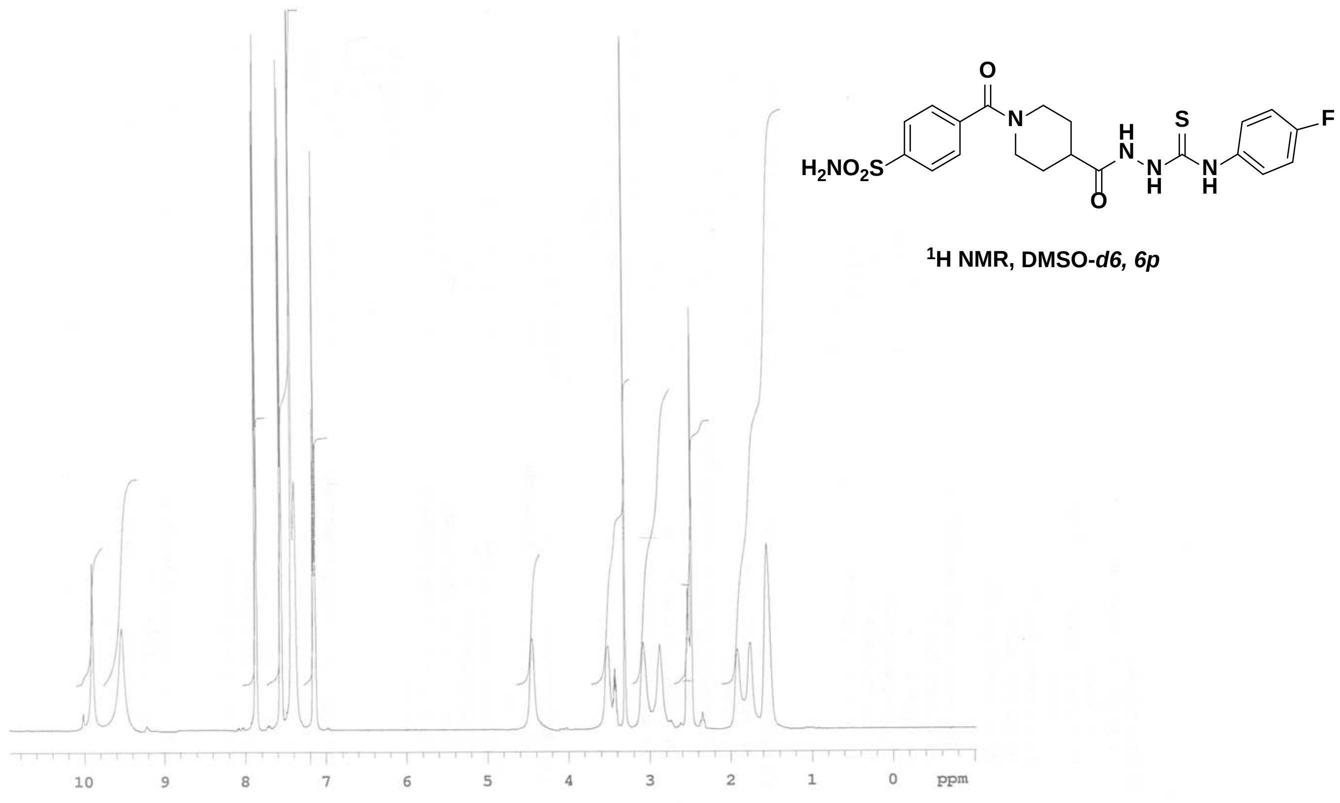

^{1}H NMR, DMSO-*d6, 6p*

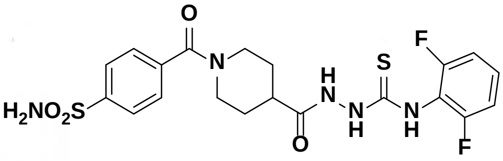

^{1}H NMR, DMSO-*d6, 6q*

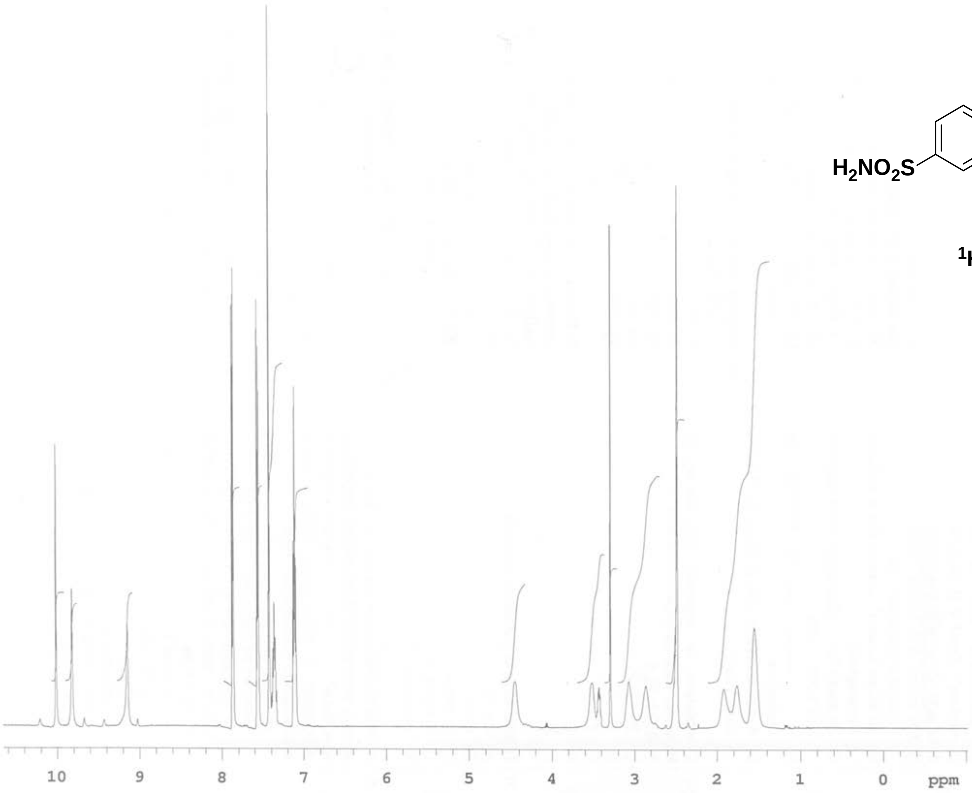

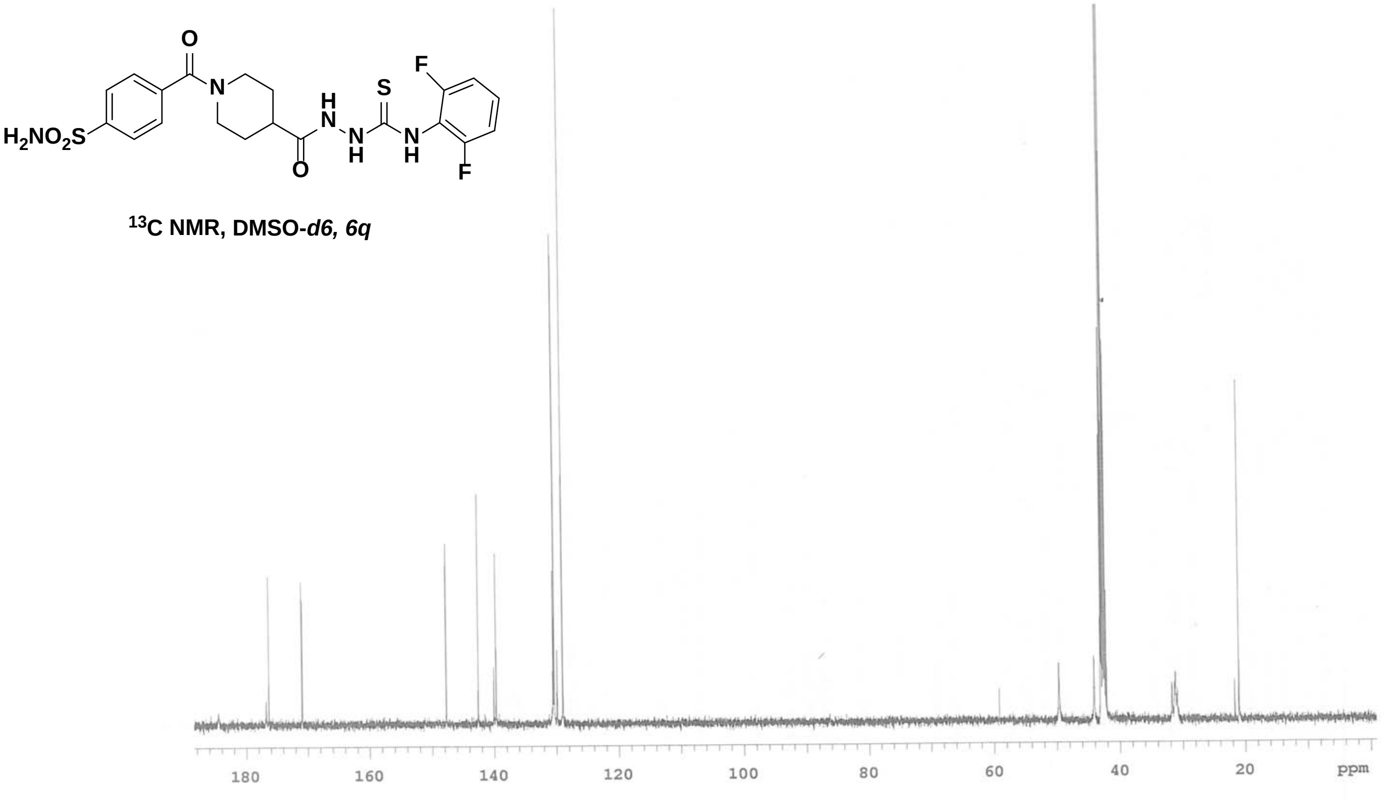

^{13}C NMR, DMSO-*d6, 6q*

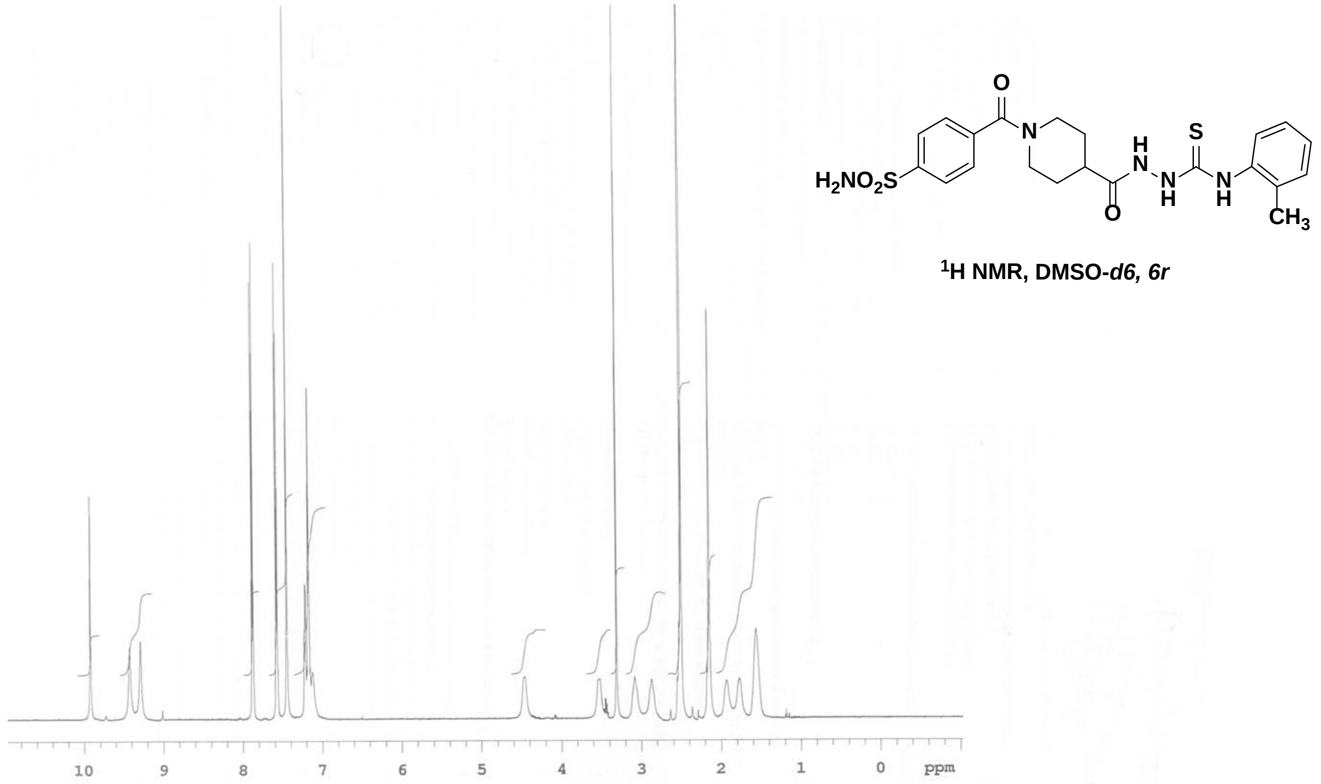

^{1}H NMR, DMSO-*d6, 6r*

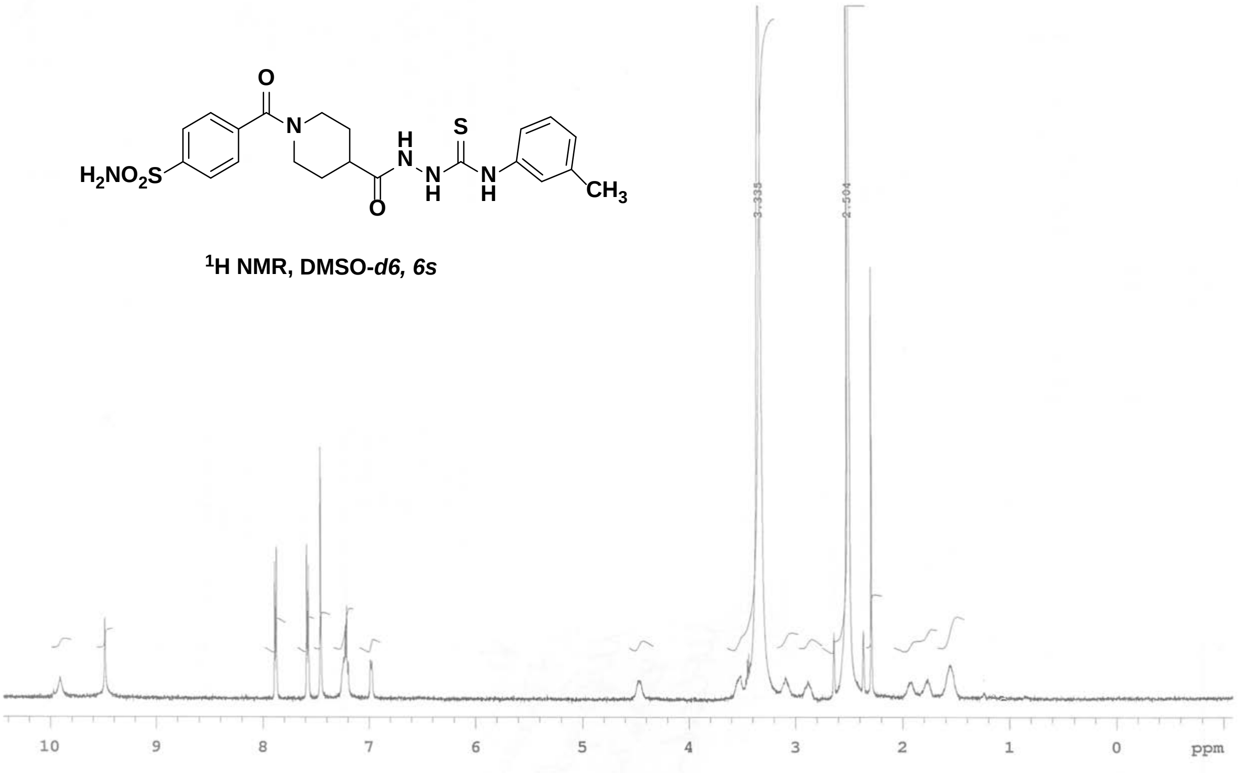

^{1}H NMR, DMSO-*d6, 6s*

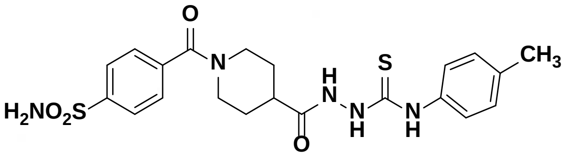

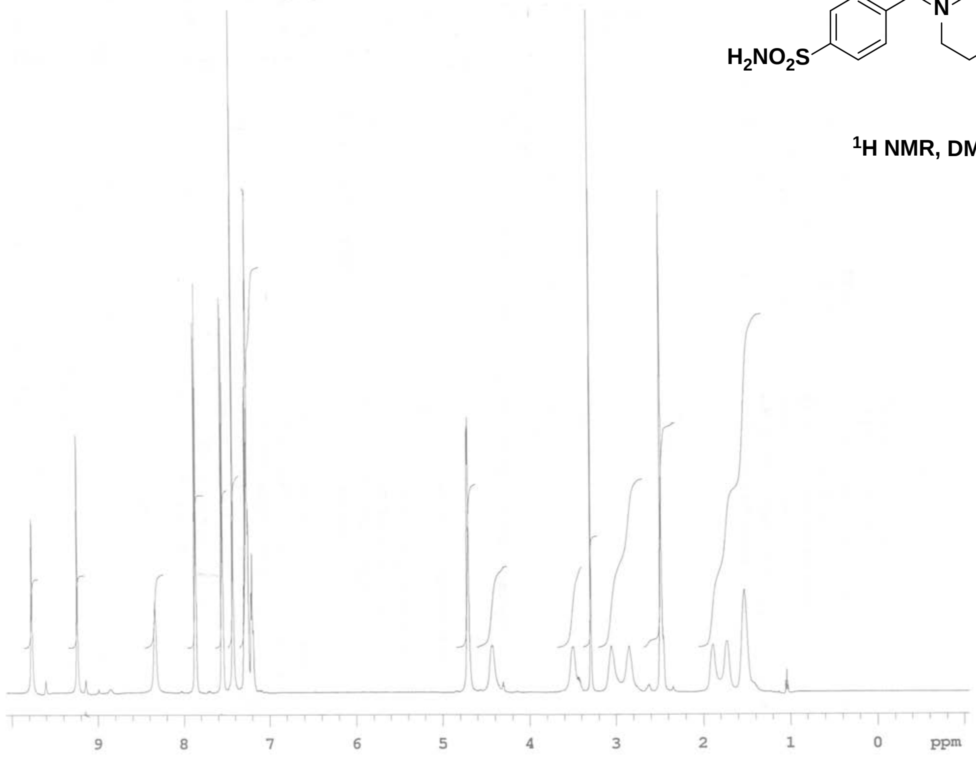

^{1}H NMR, DMSO-*d6, 6t*

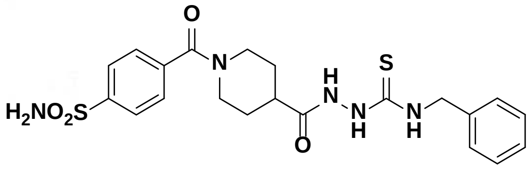

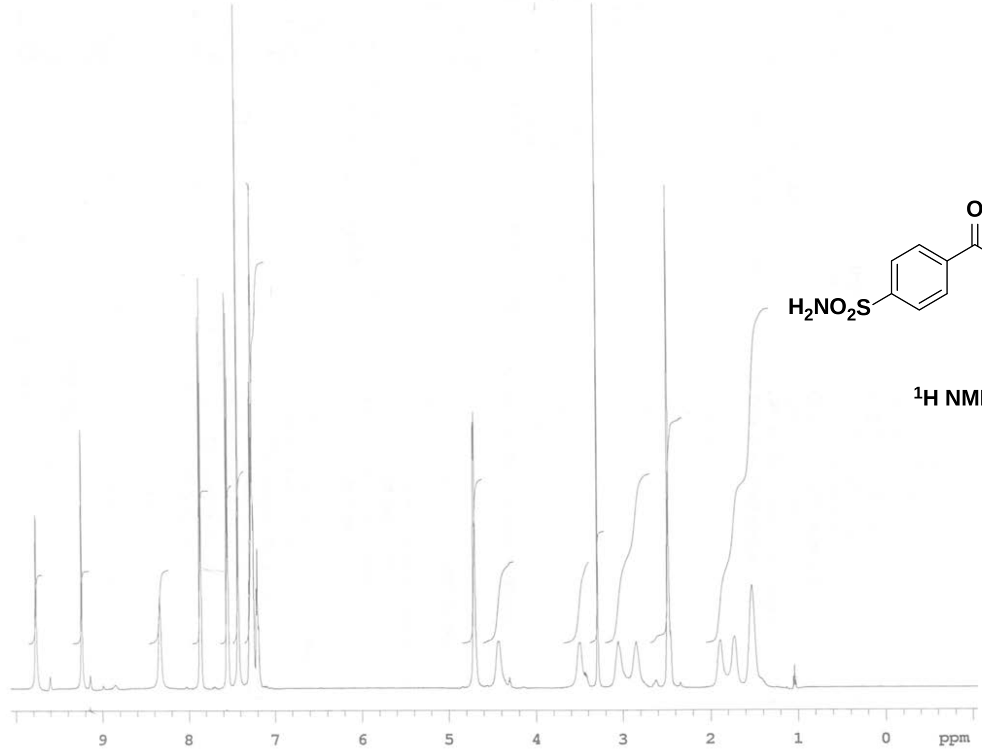

^{1}H NMR, DMSO-*d6, 6v*

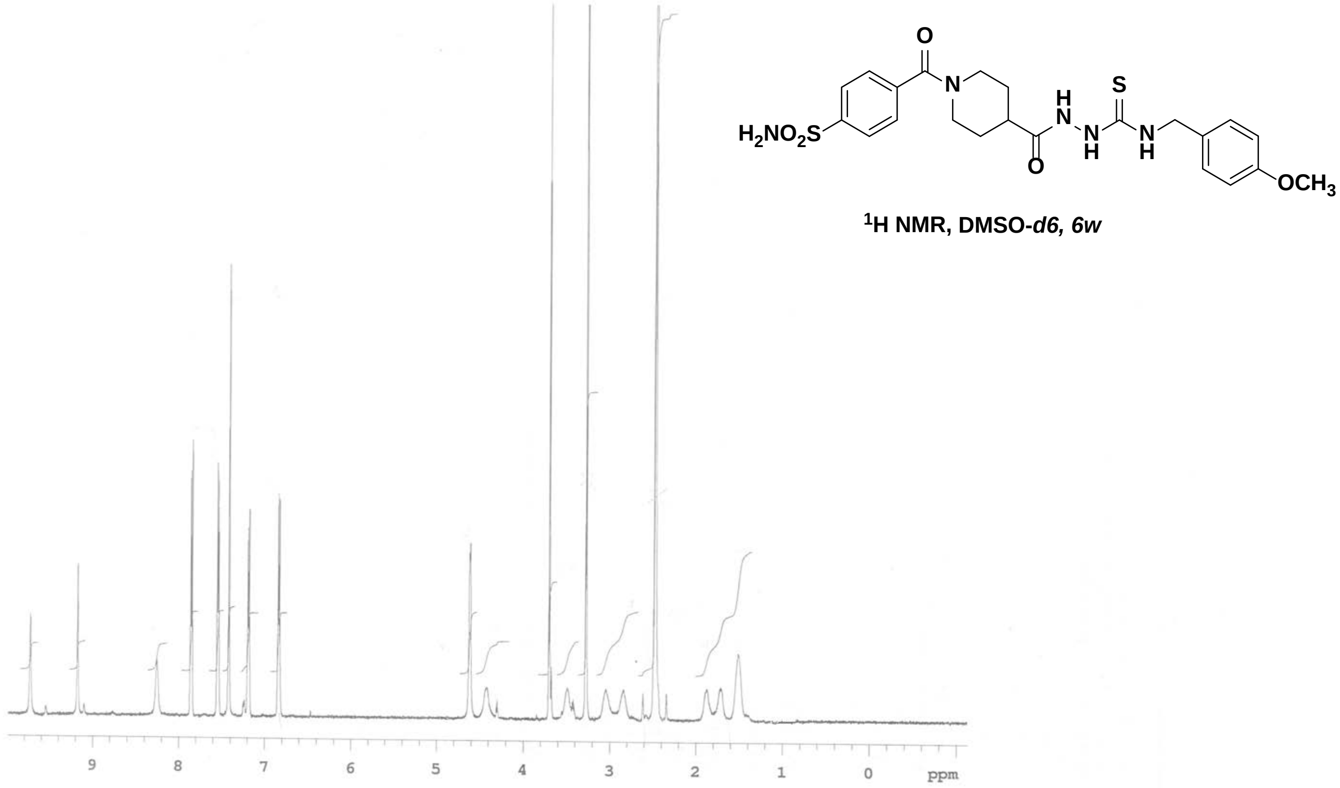

^{1}H NMR, DMSO-*d6, 6w*

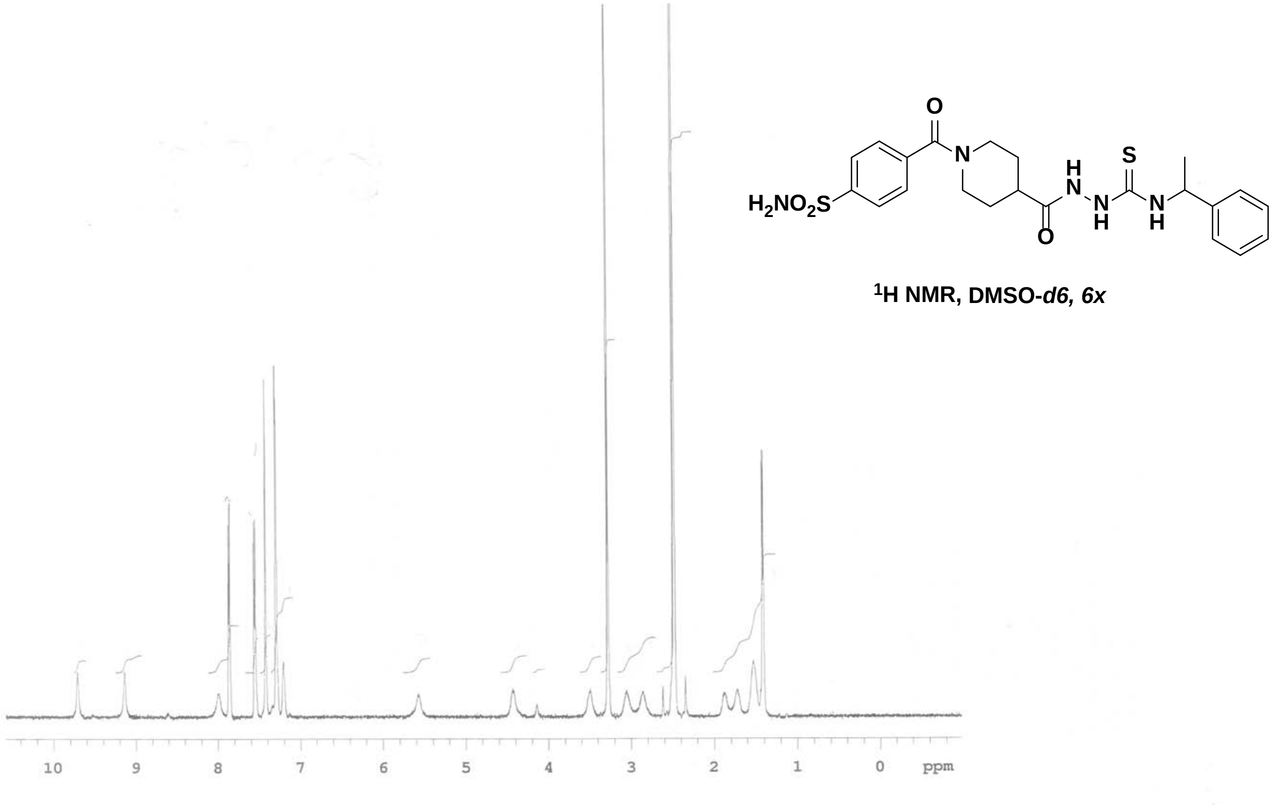

^{1}H NMR, DMSO-*d6, 6x*

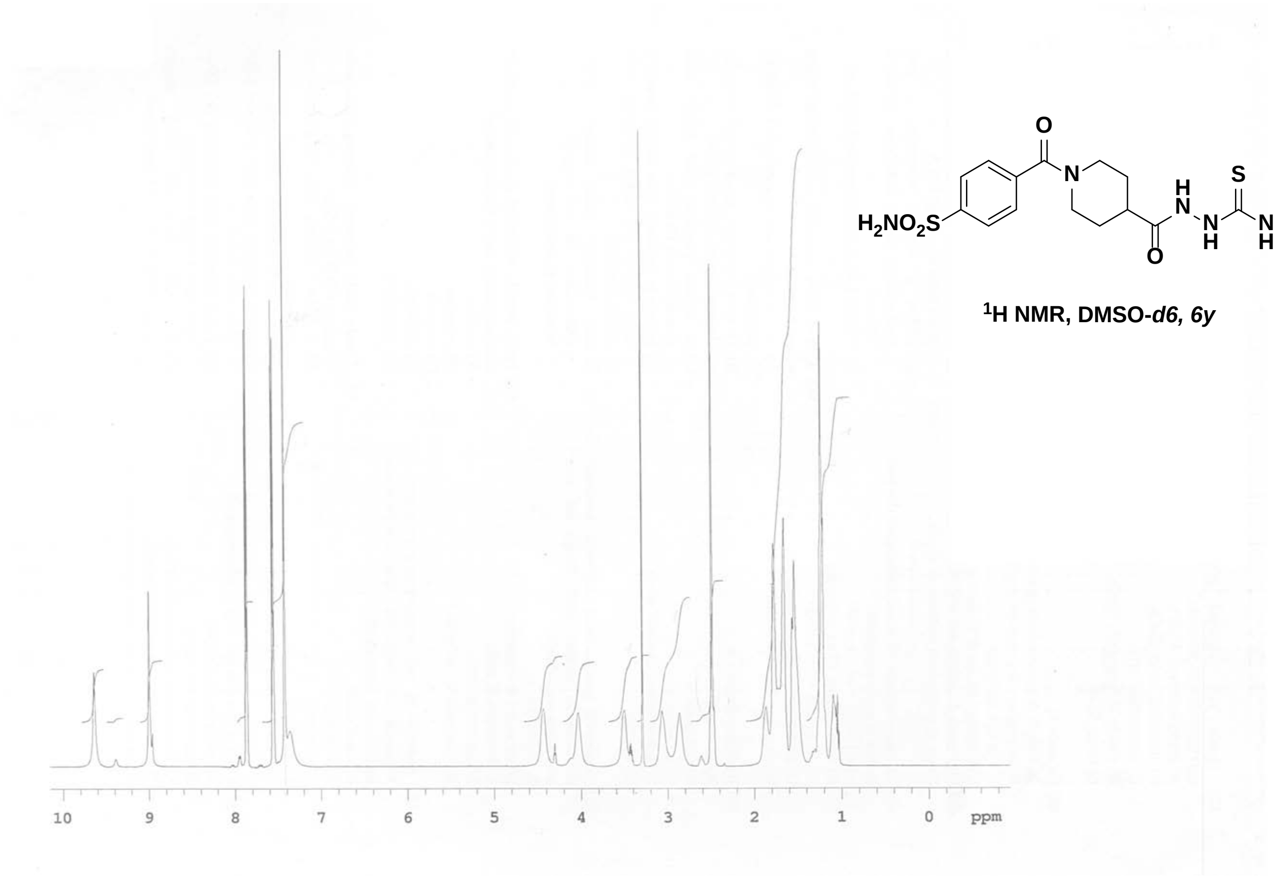

^{1}H NMR, DMSO-*d6, 6y*

compound 5g

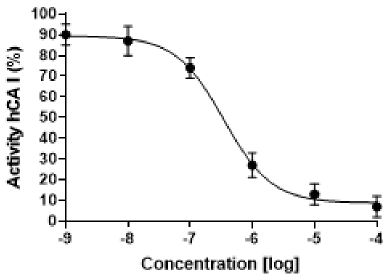

compound 5g

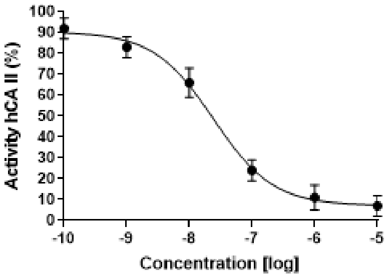

compound 5g

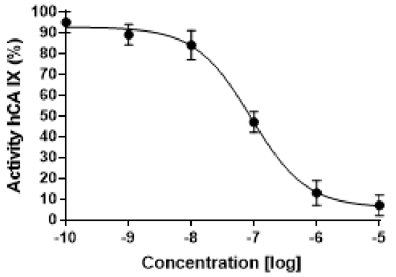

compound 5g

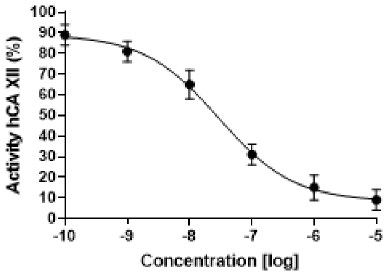

compound 5m

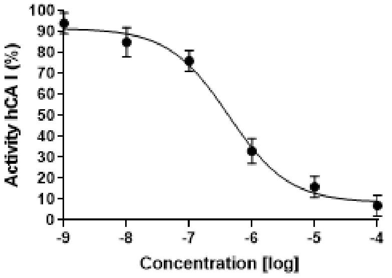

compound 5m

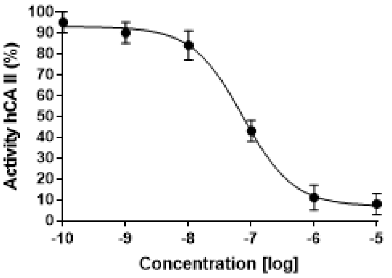

compound 5m

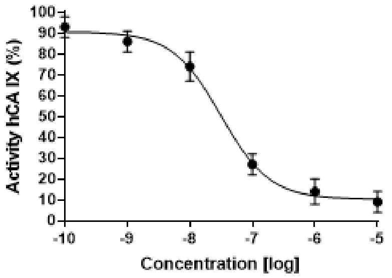

compound 5m

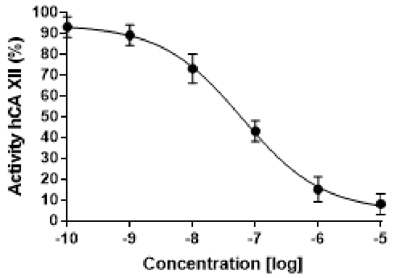

compound 5o

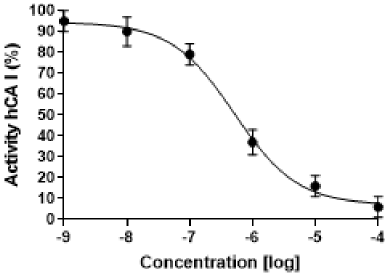

compound 5o

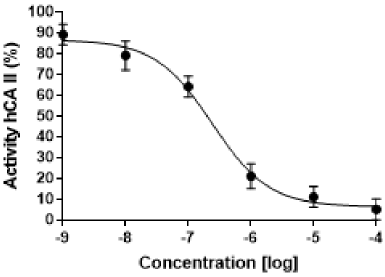

compound 5o

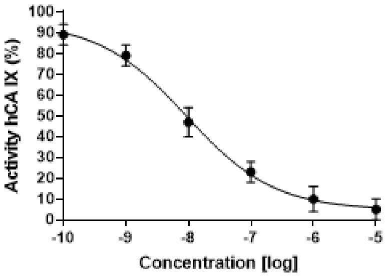

compound 5o

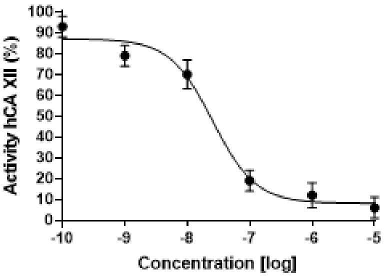

compound 5q

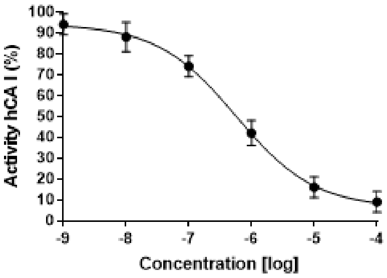

compound 5q

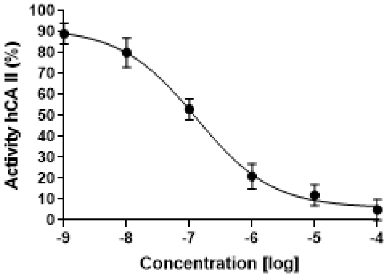

compound 5q

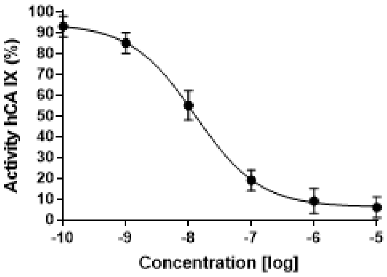

compound 5q

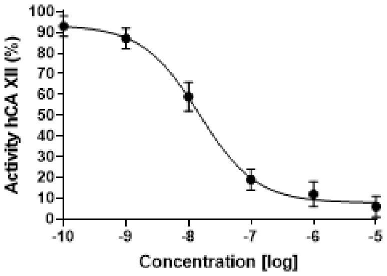

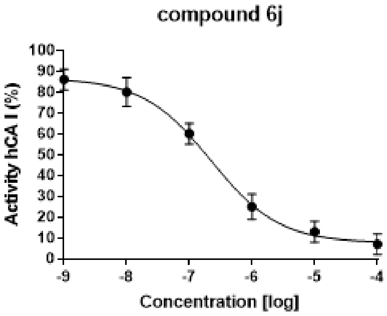
compound 6j
Activity hCA I (%)
Concentration [log]

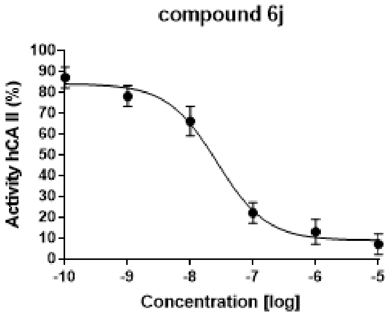
compound 6j
Activity hCA II (%)
Concentration [log]

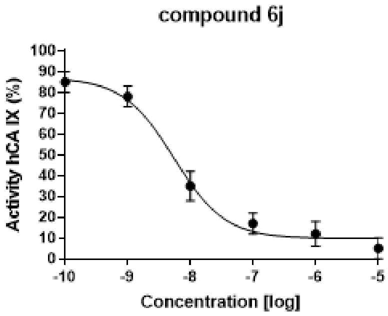
compound 6j
Activity hCA IX (%)
Concentration [log]

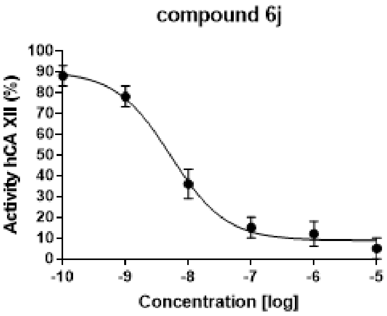
compound 6j
Activity hCA XII (%)
Concentration [log]

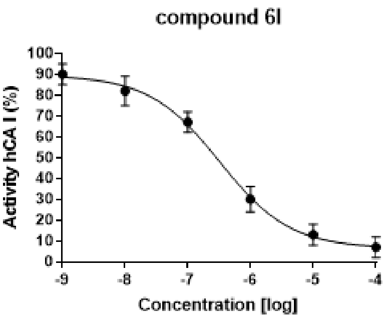
compound 6l
Activity hCA I (%)
Concentration [log]

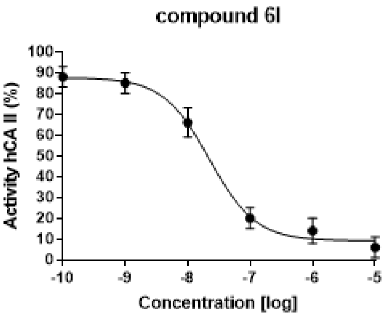
compound 6l
Activity hCA II (%)
Concentration [log]

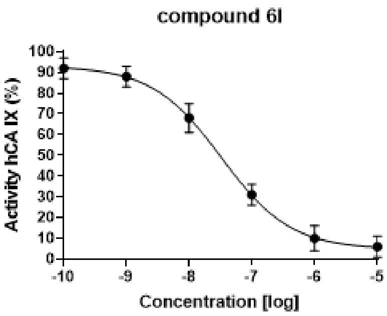
compound 6l
Activity hCA IX (%)
Concentration [log]

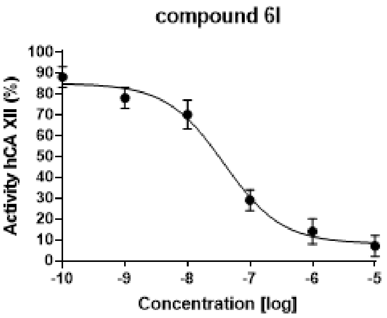
compound 6l
Activity hCA XII (%)
Concentration [log]

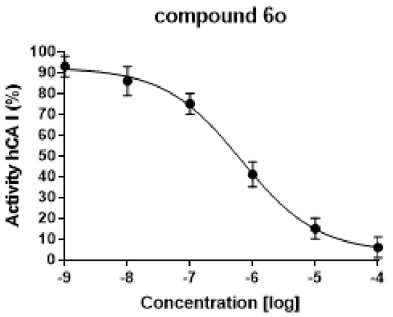
compound 6o
Activity hCA I (%)
Concentration [log]

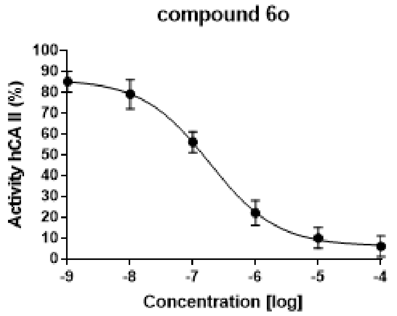
compound 6o
Activity hCA II (%)
Concentration [log]

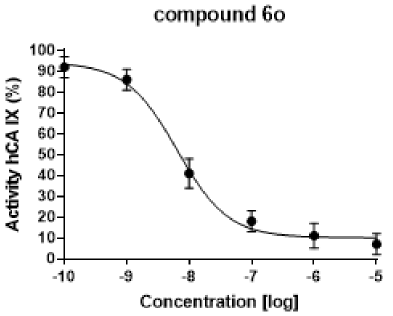
compound 6o
Activity hCA IX (%)
Concentration [log]

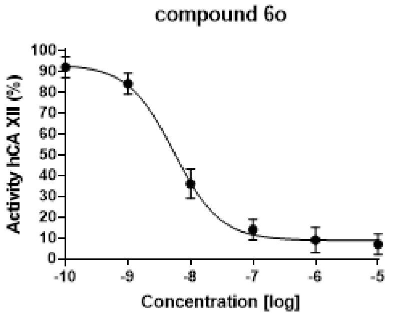
compound 6o
Activity hCA XII (%)
Concentration [log]

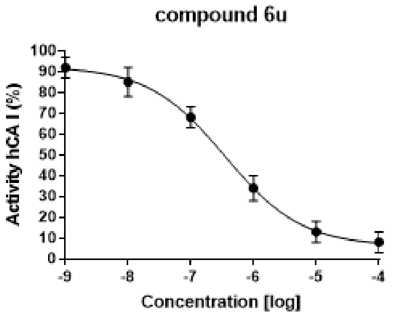
compound 6u
Activity hCA I (%)
Concentration [log]

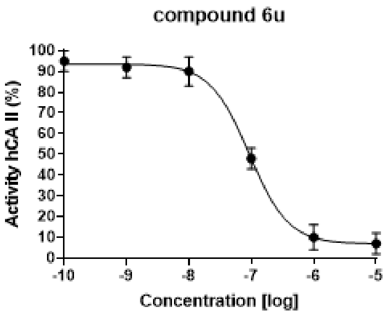
compound 6u
Activity hCA II (%)
Concentration [log]

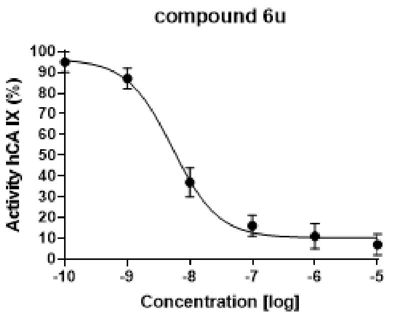
compound 6u
Activity hCA IX (%)
Concentration [log]

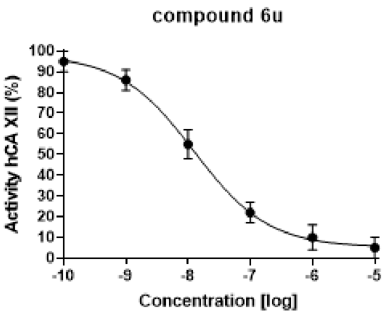
compound 6u
Activity hCA XII (%)
Concentration [log]
